# RNA-seq as a tool for evaluating human embryo competence

**DOI:** 10.1101/gr.252981.119

**Published:** 2019-10

**Authors:** Abigail F. Groff, Nina Resetkova, Francesca DiDomenico, Denny Sakkas, Alan Penzias, John L. Rinn, Kevin Eggan

**Affiliations:** 1 Department of Stem Cell and Regenerative Biology, Harvard University, Cambridge, Massachusetts 02138, USA;; 2 Department of Systems Biology, Harvard Medical School, Boston, Massachusetts 02115, USA;; 3 Boston IVF, Waltham, Massachusetts 02451, USA;; 4 Division of Reproductive Endocrinology and Infertility, Department of Obstetrics and Gynecology, Beth Israel Deaconess Medical Center, Boston, Massachusetts 02215, USA;; 5 Obstetrics, Gynecology, and Reproductive Biology, Harvard Medical School, Boston, Massachusetts 02215, USA;; 6 Department of Biochemistry, BioFrontiers, University of Colorado Boulder, Boulder, Colorado 80301, USA

## Abstract

The majority of embryos created through in vitro fertilization (IVF) do not implant. It seems plausible that rates of implantation would improve if we had a better understanding of molecular factors affecting embryo competence. Currently, the process of selecting an embryo for uterine transfer uses an ad hoc combination of morphological criteria, the kinetics of development, and genetic testing for aneuploidy. However, no single criterion can ensure selection of a viable embryo. In contrast, RNA-sequencing (RNA-seq) of embryos could yield high-dimensional data, which may provide additional insight and illuminate the discrepancies among current selection criteria. Recent advances enabling the production of RNA-seq libraries from single cells have facilitated the application of this technique to the study of transcriptional events in early human development. However, these studies have not assessed the quality of their constituent embryos relative to commonly used embryological criteria. Here, we perform proof-of-principle advancement to embryo selection procedures by generating RNA-seq libraries from a trophectoderm biopsy as well as the remaining whole embryo. We combine state-of-the-art embryological methods with low-input RNA-seq to develop the first transcriptome-wide approach for assessing embryo competence. Specifically, we show the capacity of RNA-seq as a promising tool in preimplantation screening by showing that biopsies of an embryo can capture valuable information available in the whole embryo from which they are derived. Furthermore, we show that this technique can be used to generate a RNA-based digital karyotype and to identify candidate competence-associated genes. Together, these data establish the foundation for a future RNA-based diagnostic in IVF.

Because of rising rates of infertility and greater societal acceptance of the involved technologies, in vitro fertilization (IVF) is becoming more commonplace. Technical advances in retrieval methods, culture systems, and embryo vitrification have permitted significant improvements in pregnancy success rates since the first cases (Wang and Sauer 2006). Although there has been extensive study of preimplantation embryos, only a cursory understanding of the transcriptional control of human preimplantation development currently exists. Indeed, to date, RNA-sequencing (RNA-seq), which allows comparison to clinically relevant embryological features, has not been performed. As a result, it is unknown how alterations in transcript abundance contribute to establishing a developmentally competent embryo (i.e., one that is capable of implanting and establishing a viable pregnancy).

The molecular factors contributing to a developmentally competent human embryo are only beginning to be explored. It is known that even euploid embryos transferred into a normal uterus fail to implant 30%–50% of the time (Dahdouh et al. 2015; Setton et al. 2015; Whitehead et al. 2017; Simon et al. 2018). Although some of this may be because of the endometrium, other uterine pathology, and potential paternal factors, there is another possible explanation for this imperfect efficiency even when all available testing is confirmatory: the shroud of mystery surrounding the molecular events of preimplantation development, particularly those that establish embryo competence. Currently, it is unclear at which stage competence is established. Three of the earliest checkpoints are (1) progression past zygote stage to cleavage and compaction stages, (2) further progression to the blastocyst stage, and (3) implantation. The decision to progress through these stages or senesce, however, is unlikely to rely on a single event at a single stage. Because of the many likely contributing factors, establishing robust predictors of embryo competence has remained a challenge.

Currently, efforts to judge embryo competence are largely subjective. The most common is morphological assessment using an established embryologic grading system (Gardner et al. 2013). In some cases, additional data, such as embryo developmental morphokinetics or such as karyotype status from preimplantation genetic testing for aneuploidy (PGT-A), are also used to help select an embryo (Kovacs et al. 2013; Pérez et al. 2014; Aparicio-Ruiz et al. 2016; Sekhon et al. 2017). PGT-A yields a digital karyotype, including sex chromosome content. Aneuploid embryos are generally considered incompatible with life (with exceptions of Chromosomes X, Y, 13, 18, and 21), and thus, these embryos are not typically transferred (Hassold and Jacobs 1984; Robinson et al. 2001). Additionally, diagnosing embryo sex chromosome content is useful in avoiding the selection of embryos with X- or Y-linked traits (Wang and Sauer 2006). However, none of these approaches fully predicts implantation or developmental competence.

Full-transcriptome RNA-seq provides high-dimensional information about a sample (Mortazavi et al. 2008; Trapnell et al. 2010). The expression levels of key genes can be informative and even predictive of cellular states. Recently, advances in library preparation techniques have allowed the application of RNA-seq technologies to small inputs, including single cells (Tang et al. 2009; Islam et al. 2011; Ramsköld et al. 2012; Picelli et al. 2014). Multiple studies have pioneered the application of single-cell RNA-seq technology to the human preimplantation transcriptome (Xue et al. 2013; Yan et al. 2013; Blakeley et al. 2015; Petropoulos et al. 2016). These studies, however, are generally focused on early transcription networks or lineage delineations and do not report indicators of embryo quality or chromosomal status such as karyotype. As a result, these data have not been useful for the assessment of clinically relevant gene expression that relates to potential developmental outcomes. Indeed, transcriptional trends ascertained without regard to embryo quality may differ from those obtained from the same cell type in embryos that pass clinical evaluation. For example, poorly formed or apoptosing trophectoderm (TE) would be expected to express different genes than well-formed TE capable of implantation.

We reasoned that the information content available from RNA-seq, if properly used, can bridge the gap in our understanding of early molecular events in human development and provide a more comprehensive metric for grading embryo competence. Here, we performed several methods of embryo evaluation (morphological, morphokinetic, and genetic screening) along with RNA-seq for the first time. Specifically, we have performed a series of experiments wherein the RNA-seq of embryos with varying embryological scores were compared to identify candidate biomarkers of developmental competence. Additionally, we have performed RNA-seq of TE samples acquired using clinical biopsy techniques in order to compare the information content of these samples to the phenotype and karyotype of the remaining whole embryo (WE).

Our study benchmarks the ability of RNA-seq to identify embryo sex chromosome content and karyotype. It also provides a proof-of-principle data set that combines morphologic, morphokinetic, and embryo karyotype status with whole-transcriptome data to identify candidate genes associated with metrics of embryo competence. These findings can inform the design of future clinical studies to evaluate the utility of low-input RNA-seq of TE biopsies to make clinical decisions about which embryo to implant as part of assisted reproductive treatment.

## Results

To establish a proof-of-principle demonstration that RNA-seq might one day be a useful tool in preimplantation genetic screening, our aim was threefold: (1) to evaluate the information content available in the full transcriptome of a WE or biopsy taken at the blastocyst stage; (2) to assess the fidelity of a TE biopsy to report information content of a WE; and (3) to correlate transcriptomic profiles from embryo RNA-seq to well-known embryo selection metrics (morphological grading, morphokinetic grading, and karyotype).

### Generation of a unique data set to assess use of RNA-seq in embryo evaluation

**Figure 1.**
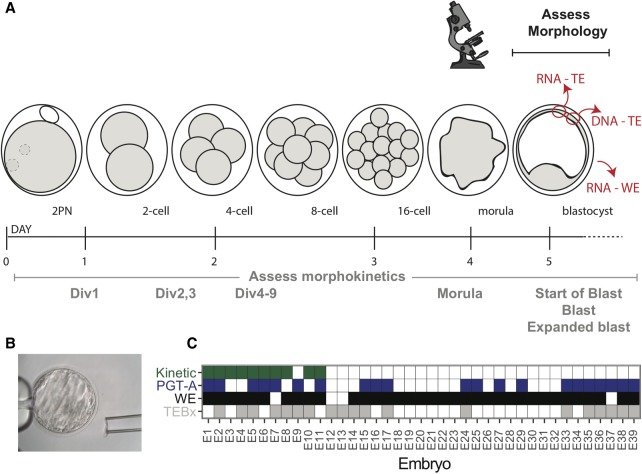
Experimental overview. (*A*) Preimplantation human development time-course depicting our comparative analytical approach. Samples were processed from blastocyst stage embryos and assessed for morphokinetic criteria and morphology before biopsy. One trophectoderm (TE) biopsy was processed for DNA-based preimplantation genetic testing for aneuploidy (PGT-A), one was harvested for RNA-seq, and the remaining whole embryo (WE) was also processed for RNA-seq. (*B*) Representative image of a blastocyst. (*C*) Data overview table. Embryos (E1-39) for which we have morphokinetic data are shaded in green; those for which DNA-based PGT-A yielded a result are depicted in blue; and those for which we have RNA-seq of either WE or TE biopsy are labeled in black and gray, respectively.

Embryos donated under an institutional review board (IRB)–approved protocol were thawed, cultured, graded for morphological quality, and double TE biopsied at the blastocyst stage (for details, see Methods) (Fig. 1A,B). One biopsy was sent for DNA-based PGT-A, using next generation-like sequencing performed by an established regional genetics laboratory (Invitae) (Fig. 1A; Kinde et al. 2012). The second TE biopsy and the remaining embryo were harvested for RNA-seq library preparation using the Smart-seq2 protocol (Picelli et al. 2014). Images were obtained every 20 min throughout the course of embryo development. Thus, for a subset of embryos donated at the zygote stage, we were able to gather morphokinetic data (the timing of the first cell divisions, starting at the fusion of the two pronuclei). The resulting data set includes 35 WE samples, 19 TE biopsies, 21 PGT-A results, and 10 morphokinetic grades representing 39 unique embryos (Fig. 1C). Each RNA-seq library was sequenced to an average depth of approximately 44.6 million reads and assessed for quality (note that three TE biopsies were excluded after quality control; see Methods).

Because TE biopsies consist typically of three to seven cells whereas WEs post-biopsy are expected to consist of between 60 and 100+ cells, we anticipated that gene expression would differ greatly between each sample type. Indeed, we found that samples largely separated by sample type (WE or TE) in principle component analysis (PCA) (Supplemental Fig. S1A). We also inferred that transcriptome representation may differ because of both biological and technical sources, such as precise developmental stage of the blastocyst, and variation in the efficiency of library preparation. To assess the relationship of library complexity to expression, we plotted the number of expressed genes (Supplemental Fig. S1B,C) and transcriptome coverage of each sample (Supplemental Fig. S1D), as well as the gene count distribution across sample types (Supplemental Fig. S1E). We found that although WEs did indeed tend to express more genes than biopsies, the skew was not extreme. Samples with lower transcriptome coverage did not have a higher incidence of expression “jackpotting,” in which one or a few genes are overamplified during library preparation, and thus disproportionately contribute to the library, drowning out signal from more lowly expressed genes (Supplemental Fig. S1F; Levin et al. 2010). Finally, we overlaid read count information on the PCA, revealing that total RNA count weight per sample does not drive the separation between WE and biopsy (Supplemental Fig. S1G). Indeed, the lower gene count in the biopsies may reflect biology rather than a lighter sampling of the transcriptome owing to lower input in library preparation. For instance, the WE contained both TE and inner cell mass (ICM), whereas the biopsy contained only TE; thus, we expected more diverse cell types and potentially more genes in the WE than the biopsy.

### RNA-seq accurately reports the sex chromosome content of an embryo

Next, we sought to use RNA-seq to determine the sex chromosome content of WEs at the blastocyst stage. Although some previous studies suggest that the classical Y-linked gene *SRY*, commonly used in genotyping assays, is expressed in preimplantation human embryos (Ao et al. 1994; Fiddler et al. 1995), we found that it is not accumulated to be detected by RNA-seq (Supplemental Fig. S2A), consistent with previous analysis of RNA-seq at this stage (Petropoulos et al. 2016). Similarly at this time, *XIST*, the X-linked gene that is responsible for dosage compensation and that is commonly used to identify females in genotyping assays (Disteche and Berletch 2015), is expressed in both XX and XY embryos (Supplemental Fig. S2A; Daniels et al. 1997; Nichols et al. 2009; Briggs et al. 2015; Petropoulos et al. 2016; Sousa et al. 2018). To overcome these single-gene genotyping limitations, we assessed expression of many sex chromosome–linked genes, excluding genes from the sex chromosome pseudoautosomal regions 1 and 2 (containing genes that can map to either the X or Y Chromosome) (Mangs and Morris 2007).

**Figure 2.**
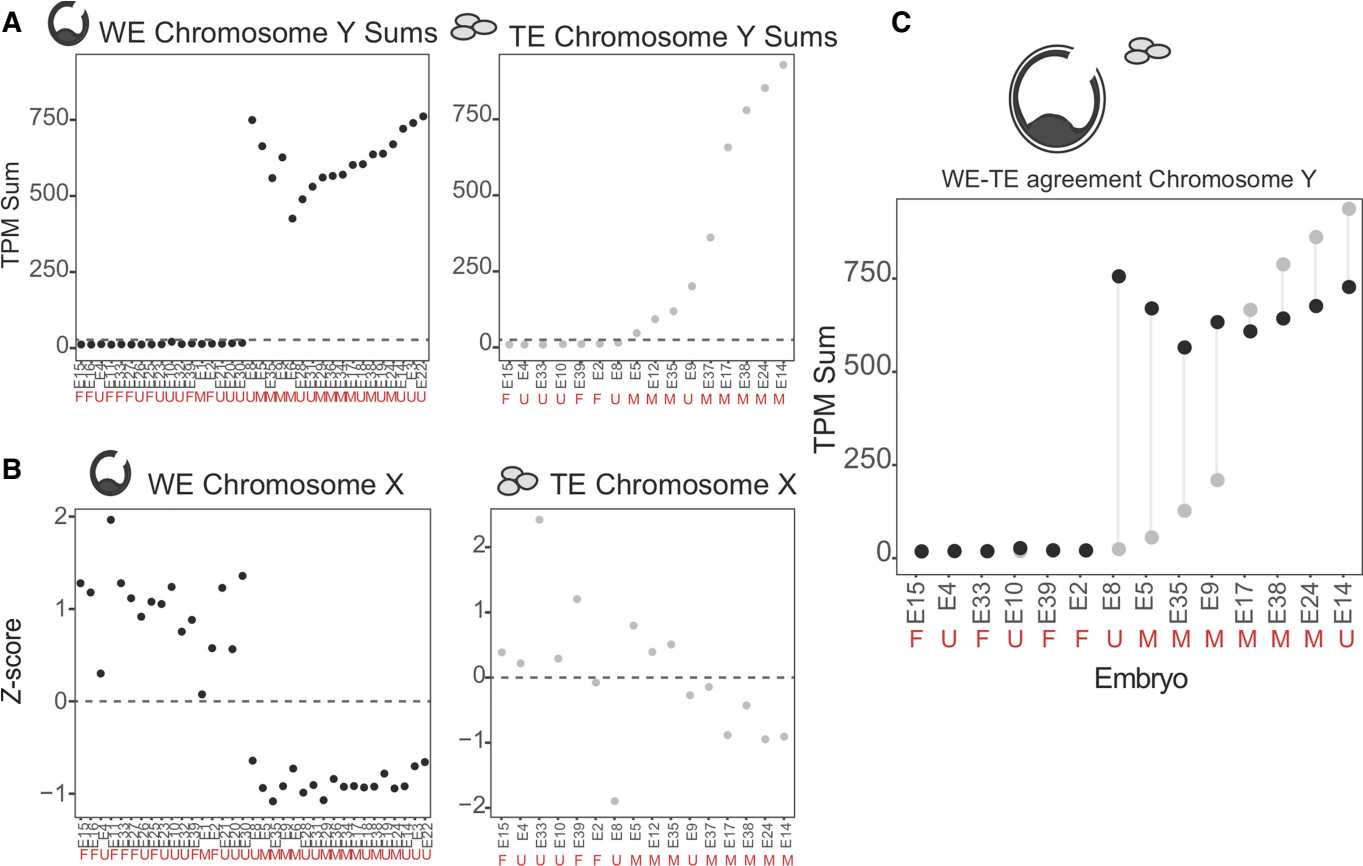
RNA-based embryo sex chromosome content. (*A*) Chromosome Y–specific gene TPM sums for all WEs (black; *left*) and TE biopsy samples (gray; *right*), respectively. Embryos are on the *x*-axis and are ordered by Chromosome Y TPM sum. Dashed line indicates sum of 25 TPM threshold for evidence of a Y Chromosome in the sample. Red letters indicate sex chromosome status as determined by DNA-based PGT-A: *M* = XY; *F* = XX; *U* = Undefined. (*B*) Chromosome X *Z*-score profiles for all WE and TE biopsy samples, respectively. (*C*) Chromosome Y TPM sums for paired WE–TE samples (from the same embryo). Red letters indicate PGT-A results. Black dot indicates WE sample; gray dot, TE sample.

We reasoned that Y Chromosome genes should be expressed >1 TPM in approximately half of our samples (corresponding to XY embryos). Upon examination of sequencing data, three protein-coding genes fit these criteria (*DDX3Y*, *RPS4Y1*, and *EIF1AY*). To assess presence or absence of the Y Chromosome, we summed TPMs across these three genes and observed that a sum >25 TPM was a likely indicator for the presence of a Y Chromosome (Fig. 2A; Supplemental Fig. S2B). By using this cut-off, we found a clear separation between XX and XY samples in WEs (Fig. 2A, right). We compared this approach to paired PGT-A data when available and found that we identified Y-containing embryos 18/19 times (94.7%; 19 WE samples with paired PGT-A). The sole mismatch, E1, was identified as XY via PGT-A but contained no evidence for Y Chromosome reads in the RNA-seq. The TE biopsy results were less clearly delineated (Fig. 2A, left). However, they agreed with PGT-A–identified sex chromosome karyotype 11/11 times (100%). A subset of embryos (E5, E12, E35, E9) contained reads suggesting the presence of a Y Chromosome, albeit at much lower levels than the average XY biopsy (520 TPMs is the mean sum for embryos with XY karyotype by PGT-A). These results could indicate that one or more cells in the biopsy has lost the Y Chromosome whereas at least one cell retained it. When cells of the same embryo have different chromosomal contents, the embryo is considered a karyotype mosaic. Collectively, these data indicate that the sum of Y-specific expressed genes can indicate presence or absence of the Y Chromosome, as suggested previously (Petropoulos et al. 2016).

Although summing specific genes worked well for a single-copy chromosome such as the Y, this approach was not expected to work for the X Chromosome, which is present in at least one copy in all embryos, and so we cannot use a presence-or-absence approach. To estimate how many copies of X were present in a given sample, we summed counts across all expressed genes on the X and generated a *Z*-score for chromosome-wide expression across our WE samples (see Methods; Fig. 2A,B; Wang et al. 2002; Kinde et al. 2012; Moreira de Mello et al. 2017). Visual inspection of the *Z*-score plots proved a reliable method of estimating sex chromosome content in WEs. We called samples with negative Chromosome X *Z*-scores (and Y sums >25 TPM) “XY” and those with positive Chromosome X *Z*-scores (and no evidence of a Y Chromosome) “XX” (Fig. 2A,B). We again compared these inferred sex chromosome calls to the PGT-A results and found that they agree perfectly with the Y-sum approach above, including for the discordant E1. Thus, independent evaluation of expression from both X and Y Chromosomes indicates that the E1 embryo is likely XX and not XY as suggested by PGT-A. These results indicate that both summation of Y-specific gene TPMs and sex chromosome *Z*-score are reliable methods for determining sex chromosome status of an embryo at the blastocyst stage.

Encouraged by these results, we next sought to assess the ability of the *Z*-score approach to estimate copy number of the X Chromosome in the TE biopsy, despite the activation of dosage compensation starting around this time. X-Chromosome inactivation (XCI) down-regulates expression from the X, which we hypothesized may interfere with our ability to infer copy number using RNA read counts (Petropoulos et al. 2016). Indeed, we found that these digital karyotyping results were less clearly delineated than in WEs, agreeing with PGT-A 7/11 times and disagreeing four times, consistent with our concern about XCI. As such, we called sex chromosome status based solely on the Chromosome Y sums.

A major limitation of biopsy-based approaches is that they, by definition, sample only a small portion of the embryo (∼5%). We wanted to understand to what extent a biopsy recapitulated the information content of the remaining WE. To assess how accurately a biopsy reported on the sex chromosome status of a WE, we analyzed just the 14 samples passing quality control for which we had RNA-seq from both the WE and biopsy (TE–WE pairs) (Fig. 2E). We found that our sex chromosome content calls based on TPM sums from the Y Chromosome agreed between WE and biopsy 13/14 times and disagree once. In the discordant case, the biopsy indicates a total loss of the Y Chromosome, suggesting embryonic karyotype mosaicism. If an embryo is mosaic and the biopsy samples cells with different sex chromosome content than the remaining embryo, the biopsy could over- or underestimate the reads associated to that chromosome. Each of the low Y-sum biopsies reported in Figure 2A (E5, E12, E35, E9, E37; mismatching in Fig. 2C: E8, E5, E35, E9) is consistent with Y Chromosome loss in the biopsy. Consistent with this notion, it is well known that Y Chromosome loss is one of the most commonly occurring mitotic errors (Eggan et al. 2002; Duijf et al. 2013; Forsberg 2017; Loftfield et al. 2018).

Finally, we performed differential gene expression analyses between WEs with XX and XY karyotypes using the sex chromosome calls ascertained above. We identified 194 significantly differentially expressed genes of which 146 are sex-linked (Supplemental Fig. S2C; Supplemental Files S2, S3). We further refined this list by selecting those with a log_2_ fold change >2 and visualized their log_2_-transformed expression values in a heatmap (Supplemental Fig. S2D). Because the column dendrogram separates XX from XY WEs as in Figure 2A, these 88 genes may, in aggregate, constitute a suitable sexing gene list, similar to the approach taken above. We include the results of this analysis as Supplemental File S3.

Together, our data indicate that RNA-seq from either WEs or TE biopsies can be used to detect embryo sex chromosome content and that, like any other biopsy method, our results can be limited by the occasional confounding factor of mosaicism.

### RNA-seq can provide a digital karyotype

Our analysis of sex chromosomes indicated that RNA-seq can be used to infer sex chromosome status of an embryo by calling the presence or absence of a single-copy chromosome such as the Y in conjunction with a *Z*-score assessment of the X Chromosome. We reasoned that a similar analysis may enable us to infer dosage of autosomes as well. We therefore extended our *Z*-score approach to the generation of a RNA-based digital karyotype for all autosomes.

**Figure 3.**
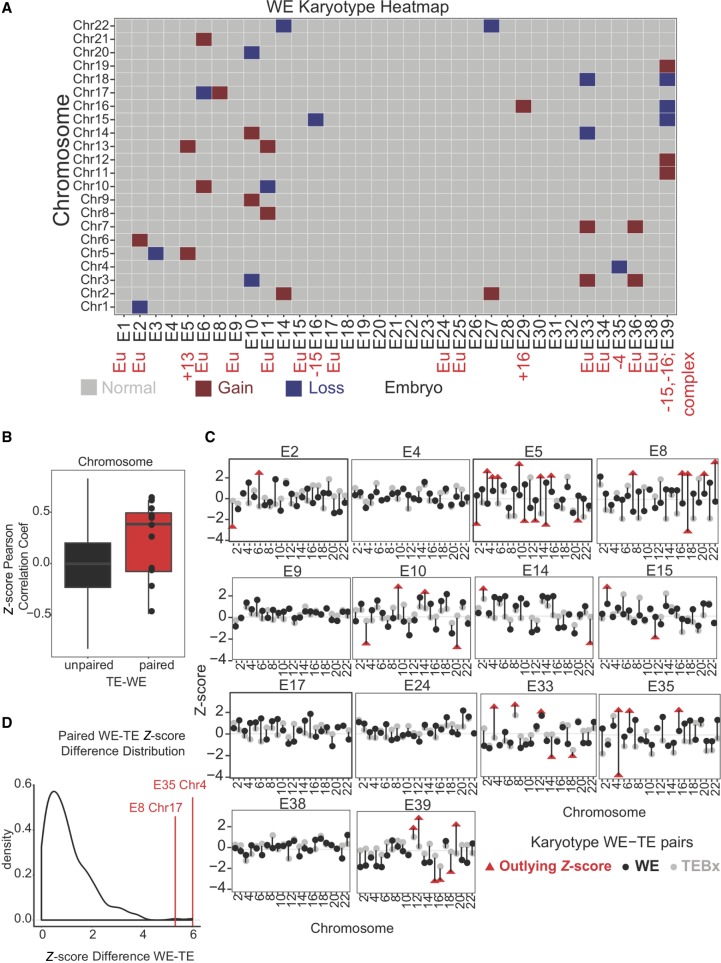
RNA-based digital karyotype. (*A*) Overview of WE digital karyotypes. Embryos are on the *x*-axis and chromosomes are on the *y*-axis. Gray indicates *Z*-score within the normal range (±2); red, a gain (>2 *Z*-score); and blue, a chromosomal loss (<−2 *Z*-score). Red text indicates results of DNA-based PGT-A where applicable. (*B*) Boxplot of Pearson correlation coefficients comparing *Z*-score profiles from unpaired (black; from different embryos) or paired (red; from the same embryo) WE and TE samples. (*C*) *Z*-score profiles for paired WE and TE biopsy samples. Black dot indicates *Z*-score (*y*-axis) of WE; gray dot, *Z*-score of TE biopsy. Red triangles indicate outlying *Z*-scores. Chromosomes indicated along the *x*-axis. (*D*) Distribution of *Z*-score differences between WE and TE biopsy for paired samples (originating from the same embryo). Red lines indicate E8 Chromosome 17 and E35 Chromosome 4 as outliers.

Briefly, *Z*-scores were calculated on total read counts per chromosome per sample type, and outliers were highlighted as chromosome scores >2 SDs away from the mean (Fig. 3A; Supplemental Fig. S3A). This outlier call allowed us to quickly assess potential instances of aneuploidy with great sensitivity, which would be a prerequisite for identifying all potential cases of aneuploidy in a future clinical test. By using this approach, we identified 20 potential chromosomal gains (approximating a trisomy) and 15 potential chromosomal losses (approximating a monosomy) across 15 unique embryos, suggesting a 15/35 (43%) aneuploidy-affected rate at blastocyst stage for our WEs. This method identified every instance of autosomal aneuploidy reported in our embryo cohort by PGT-A (E5 + 13, E16 − 15, E29 + 16, E35 − 4, E39 − 15,−16). In addition, our RNA-based method identified additional candidate instances of aneuploidy in whole blastocysts compared with PGT-A, suggesting either that our digital RNA-seq karyotyping was more sensitive or had a higher false-positive rate or that mosaicism led to there being more karyotypic changes in cells of the WE, tested here, relative to the biopsy. For more information including assessment of outlier call significance, see the Methods.

Next, we sought to assess the extent to which the additional instances of aneuploidy reported above might depend on method of sample preparation (i.e., RNA-seq vs. PGT-A). To this end, we examined whether or not there was strong concordance between RNA digital karyotypes generated from WEs and TE biopsies. To start, we wanted to assess how well a TE biopsy recapitulated expression trends from the WE at the chromosome level, given that the WE contains more cell types than the biopsy. To do this, we performed a correlation analysis. In this analysis, we assessed how the distribution of *Z*-scores in each biopsy compared with the *Z*-score distribution in every WE using the Pearson correlation coefficient. We then compared the distribution of correlation coefficients between samples that came from the same embryo (“paired”) or samples that came from separate embryos (“not paired”). We found that indeed, paired TE–WE samples were more closely correlated than their “not paired” counterparts (*P* = 0.01553) (Fig. 3B). This indicated that RNA-seq of a TE biopsy can yield reliable information about the rest of the embryo from which it was taken.

To delve deeper into the relationship between WE and TE biopsy karyotypes, we next assessed how well the *Z*-scores for each chromosome agreed between both samples. To this end, we compared the *Z*-scores for each chromosome from the 14 WE and TE biopsy pairs by calculating the difference between each *Z*-score pair (referred to as “*Z*-score distance”) (Fig. 3C,D). Broadly speaking, we found good agreement for each pair, as indicated by most of the *Z*-score distances being quite small (most distances are <2) (Fig. 3D). By observing each pair of karyotypes across all chromosomes per embryo, we found that for 5/14 of these embryos, both samples agree that the embryo is euploid (i.e., all *Z*-scores lie within 2 SDs of zero; E4, E9, E17, E24, E38). For another 7/14 embryos, at least one chromosome in one sample is a *Z*-score outlier, indicating possible karyotype mosaicism. It is interesting to note that many of these outlier *Z*-scores were in the WE, indicating that the WE had an aneuploidy whereas the TE biopsy was euploid. These results lend further support to the notion that the karyotype statuses of the biopsy and WE systematically differ because of mosaicism. Indeed, the final 2/14 pairs provide evidence of mitotic nondisjunction. Mitotic nondisjunction occurs when sister chromatids fail to separate during mitosis, resulting in reciprocal aneuploidies in the daughter cells. In other words, one daughter cell has a trisomy for the affected chromosome, and the other has a monosomy. In samples E8 and E35, we found clear evidence of reciprocal aneuploidy, in which one chromosome was an outlier in both sample types but in the opposite direction (Fig. 3C,D). These findings underscore the presence and at least one source of mosaicism in our embryo cohort.

Given the multiple approaches by which we have identified evidence of mosaicism across our data set, we sought to estimate the total rate of mosaic embryos in our cohort. We considered the embryos for which we have multiple karyotype results (either from RNA-seq or PGT-A) after quality control and considered Y-loss evidence from PGT-A or discordant outlier calls between TE and WE samples to be evidence of mosaicism (22 embryos) (Supplemental Table S1). By using this conservative approach, we estimated that 5/22 embryos (22.7%) were mosaic. We also calculated a more permissive mosaicism rate by considering any mismatched karyotype between PGT-A and the WE RNA-karyotype as evidence of mosaicism, which yielded a mosaicism rate of 50% (11/22 embryos). Both estimates are consistent with mosaicism rates reported in the literature, which range from 15%–90% (van Echten-Arends et al. 2011). Our conservative estimate almost exactly matches the percentage of mosaic embryos from a large cohort of blastocysts that underwent PGT-A via DNA-seq, the most appropriate technical comparison to our cohort (21.8%) (McCoy 2017; Munné et al. 2017).

Together, our data indicate that RNA-seq can be used to generate a digital karyotype from preimplantation human embryos and that, despite the potential complicating factor of mosaicism, TE biopsies can be predictive of both the expression trends and chromosomal content of WEs.

### Correlation of expression trends with established competence measures

To assess the difference in transcriptomic profiles between WEs showing different grades by a variety of established developmental competence metrics, we collected RNA-based digital karyotypes (Fig. 3A), as well as morphological or morphokinetic grades for as many embryos as possible. Here we present three vignettes summarizing the trends that differentiate potentially competent from likely incompetent embryos across three independently assessed axes: karyotype status, morphological grade, and morphokinetics.

**Figure 4.**
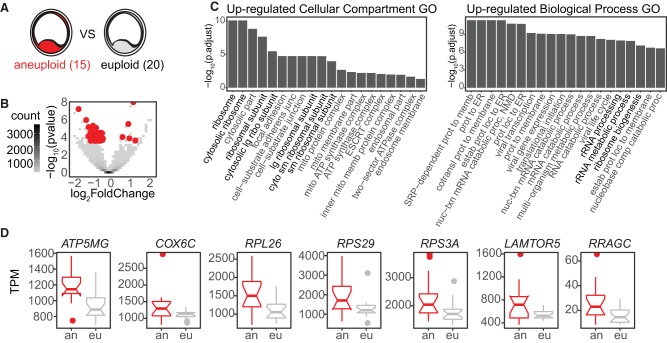
RNA-based karyotype-associated gene sets. (*A*) Schematic depicting RNA-based karyotype differential expression analysis: 15 aneuploid (by RNA) versus 20 euploid WE samples. (*B*) Volcano plot of differentially expressed genes from RNA-based karyotype differential expression analysis. Red indicates significant differential expression (BH-adjusted *P*-value <0.05). (*C*) Gene Ontology (GO) enriched terms for genes up-regulated in the aneuploid samples: (*left*) cellular compartment; (*right*) biological process. (*D*) Boxplots of selected differentially expressed genes from the RNA-based karyotype differential expression analysis (BH-adjusted *P*-value <0.05). Aneuploid samples are in red; euploid samples, in gray.

To assess the relationship between aneuploidy and transcriptome profile, we performed an unbiased differential gene expression analysis using DESeq2 on all aneuploid (15) and euploid (20) WE samples (Fig. 4A) in our data set as identified in Figure 3A (Love et al. 2014). Note that in segmenting our samples this way, we ignored all other characteristics such as embryo sex, PGT-A result, or morphological or morphokinetic grade. We found five genes down-regulated and 48 genes up-regulated in the aneuploid samples compared with the euploid samples (53 total differentially regulated genes) (Fig. 4B).

To identify pathways among the 53 differentially expressed genes, we performed a Gene Ontology (GO) analysis of the up- or down-regulated groups (Fig. 4C). We found that genes up-regulated in the aneuploid samples were enriched for annotations, including ribosomal RNA processing and biogenesis, and mitochondrial membranes. We highlight seven of the genes significantly up-regulated in aneuploid samples (Fig. 4D). These include a mitochondrial ATP synthase (ATP synthase membrane subunit g, *ATP5MG*) and cytochrome c oxidase subunit 6C (*COX6C*), which encodes the terminus of the mitochondrial electron transport chain; ribosomal protein components of the 60S and 40S subunits (*RPL26*, *RPS29*, *RPS3A*); and components of the RAG and RAGULATOR complexes (*LAMTOR5* and *RRAGC*), involved in regulating cell growth through mTOR complex 1 signaling (Sekiguchi et al. 2001; Marusawa et al. 2003; Bar-Peled et al. 2012; Long et al. 2016). *LAMTOR5* is additionally implicated in control of apoptosis (Marusawa et al. 2003). Together, these categorical enrichments and gene highlights suggest a change in metabolic pathways in aneuploid embryos. Another explanation for this finding is that incorrect stoichiometry of protein complexes that are generated from genes residing on aneuploid chromosomes, including mitochondrial subunits, leads to metabolic dysfunction.

Given the presence of genes associated with mitochondrial function in this gene list, we assessed the relationship between karyotype status and expression of genes from the mitochondrial genome (excluded from the prior analysis owing to their high expression). We plotted TPM values associated with each mitochondrial gene for each WE or TE sample and found a general trend whereby euploid samples have an elevated median expression in both the TE and WE samples (Supplemental Fig. S4). We performed *t*-tests for each comparison but found that only one gene, *MT-ND5*, has significantly higher expression in the euploid WE samples (*P* = 0.009953).

This general trend could indicate that as the nuclear genome loses transcriptional control in response to aneuploidy, mitochondria respond by altering regulation of specific complexes to adapt. This regulation could occur independently of the mitochondrial DNA (mtDNA) copy number, a proposed biomarker of embryo competence, which is an area of active investigation in the field (Fragouli et al. 2015; Treff et al. 2017). As a result, we find that transcriptional output of mitochondrially encoded *MT-ND5*, part of the Mitochondrial complex I, is reduced in aneuploid embryos. Although this alone is not enough to be a diagnostic marker, it provides a trend that could be a useful component of an RNA-seq diagnostic.

**Figure 5.**
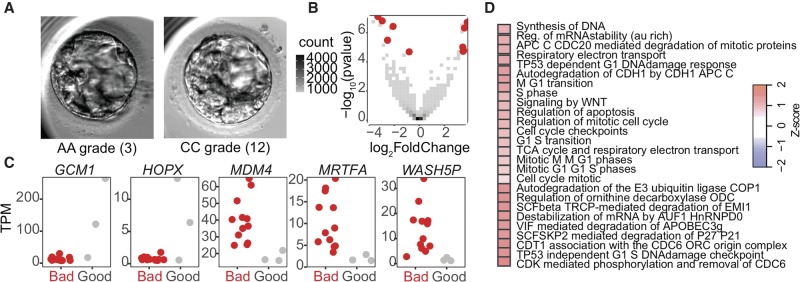
Morphology-associated gene sets. (*A*) Representative good morphological quality (AA grade on Gardner scale) and bad morphological quality (CC grade on Gardner scale) blastocysts from our cohort. (*B*) Volcano plot of differentially expressed genes from morphology-based differential expression analysis. Red indicates significant differential expression (BH-adjusted *P*-value <0.05). (*C*) Jitter plots of selected significant differentially expressed genes from the morphology-based differential expression analysis (adjusted *P*-value <0.05). (*D*) Reactome gene set enrichment analysis of gene expression from morphology-based differential expression analysis. Red indicates increase in gene set expression in “good” samples.

Next, we assessed transcriptome-wide differences according to morphological grade at blastocyst stage. The Gardner grading system was used to evaluate embryos (Gardner and Schoolcraft 1999; Gardner et al. 2000). By using this alphanumeric system, the blastocyst was given a number depending on its expansion status (1–6), and both the ICM and TE were assigned a grade between A and C according to their quality (i.e., cell cohesion, uniformity, and viability) (Fig. 5A). We compared only the highest-quality embryos, AA (in which both ICM and TE are grade A, regardless of expansion status), to those with the lowest-quality score, CC. It is notable that embryos of CC grade would typically not be selected for transfer or freezing in clinical practice, especially if better-quality embryos are available.

By using DESeq2 to perform an unbiased differential gene expression analysis as above, we found five genes up-regulated and five genes down-regulated in the morphologically aberrant samples (Fig. 5B). Although no GO categories were enriched in either the up- or down-regulated genes, a number of biological themes emerged from the identities of the differentially expressed genes themselves. First, *GCM1* and *HOPX*, two of the genes up-regulated in WEs with good morphology, are implicated in control of placental growth factor or trophoblast differentiation, respectively (Fig. 5C; Chen et al. 2004; Bainbridge et al. 2012). Additionally, HOPX interacts with serum response factor (SRF), which is known to regulate many immediate early response genes (Yamaguchi et al. 2009). We also found SRF-related genes highly up-regulated in embryos of poor morphological quality, as detailed below (Fig. 5C). *MDM4*, *MRTFA*, and *WASH5P* were each highly up-regulated in embryos of poor morphology and are each in some way implicated in cancer. MDM4 is a TP53-binding protein that suppresses TP53's apoptotic functions and is overexpressed in cancer (Steinman et al. 2005). MRTFA is associated with acute megakaryocytic leukemia, acts as a coactivator of SRF, and inhibits activation of caspases, which suppresses apoptosis (Sun et al. 2006). Finally, *WASH5P* is a pseudogene that is differentially methylated in early-stage breast cancer (Titus et al. 2017). Gene set analysis (GSA) of this differential comparison reinforced the trends highlighted above: Tumor suppressor gene sets, such as those implicating TP53 damage control, are up-regulated in embryos with good morphological grades (Fig. 5D). Together, these data suggest that embryos with poor morphological scores show a lack of transcriptional control reminiscent of the hallmarks of cancer, and also implicate SRF signaling in establishing the transcriptional conditions necessary for proper morphological development.

**Figure 6.**
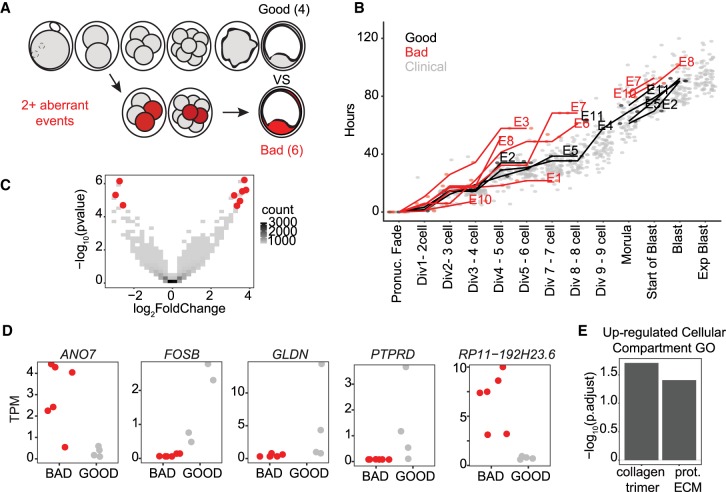
Morphokinetic quality-associated gene sets. (*A*) Schematic indicating the morphokinetic differential analysis (six low-quality vs. four high-quality embryos). Low quality is defined as deviance by >1 SD from clinical standard in two or more measurements. (*B*) Comparison of morphokinetic data from embryos in this analysis (red indicates low-quality embryos; black, high quality) to clinical-standard embryos (gray): *y*-axis is time in hours; *x*-axis, sequential series of divisions and other embryological events measured from the time of pronuclei fade. (*C*) Volcano plot of differentially expressed genes from morphokinetic-based differential expression analysis. Red indicates significant differential expression (BH-adjusted *P*-value <0.05). (*D*) Jitter plots of selected significant differentially expressed genes from the morphokinetic-based differential expression analysis (adjusted *P*-value <0.05). (*E*) Cellular compartment GO analysis from genes up-regulated in embryos that meet clinical standards for morphokinetics.

Finally, we assessed transcriptome differences at the blastocyst stage associated with varying scores of morphokinetic development. Embryos donated at the zygote stage were observed developing to blastocyst stage, and the timing of a series of events corresponding to particular cellular divisions (two-cell, three- and four-cell, five- to eight-cell, morula, early blastocyst, well-developed blastocyst) was measured and compared with a large set of clinical-grade embryos resulting from fertilization of donor eggs (for details, see Methods) (Fig. 6A). Briefly, a clinical-standard timeline was established for attainment of each developmental stage by calculating the mean and SD of the clinical donor-egg–derived samples, and each research embryo was compared against this timeline. An embryo was determined to be morphokinetically aberrant if its development differed by greater than 1 SD twice or more during the time-course. In total, we were able to assign scores to 10 embryos: six aberrant and four that were in line with clinical-standard samples (Fig. 6A,B).

By using DESeq2, we compared transcriptional profiles of embryos with high- and poor-quality morphokinetics and found three genes down-regulated and six up-regulated in samples meeting strong morphokinetic standards (Fig. 6C). Two of the three down-regulated genes are uncharacterized—*AC013268.5* (*ENSG00000283684*) and *RP11-192H23.6* (*ENSG00000265287.2*), whereas *ANO7* is an androgen-responsive gene associated with prostate cancer (Fig. 6D; Bera et al. 2004; Pedemonte and Galietta 2014). A large number of cellular compartment GO categories were enriched among the six up-regulated genes, including collagen trimer and proteinaceous extracellular matrix (ECM) (Fig. 6E). Upon further examination, we found that these enrichments were driven mostly by *GLDN*, a transmembrane collagen associated with ion channels in neural development but also expressed in the placenta (Fig. 6D; Eshed et al. 2005; Maluenda et al. 2016), and *PTPRD*, a transmembrane signaling protein and cell adhesion molecule (Fig. 6D; Veeriah et al. 2009; Zhu et al. 2015). We note that although not contributing to the GO category enrichments, the significantly up-regulated gene *FOSB* is also a regulator of cell matrix adhesion (Fig. 6E; Ohnishi et al. 2008). Collectively, these data indicate that morphokinetically high-quality embryos may be more capable of activating gene expression programs to generate appropriate extracellular matrix and cell–cell adhesions required at the blastocyst stage.

Collectively, we have identified expression trends associated with previously constructed metrics of developmental competence of human preimplantation embryos. Specifically, we found that aneuploidy results in transcriptional changes, indicating an altered metabolic profile; poor morphology is associated with cell cycle changes and disrupted DNA damage response; and poor morphokinetic development is associated with altered transcription of cell adhesion or extracellular matrix genes. These data suggest that lower-quality embryos may lose transcriptional control of a narrowly defined gene expression program and (perhaps as a stress response to genetic or environmental anomalies) aberrantly express genes that disrupt proper development. Future studies are needed to validate these associations and assess their importance in implantation competence.

## Discussion

RNA-seq from small input libraries is revolutionizing our basic understanding of human preimplantation development. However, current studies have not assessed the potential for RNA-seq to inform decision making in clinical embryo selection for IVF (Xue et al. 2013; Yan et al. 2013; Blakeley et al. 2015; Petropoulos et al. 2016; Licciardi et al. 2018). Here, we have asked whether information can be gleaned from high-quality RNA-seq in embryos that would be useful for clinical embryologists, who regularly make decisions based on the sex, karyotype, and perceived quality of the embryo. We have summarized a proof-of-principle data set highlighting the utility of this method for gaining an unprecedented depth of information on the transcriptional control of preimplantation developmental competence. We have shown that RNA-seq can identify preimplantation embryo sex chromosome content, karyotype, and candidate developmental competence gene sets. As such, this method has potential for clinical use.

This initial data set begins to paint a picture of a highly controlled transcriptional program in developmentally competent embryos. Such embryos appear to activate cell–cell adhesion programs that may be responsible for establishing the contacts critical for blastulation and then generating the necessary forces for hatching and implantation. Moreover, we note a trend whereby embryos that do not meet clinical standards for transfer show noisier expression, indicating transcriptional and metabolic stress, reminiscent of the hallmarks of cancer. This is also in line with the concept of human embryos being more viable if they retain a “quiet” metabolic profile (Baumann et al. 2007). It is unclear if the “noisy” transcription is in response to environmental stressors that have already affected the embryo or if an early loss of transcriptional control, perhaps owing to stoichiometric imbalance of protein complexes caused by aneuploidy, has stressed the cells and sped the embryo toward a stress-response program.

A potential complicating factor in the use of any biopsy-based embryo assessment is the possibility that not all cells of the embryo are karyotypically identical, and therefore, the embryo displays mosaicism with respect to karyotype. Estimates of mosaicism rates in cultured human preimplantation embryos vary widely, owing in part to differences in the technology used to assess karyotype and the timing and number of cells biopsied, but the condition is thought to be widespread (van Echten-Arends et al. 2011; McCoy 2017). This makes analysis of concordance between TE biopsy and WE transcriptomes difficult because it can be challenging to separate technical noise from biological signal. We find evidence of mosaicism in, conservatively, a quarter of our embryos, and thus, it is possible that our WE samples contain mixed populations of cells that may limit our ability to accurately assign a karyotype. It follows that this condition also complicates preimplantation genetic screening for aneuploidy, because the cells biopsied and assessed in this technique may not faithfully represent the cell content of the remaining embryo. Indeed, this is one of the main critiques of PGT-A in the field of assisted reproductive technology (Capalbo and Rienzi 2017; Griffin and Ogur 2018).

Indeed, clinical biopsies will always be limited by the uncertainty of the fact that the biopsy samples a subset of one cell type and not a representative fraction of the embryo. Even in the absence of mosaicism, expression concordance between a TE biopsy and the karyotype of the remaining WE will always be limited owing to differing cell type content (mural TE in the biopsy vs. mural and polar TE, epiblast, and primitive endoderm in the remaining WE). The relationship between the information content of a TE biopsy (whether DNA-based karyotype or RNA-seq data) and the remaining embryo remains a topic of active investigation in the reproductive biology community. Our data represent an advancement toward defining this relationship on a transcriptional level. Further studies are necessary to define the mosaicism rates and transcriptional consequences over time in human preimplantation development. Indeed, we feel that within the bounds of what can be ascertained from a TE biopsy owing to sampling limitations, our data are comparable to those obtained by PGT-A. Ultimately, with future studies focusing on transcriptional correlates across multiple predictors of embryo competence, RNA-seq data may identify avenues of noninvasive testing for embryo competence that could forego the need for TE biopsies for preimplantation genetic testing.

Beyond the issue of mosaicism, our analysis is further limited by several other confounding factors that arise because of the extremely limited availability of embryos for research purposes. Although pains were taken to treat each sample uniformly, these embryos were donated by women ages 25–36 (average sample, age 31.1 yr) (see Supplemental File S1), frozen and thawed at different timepoints, and cultured in separate batches to the blastocyst stage. Some embryos reached this developmental timepoint at day 5 of development, whereas some were cultured to day 6 or 7. Moreover, this cohort contains both XX and XY embryos, as well as embryos with varying grades of morphological or morphokinetic quality. Essentially, we expect a high degree of biological variation between any two embryos in this cohort: a reality that reflects the nature of any potential clinical use of RNA-seq for preimplantation screening. More samples (including replicates of each aneuploidy, as well as internal replicates from each WE) are needed to further improve the comprehensiveness of the candidate gene sets and further control for aspects such as sex chromosome content, karyotype, parental age, day of harvest, and variance of morphological or morphokinetic grades.

Our approach to RNA digital karyotyping relies solely on expression values from each sample and does not require SNP genotyping, which can require deep sequencing (Weissbein et al. 2016; Licciardi et al. 2018). As such, this approach is translatable to lighter-sequencing schemes in addition to being computationally accessible.

In conclusion, our study highlights the potential utility of RNA-seq for translational embryo evaluation in IVF. Our data show the ability to generate a digital karyotype from RNA read counts and provide a step forward in our understanding of the molecular underpinnings of human preimplantation embryo competence by assessing expression trends across different known predictive measures of pregnancy. Beyond potential future use in IVF, this information is valuable to inform basic studies of transcriptional events in preimplantation human development, as differential embryo quality (whether morphological, morphokinetic, or digital karyotype) implies altered transcriptional output and thus potentially altered developmental potential.

## Methods

### IRB approval

Harvard University Institutional Review Board (IRB) and Embryonic Stem Cell Research Oversight (ESCRO) committee approval was obtained for both the collection and experimental use of surplus embryos resulting from infertility treatment and donated for research.

### Consent process

All embryos used in this study had previously been donated and stored for research purposes at Harvard University. Couples who had embryos created at various participating facilities and chose to donate surplus embryos for research signed an extensive consent form at the time of their donation. These consent forms were approved by the Harvard University IRB. We did not have access to any identifying personal health information.

### Human embryo thawing

Once received for donation, human embryos created via IVF for the treatment of infertility were stored in liquid nitrogen at −196°C. Briefly, 48 embryos were donated at the zygote stage (two pronuclear [2PN]), 118 embryos were donated at the four- to eight-cell stage (representing a typical day 3 stage of development), and 16 embryos were donated at the blastocyst stage. Embryos were then cultured to the blastocyst stage and graded using the Gardner grading system (Gardner et al. 2013).

Embryo culture dishes were set up using 60-mm culture dishes (BD Falcon) and eight 30-µL drops of continuous single culture medium (Irvine Scientific) overlaid with 10 mL of paraffin oil (Ovoil, Vitrolife). Rinse dishes were also set in a similar format and were equilibrated overnight at 37°C, 5% CO_2_.

Pronuclear, cleavage-stage embryos and blastocysts were thawed using the Quinn's Advantage Thaw Kit (SAGE) (Origio). This kit contains three solutions: 0.5 M sucrose, 0.2 M sucrose, and diluent. For rare embryos that had been frozen using a propanediol-based medium, the appropriate thaw kit from SAGE was used for the thaw (ART-8014).

The straw or vial containing the cryopreserved embryos was placed in a water bath for 2 min at 30°C. The embryos were then expelled from the straw or transferred from the vial using a Pasteur pipette to a clean tissue culture dish on a heated stage. The embryos were located and thawed according to the manufacturer's instructions.

Each embryo was rinsed by passing through culture media and then placed in a single drop in the embryo culture dishes described above. Embryos were cultured in a humidified atmosphere at 5% CO_2_ in air and 37°C.

Study embryos thawed either at the pronuclear or cleavage stage were cultured in a time-lapse incubator with standard trigas settings (embryoscope timelapse incubation system, Vitrolife) using 5% CO_2_, 5% O_2_, and 90% N_2_. Images were obtained every 20 min throughout the course of embryo development, and embryos were removed only for the purpose of embryo biopsy and were replaced in the corresponding well after the biopsy process was complete.

### Embryo evaluation

Pronuclear embryos were evaluated for viability after the thawing process, whereas embryos thawed at the cleavage stage were evaluated 2–4 h after the thawing process. Embryos donated as blastocysts were cultured for a single day to ensure they survived the thaw (and were evaluated for viability within 1 d of the thawing process). Embryos were evaluated using a Nikon Eclipse 80i microscope, and images of each embryo were obtained at 40× using Hamilton Thorne Clinical Laser software. These images were then evaluated by a senior embryologist and a physician for the purposes of embryo grading and assessment of survival.

### Embryo biopsy

Cleavage-stage embryos were biopsied on a heated stage using a laser to create a defect in the zona pellucida sufficient for the removal of a single blastomere. Those embryos were then returned to their respective wells for culture to the blastocyst stage of development. All cleavage-stage embryos were biopsied. Embryos were then reassessed for potential biopsy at the blastocyst stage about 2–3 d later, and five to 10 cells were biopsied at that stage of development. Biopsied samples were then processed in the same way as the embryos, described below. TE biopsies obtained for the purposes of PGT-A were placed in a proprietary DNA buffer, snap frozen on dry ice, and transferred to storage at – 80°C until analysis was completed by Invitae.

### Human embryo collection

Single viable embryos were passed through culture media under mineral oil before an acidic Tyrode's solution (Sigma-Aldrich) wash to dissolve the zona pellucida, somatic cellular debris, and additional sperm. The embryo was then rinsed in clean media drops (HEPES-buffer) and placed in 5 µL of TCL solution containing 1% 2-mercaptoethanol, spun, and snap frozen on dry ice.

Overall, 182 embryos were donated to this study from a databank of donations; 151 recovered from the thaw procedure and were cultured. Of these, 51 developed to blastocyst stage (i.e., 95 senesced before blastocyst stage, and five were unviable as blastocysts). From 51 blast-stage embryos, 42 WE and 20 TE biopsy preps yielded usable libraries. We sequenced these libraries and filtered an additional eight samples owing to potential tetraploid status (from preimplantation genetic screening for aneuploidies or suboptimal sample conditions). This yielded the 35 WEs and 19 TE biopsies depicted in Figure 1. For subsequent analysis, we filtered an additional three biopsy samples which did not pass our final quality-control step (i.e., too few genes expressed, fewer than 5000 genes).

### Library preparation and sequencing

Libraries were prepared according to the Smart-seq2 protocol (Picelli et al. 2014), using a small volume library preparation kit (Nextera), and were assessed for quality and quantified using a Qubit fluorometer and Agilent Bioanalyzer. Samples were sequenced at Harvard's Bauer sequencing core on a HiSeq 2500.

### Analysis

Sequencing reads were trimmed to 50 bp to ensure uniform high quality in the FASTQ scores and then aligned with RSEM version 1.2.29 to hg19 (International Human Genome Sequencing Consortium 2001; Li and Dewey 2011). Because updates to the genome in GRCh38 focused mostly on noncoding regions or regions we otherwise excluded, realignment to GRCh38 is not anticipated to alter the main findings reported here. FastQC and the fastx_toolkit were used in preliminary quality control measures. RSEM-derived counts or TPM tables were used throughout the remainder of the analysis, along with a variety of packages available through Bioconductor in R (R Core Team 2018). All code is available as Supplemental File S2.

We removed spuriously expressed genes (<1 TPM in all samples) and also removed samples expressing fewer than 5000 genes (representing about one-third the maximum transcriptome coverage we observed in other samples) (Supplemental Fig. S1B–D). As a result, three TE biopsy samples were excluded for failing to pass quality control (embryos 7, 13, and 36). Additionally, we removed mitochondrial genes as these represented about one-tenth of the total RNA read counts. The total set of expressed genes for the data set was defined by those which reach at least 1 TPM of expression in one or more samples. This kept the gene list as permissive as possible (we want to identify aberrant expression) while excluding noise. For the transcriptome coverage analysis, we limited this list to 1 TPM in 10 or more samples per sample type (TE or WE). For WE, we identified 13,175 expressed transcripts, whereas TE samples expressed 6086 transcripts. We cannot rule out the possibility that the restricted transcriptome in TE is owing to sample prep bias against smaller input and not a restricted transcriptional program in this specific lineage. However, sample prep bias should be further evidenced by more jackpotting in TE samples, and this appears to not be the case (Supplemental Fig. S1F).

### Sex chromosome content

Embryo sex chromosome content was inferred from either WE or TE samples by first summarizing TPM values for *DDX3Y*, *RPS4Y1*, and *EIF1AY* (three robustly expressed genes on the Y Chromosome) and considering expression >25 TPMs as evidence for presence of a Y in the sample (Petropoulos et al. 2016). Next, we generated a *Z*-score for sequencing depth–normalized read counts across the entire X Chromosome. Briefly, all genes mapping to the X (with the exception of the pseudoautosomal region 1 and 2 genes) were collected and summed, per sample, and normalized by the sequencing depth for that sample. The mean and SD for this normalized read count were calculated across all samples passing quality control per sample type (WE or TE), and these statistics were used to calculate a *Z*-score for each sample. A positive *Z*-score was considered evidence for two copies of the X, whereas a negative *Z*-score provided evidence for a single copy. These results were then compared with PGT-A sex chromosome content calls where available and between WE and TE samples originating from the same embryo where available.

Differential expression analysis by sex chromosome content was conducted in R 3.5.1 using DESeq2 1.2.0.

### RNA digital karyotype

*Z*-Scores for every autosome were calculated as described above. Note that we did not filter stringently for expression (as with Chromosome Y) for either X or the autosomes. A stringent-filtering approach ubiquitous expression operates under the assumption that all embryos are adhering to the same developmental program, but competent versus incompetent embryos may systematically diverge, and time-of-harvest will additionally influence which genes are expressed in a given blastocyst. Rather, our current approach for Chromosome X and the autosomes removes the noisiest genes (those expressed <1 TPM in all samples) and then treats each entire chromosome as a transcriptional unit, taking the least-biased approach to quantifying chromosome-wide expression. A *Z*-score cutoff of ±2 was chosen for preliminary outlier analysis to maximize sensitivity. Note that we do not assume that the distributions are perfectly normal; however, the data values (normalized sums across each chromosome, “normcounts”) follow roughly a bell-shaped curve for each chromosome in which the means and medians are nearly identical but not centered on zero. We reasoned that the *z*-score provides us a reasonable standard measure of deviance from the mean for each chromosome, and the ±2 *Z*-score range provides a permissive first approximation for flagging outlying chromosome scores. If the data followed a perfect normal distribution, the ±2 *Z*-score range would encompass 95% of all observations, which is why we chose this as our “permissive” cutoff. To gain a more stringent view of which chromosomes may reliably be aneuploid in these samples, we generated a bootstrapped *P*-value associated with each *Z*-score. To generate this *P*-value, we shuffled chromosome labels for each gene in our analysis and repeated the *Z*-score calculation 3000 times, keeping track of how often we observed a *Z*-score at least as extreme as the true *Z*-score for each chromosome in each sample.

We used the shuffled distribution of *Z*-scores (Supplemental Fig. S3B) to assign a bootstrapped *P*-value to each chromosome's true *Z*-score. We highlighted three individual WE samples in which both RNA karyotype and PGT-A results agree on chromosome content: a euploid sample, a trisomy 16, and monosomy 4 (Supplemental Fig. S3C). Across each of these embryos, we took a closer look at the distribution of shuffled *Z*-score values to identify how extreme each significantly called *Z*-score is compared with the shuffled majority. We found that, indeed, *Z*-scores that are called significant lie at the extreme left or right of the distribution (depending on whether the *Z*-score indicates a loss or gain of transcriptional activity), and *Z*-scores that are not called significant are situated more centrally in the bulk of the distribution. These data visualize our approach and show how RNA read counts can be used to identify chromosomal abnormalities in preimplantation human embryos at least for a subset of chromosomes.

By using this more stringent approach, we identified 16 incidences of significantly abnormal gene expression from a whole chromosome across 12 embryos (Supplemental Fig. S3D). PGT-A results agree with these relative chromosome expression calls 386 times and disagree only 10 times (Supplemental Fig. S3D). Note that the embryo found to contain a complex chromosomal aneuploidy (involving more than two chromosomes) by PGT-A, E39, had no chromosomes individually called aneuploid using this method. This is because expression from the sample is very noisy across multiple chromosomes (Supplemental Fig. S3A), and so the distribution of shuffled *Z*-scores is broad (Supplemental Fig. S3B). As a result, the signal from any given chromosome never reaches significance. This example illustrates why both the permissive and stringent approaches to calling digital RNA karyotype are necessary. Each of these approaches provides a different piece of the picture, and together, they provide more confidence for understanding which samples show truly aberrant expression.

### Differential expression

Differential expression was performed on RSEM read count data using the DESeq2 (v1.12.4) (Love et al. 2014) package in R 3.3.3 on sample partitions described in the text. Briefly, these were based on (1) RNA-karyotype status (aneuploid vs. euploid), (2) morphological result based on the Gardner grading scale (any AA embryo vs. any CC embryo, regardless of expansion status), or (3) morphokinetic call (embryos meeting clinical criteria vs. those with two or more time-points outside 1 SD of clinical samples). Note removal of mitochondrial genes before or after differential expression analysis makes no difference as DESeq2 ignores extremely abundant transcripts (Rapaport et al. 2013; Conesa et al. 2016). GSA, GO, and TPM visualizations were performed in R 3.3.3 using packages available in Bioconductor.

## Data access

All raw data generated for this study have been submitted to the European Genome-phenome Archive (EGA; https://www.ebi.ac.uk/ega/) under accession number EGAS00001003667.

## Supplementary Material

Supplemental Material
