# Supplementary material for: RNA-seq as a tool for evaluating human embryo competence
Source: Genome Res. 2019 Oct;29(10):1705–18. doi: 10.1101/gr.252981.119 (PMC6771404; doi:10.1101/gr.252981.119)
Supplement: Supplemental Material [file supp_29_10_1705__index.html]

RNA-seq as a tool for evaluating human embryo competence — Supplemental Material 

# RNA-seq as a tool for evaluating human embryo competence

## Supplemental Material

- Supplemental\_Materials\_Table\_of\_Contents.docx
- Supplemental\_File\_1\_embryo\_metadata.txt
- Supplemental\_File\_2\_AllRCode\_Review.docx
- Supplemental\_File\_3\_XX-v-XY\_siggenes.txt
