# Supplementary material for: RNA-seq as a tool for evaluating human embryo competence
Source: Genome Res. 2019 Oct;29(10):1705–18. doi: 10.1101/gr.252981.119 (PMC6771404; doi:10.1101/gr.252981.119)
Supplement: Supplemental Material [file supp_gr.252981.119_Supplemental_Materials_.docx]

**RNA-seq as a tool for diagnosing human embryo competence**

**Supplemental Information**

**Contents**

This file contains the following:

- Figures S1-S4
- Table S1
- Description of Supplemental Files 1-3

**Supplemental Figures**

**Figure S1:** Supplement to overview--quality control measures for embryo RNA-seq. (A) Principle component analysis (PCA) of all samples showing the first two PCs color coded by sample type. Trophectoderm biopsy samples in gray (TE) and whole embryo (WE) samples in black. (B) Number of genes expressed > 1 transcript per million (TPM, y-axis) for each embryo in our analysis (x-axis). Black indicates WE sample and gray indicates TE sample. (C) Boxplot depicting average number of genes per sample-type. Grey indicates TE and black indicates WE. (D) Percent coverage of the expressed transcriptome for TE (gray) or WE (black) samples. See methods for details on how expressed transcriptome is defined. (E) Log(TPM) expression distribution for each sample in this analysis. WE samples in black and TE samples in gray. (F) Relationship between max expression (in TPM, y-axis) and sample transcriptome coverage (x-axis) for TE samples (gray) or WE samples (black). (G) PCA of all samples showing the first two PCs color coded by read counts.

**Figure S2:** Supplement to RNA-based embryo sex chromosome content. (A) Expression in TPMs of classical genotyping genes *XIST* and *SRY* in each trophectoderm or whole embryo sample. Embryos along the x-axis. (B) Histograms of expressed Y-specific gene TPM sums in trophectoderm biopsy or whole embryo samples. TPM sums for *RPS4Y1, DDX3Y, and EIF1AY* on the X-axis, number of occurrences on the Y axis. Bottom panel shows a zoom-in of 1-100 (summed TPM). (C) Number of significantly differentially expressed genes (DEGs) between XX and XY embryos by chromosome. Chromosome indicated on X-axis and number of DEGs on Y-axis. (D) Row-normalized expression of log_2_-transformed TPM values for 88 DEGs with Log_2_ Fold Change >2 in XX-vs-XY whole embryo comparison. Column dendrogram separates XY from XX embryos.

**Figure S3:** Supplement to RNA-based digital karyotype. (A) Z-score profiles for each embryo in this analysis. Chromosomes on the X-axis, Z-score on the Y-axis. Whole embryo samples in black, trophectoderm biopsy samples in gray. (B) Shuffled Z-score distributions for every embryo in this analysis. Embryo on X-axis, Z-score on Y-axis. Whole embryo in black, trophectoderm biopsy in gray. (C) Z-score profile for Embryo 28 (euploid), Embryo 29 (trisomic for chr16 according to PGT-A), and Embryo 35 (monosomic for chr4 by PGT-A). Z-score is indicated on the y-axis and chromosome is indicated on the x-axis. Red dot indicates outlying z-score. (D) Karyotype overview (as in Figure 3A) for all whole embryos in this analysis using the stringent cutoff (see methods for details). Gray indicates normal chromosome content, red indicates gain of chromosome, and blue indicates loss of chromosome. Chromosome on the Y-axis, and embryo on the X-axis. Red labeling indicates results of DNA-based PGT-A where applicable.

**Figure S4:** Expression of mitochondrial genes in euploid and aneuploid whole embryos and trophectoderm biopsies. Asterisks (**, red) indicates significant difference by t-test. TPMs on the Y-axis.

**Table S1:** Summary of evidence for mosaicism in whole embryo samples.

| Embryo | Evidence of mosaicism |
| --- | --- |
| E1 | PGT-A indicates Y, but RNA-seq contains no Y reads |
| E5 | Chr Y reads under-represented in biopsy |
| E8 | Discordant outlier calls between TE-WE and Chr Y reads under-represented in biopsy |
| E9 | Chr Y reads under-represented in biopsy |
| E35 | Discordant outlier calls between TE-WE and Chr Y reads under-represented in biopsy |

**Description of additional supplemental files**

**Supplemental File 1 – Embryo Metadata –** This file includes all available metadata for each sample used in this study.

**Supplemental File 2 – All R code used in this analysis –** This file contains a concatenation of all R Markdown files used to generate the figures and data reported in this paper.

**Supplemental File 3 – XX-vs-XY differentially expressed genes –** This file contains the results of the XX-v-XY differential expression analysis, including significantly differentially expressed genes and their chromosome assignments.
