# Supplementary material for: RNA-seq as a tool for evaluating human embryo competence
Source: Genome Res. 2019 Oct;29(10):1705–18. doi: 10.1101/gr.252981.119 (PMC6771404; doi:10.1101/gr.252981.119)
Supplement: Supplemental Material [file supp_gr.252981.119_Supplemental_File_2_AllRCode_Review.docx]

---

title: "Figure1_Embryo"

output:

html_document: default

html_notebook: default

---

Code to generate figure 1, establish QC thresholds

QC: 1TPM minimum and ~1/3 max genes (~5k)

# Setup and read in data

```{r knitropts}

knitr::opts_chunk$set(echo=FALSE, message=FALSE, warning=FALSE,fig.path = "/fig1overview_images/", dev=c('png', 'pdf'))

```

```{r setup}

library(DESeq2)

library(tximport)

library(readr)

library(stringr)

library(ggplot2)

library(reshape2)

library(plyr)

library(dplyr)

#rsem_count_dir<-"/Volumes/valor2/users/agroff/seq/humanEmbryo/quants/rsem/50bptrim"

rsem_count_dir<-"~/Dropbox/Rinnlab/manuscripts/EmbryoPaper2018/Review1/rsem_files/"

#make transcript to gene conversion file using dat1 from above

files <- list.files(rsem_count_dir,pattern="*.genes.results",full.names=TRUE)

filenames<-list.files(rsem_count_dir,pattern="*.genes.results")

filenames<-gsub(".genes.results","",filenames)

names(files) <- filenames

metadata<-read.table("~/Dropbox/Rinnlab/manuscripts/EmbryoPaper2018/Review1/FigsSup/032018_WE_ploidy_metadata.txt",header=TRUE,stringsAsFactors = FALSE)

txn_chr_info<-read.table("~/Dropbox/PageLab/Projects/SexDiffs/supporting/txn_chr_info.tab",header=TRUE)

chr_sizes<-read.table("~/Dropbox/PageLab/Projects/SexDiffs/supporting/chrom_sizes.tab",header=TRUE)

tx2gene<-read.table("/Volumes/solexa_page/agroff/seq/humanEmbryo/analysis_old/analysis/tx2gene.tab",header=TRUE)

annot_short<-read.table("~/Dropbox/Rinnlab/manuscripts/EmbryoPaper2018/Review1/FigsSup/embryo_transcript_annotation_gencodev19_hg19.tab")

potentially_tetraploid<-c("NRAG41","NRAG78","NRAG82","NRAG90","NRAG93","NRAG95","NRAG66")

tet<-metadata[metadata$ProcessingID%in%potentially_tetraploid,]

metadata<-metadata[!metadata$ProcessingID%in%potentially_tetraploid,]

```

```{r definesamples_and_load, include=FALSE}

metadata<-metadata[which(metadata$Sampletype!="day3"),]

diff_files<-files[which(names(files)%in%metadata$ProcessingID)]

diff_metadata<-metadata

txi <- tximport(diff_files, type="rsem", tx2gene=tx2gene)#, reader=read_tsv)

rownames(diff_metadata)<-diff_metadata$ProcessingID

diff_metadata<-diff_metadata[order(diff_metadata$ProcessingID),]

#add 1 to all lengths in txi

#https://support.bioconductor.org/p/92763/

txi$length<-txi$length+1

ddsTxi <- DESeqDataSetFromTximport(txi, colData=diff_metadata,design=~Sampletype)

counts_data<-ddsTxi@assays[[1]]

count_sums<-as.data.frame(colSums(counts_data))

count_sums$ProcessingID<-row.names(count_sums)

names(count_sums)[1]<-"counts"

diff_metadata_counts<-merge(diff_metadata,count_sums)

diff_metadata<-diff_metadata_counts

ddsTxi <- DESeqDataSetFromTximport(txi, colData=diff_metadata,design=~Sampletype)

```

# Pre-Filtering PCA

PCA before anything

```{r PCA}

dds<-ddsTxi

nrow(dds)

dds <- dds[ rowSums(counts(dds)) > 1, ]

nrow(dds)

vsd<-varianceStabilizingTransformation(dds)

#par(mfrow=c(1, 2))

dds <- estimateSizeFactors(dds)

#pdf("unfiltered_PCA_sampletype.pdf")

plotPCA(vsd, intgroup = c("Sampletype"))+ggtitle("PCA, Sampletype")+scale_color_manual(values=c("day3"="light blue","trophectodermbiopsy"="grey","wholeembryo"="black"))+theme_bw()

#dev.off()

#pdf("unfilitered_PCA_counts.pdf")

plotPCA(vsd, intgroup = c("counts"))+ggtitle("PCA, counts")+theme_bw()+scale_colour_gradient("low"="white","high"="black")

#dev.off()

```

# Quality Filter

```{r TPM_numb_geneexp}

tpm_files<-lapply(diff_files,read.table,header=TRUE)

expression_info<-lapply(tpm_files,function(x){

newx<-x[,c("gene_id","TPM")]

newx

})

exp_info2<-lapply(seq_along(expression_info),function(x){

new<-expression_info[[x]]

new$samplename<-names(expression_info)[x]

as.data.frame(new)

})

expression_info<-do.call("rbind",exp_info2)

expression_info$genename<-str_split_fixed(expression_info$gene_id,"_",2)[,2]

expression_annot<-merge(expression_info,metadata,by.x="samplename",by.y="ProcessingID")

library(data.table)

expression_annot_dt<-data.table(expression_annot)

expression_annot_dt$infocondition<-paste(expression_annot_dt$gene_id,expression_annot_dt$devpotential,sep="_")

expressed_genes_samples<-expression_annot_dt[expression_annot_dt$TPM>1,]

expressed_genes_samples<-expressed_genes_samples[,c("samplename","TPM","genename","ReadableEmbryoID","Sampletype")]

genes_per_sample<-ddply(expressed_genes_samples,~samplename,nrow)

names(genes_per_sample)<-c("samplename","genescount")

numgenes_per_sample_annot<-merge(genes_per_sample,diff_metadata,by.x="samplename",by.y="ProcessingID")

numgenes_per_sample_annot<-numgenes_per_sample_annot[order(numgenes_per_sample_annot$EmbryoNumber),]

numgenes_per_sample_annot$PaperID<-factor(numgenes_per_sample_annot$PaperID,levels=as.character(numgenes_per_sample_annot$PaperID))

ggplot(numgenes_per_sample_annot,aes(PaperID,genescount,colour=Sampletype))+geom_point()+theme_bw()+scale_color_manual(values=c("trophectodermbiopsy"="grey","wholeembryo"="black"))+theme(axis.text.x = element_text(angle = 90, hjust = 1))

#dev.off()

XistAndSry<-expression_annot[grep("XIST|SRY",expression_annot$genename),]

XistAndSry<-XistAndSry[order(XistAndSry$EmbryoNumber),]

XistAndSry$PaperID<-factor(XistAndSry$PaperID,levels=XistAndSry$PaperID)

#pdf("Xist_and_Sry.pdf")

ggplot(XistAndSry,aes(PaperID,genename,fill=TPM))+geom_tile()+theme_bw()+coord_equal()+scale_fill_gradient2(low="white",high="black")+facet_wrap(~Sampletype)

#dev.off()

#pdf("boxplot_numgenes_sampletype_prefiltering.pdf")

ggplot(numgenes_per_sample_annot,aes(Sampletype,genescount,fill=Sampletype))+geom_boxplot()+theme_bw()+scale_fill_manual(values=c("trophectodermbiopsy"="grey","wholeembryo"="black"))

#dev.off()

```

# Define QC thresh

QC filtering mito reads and par1

```{r QC_mitoandpar1}

#remove mitochondrial reads here

counttable<-as.data.frame(counts(dds))

counttable$geneinfo<-row.names(counttable)

counttable$txname<-str_split_fixed(counttable$geneinfo,"_",2)[,1]

counttable$genename<-str_split_fixed(counttable$geneinfo,"_",2)[,2]

counttable_chr<-merge(counttable,txn_chr_info,by.x="txname",by.y="TXNAME")

mito<-counttable_chr[which(counttable_chr$chr=="chrM"),]

counttable<-counttable_chr[which(counttable_chr$chr!="chrM"),]

genomic_genes<-counttable$geneinfo

dds<-dds[row.names(dds)%in%genomic_genes,]

nrow(dds)

dds<-dds[row.names(dds)%in%genomic_genes,]

nrow(dds)

#remove PAR1 genes

par1<-c("PLCXD1","GTPBP6","PPP2R3B","SHOX","CRLF2","CSF2RA","IL3RA","SLC25A6","ASMTL","P2RY8","CXYorf3","ASMT","DHRSXY","ZBED1","CD99","XG")

par2<-c("VAMP7","IL9R","WASH6P")

#PRY3, SYBL1, IL9R and CXYorf1 --> SYBL1 == VAMP7; CXYorf1 is WASH6P; cant find PRY3

par1_genes<-counttable[which(counttable$genename %in% c(par1,par2)),"geneinfo"]

dds<-dds[!(row.names(dds)%in%par1_genes),]

```

What is the mito-weight per sample?

```{r mito}

mito.melt<-melt(mito)

mito.melt.annot<-merge(mito.melt,diff_metadata,by.x="variable",by.y="ProcessingID")

ggplot(mito.melt.annot,aes(RNAPloidy,log(value)))+geom_boxplot(notch=TRUE)+geom_jitter()+facet_wrap(~Sampletype)+theme_bw()

mito.melt.sum<-ddply(mito.melt[,c("variable","value")],.(variable),summarize,sampleSums=sum(value))

mito.melt.sum.annot<-merge(mito.melt.sum,diff_metadata,by.x="variable",by.y="ProcessingID")

ggplot(mito.melt.sum.annot,aes(RNAPloidy,log(sampleSums)))+geom_boxplot(notch=TRUE)+geom_jitter()+facet_wrap(~Sampletype)+theme_bw()

mito.melt.annot<-merge(mito.melt,diff_metadata,by.x="variable",by.y="ProcessingID")

ggplot(mito.melt.annot,aes(PaperID,value,color=Sampletype))+geom_boxplot()#geom_jitter()

ggplot(mito.melt.annot,aes(Geno,log(value)))+geom_boxplot(notch=TRUE)+facet_wrap(~Sampletype)

TPMs<-txi$abundance

mitogenesinfo<-tx2gene[tx2gene$TXNAME %in% mito$txname,]

mitogenesinfo$info<-paste(mitogenesinfo$TXNAME,mitogenesinfo$GENEID,sep="_")

mito_TPMs<-TPMs[mitogenesinfo$info,]

```

For TPM version, using TPM 1 as thresh. save samples expressing 1/3+ max # genes

```{r defineQC}

#reshape expression_annot to wide

expression_annot_towide<-expression_annot[,c("samplename","gene_id","TPM")]

expression_annot_towide$TPM<-as.numeric(expression_annot_towide$TPM)

TPM_exp_wide<-reshape(expression_annot_towide,idvar="gene_id",timevar="samplename",direction="wide")

names(TPM_exp_wide)<-gsub("TPM[.]","",names(TPM_exp_wide))

binary_TPM<-TPM_exp_wide

row.names(binary_TPM)<-binary_TPM$gene_id

binary_TPM$gene_id<-NULL

binary_TPM<-as.matrix(binary_TPM)

binary_TPM[binary_TPM<1]<-0

binary_TPM[binary_TPM>=1]<-1

binary_TPM<-as.data.frame(binary_TPM)

binary_TPM$sums<-rowSums(binary_TPM)

binary_TPM<-binary_TPM[which(binary_TPM$sums>1),] #genes exp in at least 1 sample at least 1 TPM

expressed_genes<-row.names(binary_TPM) #print this list to read into other files

#save this file of genenames, read in at begining of each subsequent analysis. This is the list of genes that pass QC thresholds!

#write.table(expressed_genes, file="/Volumes/valor2/users/agroff/seq/humanEmbryo/analysis/Feb2018_expressed_genes.tab",col.names=TRUE,quote=FALSE,sep="\t")#old

#expressed_genes<-read.table("/Volumes/valor2/users/agroff/seq/humanEmbryo/analysis/Feb2018_expressed_genes.tab",header=TRUE)#old

#write.table(expressed_genes, file="/Volumes/valor2/users/agroff/seq/humanEmbryo/analysis/March2018_expressed_genes.tab",col.names=TRUE,quote=FALSE,sep="\t")#old

#expressed_genes<-read.table("/Volumes/valor2/users/agroff/seq/humanEmbryo/analysis/March2018_expressed_genes.tab",header=TRUE)#old

expressed_genes<-expressed_genes$x

sumgenesExp1TPM<-colSums(binary_TPM[,1:54])

summary(sumgenesExp1TPM)

# Min. 1st Qu. Median Mean 3rd Qu. Max.

# 3984 8679 11440 10390 12280 14010

samples_keep<-as.data.frame(sumgenesExp1TPM[which(sumgenesExp1TPM>5000)])

samples_keep$ProcessingID<-rownames(samples_keep)

names(samples_keep)<-c("genecount","ProcessingID")

#save this file of samples to keep, read in at begining of each subsequent analysis. This is the list of samples that pass QC thresholds!

#write.table(samples_keep, file="/Volumes/valor2/users/agroff/seq/humanEmbryo/analysis/March2018_samples_passing_QC.tab",col.names=TRUE,quote=FALSE,sep="\t")

samples_keep_data<-diff_metadata[which(diff_metadata$ProcessingID %in% names(samples_keep)),]

samples_keep<-as.data.frame((samples_keep))

names(samples_keep)<-c("ExpGenes")

samples_keep$ProcessingID<-row.names(samples_keep)

samples_keep_data<-merge(samples_keep_data,samples_keep,by.x="ProcessingID",by.y="ProcessingID")

#QCfiltering starts here!

dds<-ddsTxi #start fresh here!

nrow(dds)

dds <- dds[ rowSums(counts(dds)) > 1, ] #get rid of super noisy expression

nrow(dds)

dim(dds)

dds<-dds[,(colnames(dds)%in%samples_keep$ProcessingID)] #get rid of low expressing samples

dim(dds)

nrow(dds)

dds<-dds[row.names(dds)%in%genomic_genes,] #get rid of mitochondial exp

nrow(dds)

#remove PAR1 genes

par1<-c("PLCXD1","GTPBP6","PPP2R3B","SHOX","CRLF2","CSF2RA","IL3RA","SLC25A6","ASMTL","P2RY8","CXYorf3","ASMT","DHRSXY","ZBED1","CD99","XG")

par1_genes<-counttable[which(counttable$genename %in% par1),"geneinfo"]

dds<-dds[!(row.names(dds)%in%par1_genes),]

#keep only remaining expressed genes (>=10TPM in at least 1 sample)

dds<-dds[row.names(dds)%in%expressed_genes,]

vsd<-varianceStabilizingTransformation(dds)

par(mfrow=c(1, 2))

dds <- estimateSizeFactors(dds)

#> dim(dds)

#[1] 21581 51

pdf("QC_filtered_PCA_sampletype.pdf")

plotPCA(vsd, intgroup = c("Sampletype"))+ggtitle("PCA, Sampletype")+scale_color_manual(values=c("day3"="light blue","trophectodermbiopsy"="grey","wholeembryo"="black"))+theme_bw()

dev.off()

```

TPM distributions for all samples used (post QC, supplement)

```{r TPMdistributions, include=FALSE}

#filter for expressed genes and expressed samples

expression_annot_dt_filtered<-expression_annot_dt[expression_annot_dt$gene_id %in% row.names(dds),]

expression_annot_dt_filtered<-expression_annot_dt_filtered[expression_annot_dt_filtered$samplename %in% colnames(dds),]

pdf("QC_filtered_embryo_sample_distributions_bytype_mitoremoved.pdf")

ggplot(expression_annot_dt_filtered,aes(log(TPM),fill=Sampletype))+geom_density()+theme_bw()+facet_wrap(~samplename)+scale_fill_manual(values=c("trophectodermbiopsy"="grey","wholeembryo"="black"))

dev.off()

```

Transcriptome coverage

binary = which genes expressed > 1 tpm..

keeping genes expressed in 10 or more whole embryos --> ~13175 genes

```{r transcriptomecapture}

#keep whole embryos only

transcriptome<-binary_TPM[,colnames(binary_TPM) %in% metadata[which(metadata$Sampletype=="wholeembryo"),"ProcessingID"]]

#keep only genes expressed in 10 or more embryos--call full transcriptome?

transcriptomesums<-rowSums(transcriptome)

transcriptome<-transcriptome[which(transcriptomesums>10),] #for now consider this geneset essentially the transcriptome max? could probably be refined...

max_genes_WE<-dim(transcriptome)[1]

#do we care if theyre the same genes or just # genes? for now lets say same genes...

# grab all samples

txn_capture_calc<-binary_TPM[which(row.names(binary_TPM) %in% row.names(transcriptome)),]

txn_capture_calc$sums<-NULL

num_txome_genes_expressed_1tpm<-as.data.frame(colSums(txn_capture_calc))

names(num_txome_genes_expressed_1tpm)<-"genes_exp"

num_txome_genes_expressed_1tpm$percent_txome<-num_txome_genes_expressed_1tpm$genes_exp/max_genes_WE

num_txome_genes_expressed_1tpm$ProcessingID<-row.names(num_txome_genes_expressed_1tpm)

########MAKE SEP TEBX TXOME TO CALC RECOVERY #########

transcriptome_TE<-binary_TPM[,colnames(binary_TPM) %in% metadata[which(metadata$Sampletype=="trophectodermbiopsy"),"ProcessingID"]]

#keep only genes expressed in 10 or more embryos--call full transcriptome?

transcriptomeTEsums<-rowSums(transcriptome_TE)

transcriptome_TE<-transcriptome_TE[which(transcriptomeTEsums>10),] #for now consider this geneset essentially the transcriptome max? could probably be refined...

max_genes_TE<-dim(transcriptome_TE)[1]

#do we care if theyre the same genes or just # genes? for now lets say same genes...

# grab all samples

txn_capture_TE_calc<-binary_TPM[which(row.names(binary_TPM) %in% row.names(transcriptome_TE)),]

txn_capture_TE_calc$sums<-NULL

num_txome_TE_genes_expressed_1tpm<-as.data.frame(colSums(txn_capture_TE_calc))

names(num_txome_TE_genes_expressed_1tpm)<-"genes_exp"

num_txome_TE_genes_expressed_1tpm$percent_txome<-num_txome_TE_genes_expressed_1tpm$genes_exp/max_genes_TE

num_txome_TE_genes_expressed_1tpm$ProcessingID<-row.names(num_txome_TE_genes_expressed_1tpm)

txome_capture_TE_annot<-merge(num_txome_TE_genes_expressed_1tpm,metadata)

txome_capture_TE_annot<-txome_capture_TE_annot[grep("trophectodermbiopsy",txome_capture_TE_annot$Sampletype),]

txome_capture_WE_annot<-merge(num_txome_genes_expressed_1tpm,metadata)

txome_capture_WE_annot<-txome_capture_WE_annot[grep("wholeembryo",txome_capture_WE_annot$Sampletype),]

write.table(txome_capture_WE_annot,file="WE_transcriptome_capture.txt",quote=FALSE,sep="\t",row.names=FALSE)

write.table(txome_capture_TE_annot,file="TE_transcriptome_capture.txt",quote=FALSE,sep="\t",row.names=FALSE)

txome_capture_all<-rbind(txome_capture_WE_annot,txome_capture_TE_annot)

write.table(txome_capture_all,file="transcriptome_capture_bysampletype.txt",quote=FALSE,sep="\t",row.names=FALSE)

pdf("transcriptome_capture_sampletype_jitter.pdf")

ggplot(txome_capture_all,aes(Sampletype,percent_txome,colour=Sampletype))+geom_jitter()+theme_bw()+scale_colour_manual(values=c("trophectodermbiopsy"="gray","wholeembryo"="black"))

dev.off()

pdf("transcriptome_capture_sampletype_violin.pdf")

ggplot(txome_capture_all,aes(Sampletype,percent_txome,colour=Sampletype))+geom_violin()+theme_bw()+scale_colour_manual(values=c("trophectodermbiopsy"="gray","wholeembryo"="black"))

dev.off()

#from Fig2-- Are E35,E8 TEBx on lower end of TEBx txome coverage?

ggplot(txome_capture_annot,aes(Sampletype,percent_txome,colour=Sampletype,label=PaperID))+geom_jitter()+theme_bw()+scale_colour_manual(values=c("trophectodermbiopsy"="gray","wholeembryo"="black"))+geom_text(data=subset(txome_capture_annot,PaperID=="E35"|PaperID=="E8"))

#### REDO W ALL EXP GENES (most permissive for exp) - 1TPM in 1Sample --> transcriptome of like 21k ish

transcriptome_all<-binary_TPM[,which(colnames(binary_TPM) %in% colnames(dds))] #keep genes expressed in 1 or more embryos (already there)

max_genes_all<-dim(transcriptome_all)[1]

num_txome_genes_expressed_1tpm_all<-as.data.frame(colSums(transcriptome_all))

names(num_txome_genes_expressed_1tpm_all)<-"genes_exp"

num_txome_genes_expressed_1tpm_all$percent_txome<-num_txome_genes_expressed_1tpm_all$genes_exp/max_genes_all

num_txome_genes_expressed_1tpm_all$ProcessingID<-row.names(num_txome_genes_expressed_1tpm_all)

txome_capture_allexpgenes_annot<-merge(num_txome_genes_expressed_1tpm_all,metadata)

write.table(txome_capture_allexpgenes_annot,file="transcriptome_capture_allexpgenes_bysampletype.txt",quote=FALSE,sep="\t",row.names=FALSE)

```

Evidence of jackpotting in samples? (esp in those of low txome coverage?)

```{r evidenceOfJackpot}

summary_of_samples<-ddply(expression_annot_dt_filtered,.(samplename),summarise,TPMMax=max(TPM),TPMMed=median(TPM),TPMmean=mean(TPM))

#ddply(expression_annot_dt_filtered, .(samplename), function(x) quantile(x$TPM))

TPM_quantiles_by_sample<-ddply(expression_annot_dt_filtered, .(samplename), function(x) quantile(x$TPM,probs=c(0.25,0.50,0.75,0.9,0.99,1)))

summary_of_samples.melt<-melt(summary_of_samples)

TPM_quantiles_by_sample.melt<-melt(TPM_quantiles_by_sample)

summary_data_tpms<-rbind(summary_of_samples.melt,TPM_quantiles_by_sample.melt)

ggplot(summary_data_tpms,aes(variable,log(value)))+geom_violin()+theme_bw()+theme(axis.text.x = element_text(angle = 90, hjust = 1))

top_subset<-summary_data_tpms[grep("90%|99%|100%",summary_data_tpms$variable),]

ggplot(top_subset,aes(variable,value))+geom_violin()+theme_bw()+theme(axis.text.x = element_text(angle = 90, hjust = 1))

top_subset_annot<-merge(top_subset,metadata,by.x="samplename",by.y="ProcessingID")

ggplot(subset(top_subset_annot,variable=="100%"),aes(variable,value,color=Sampletype))+geom_jitter()+theme_bw()+theme(axis.text.x = element_text(angle = 90, hjust = 1))+scale_colour_manual(values=c("trophectodermbiopsy"="gray","wholeembryo"="black"))

top_subset_annot<-merge(top_subset,txome_capture_allexpgenes_annot,by.x="samplename",by.y="ProcessingID")

pdf("max_TPMval_vs_allgenesPercentTxome.pdf")

ggplot(subset(top_subset_annot,variable=="100%"),aes(percent_txome,value,color=Sampletype))+geom_jitter()+theme_bw()+theme(axis.text.x = element_text(angle = 90, hjust = 1))+scale_colour_manual(values=c("trophectodermbiopsy"="gray","wholeembryo"="black"))

dev.off()

pdf("max_TPMval_jitter.pdf")

ggplot(subset(top_subset_annot,variable=="100%"),aes(variable,value,color=Sampletype))+geom_jitter()+theme_bw()+theme(axis.text.x = element_text(angle = 90, hjust = 1))+scale_colour_manual(values=c("trophectodermbiopsy"="gray","wholeembryo"="black"))

dev.off()

pdf("99percentile_TPMval_jitter.pdf")

ggplot(subset(top_subset_annot,variable=="99%"),aes(variable,value,color=Sampletype))+geom_jitter()+theme_bw()+theme(axis.text.x = element_text(angle = 90, hjust = 1))+scale_colour_manual(values=c("trophectodermbiopsy"="gray","wholeembryo"="black"))

dev.off()

pdf("90percentile_TPMval_jitter.pdf")

ggplot(subset(top_subset_annot,variable=="90%"),aes(variable,value,color=Sampletype))+geom_jitter()+theme_bw()+theme(axis.text.x = element_text(angle = 90, hjust = 1))+scale_colour_manual(values=c("trophectodermbiopsy"="gray","wholeembryo"="black"))

dev.off()

#num genes w exp over 500TPM per sample:

highexp<-expression_annot_dt_filtered[which(expression_annot_dt_filtered$TPM>700),] #99percentile median seems to be around 700)

highexp_bysample<-ddply(highexp,.(samplename),nrow)

```

Each sample has maybe 200 genes w exp > 700TPM (100-150 w exp > 1000, and just a few w a handfull > 5000)

Filtered gene counts figures (TPM >1)

```{r tpm_filterExp}

expressed_genes_samples<-expression_annot_dt_filtered[expression_annot_dt_filtered$TPM>1,]

expressed_genes_samples<-expressed_genes_samples[,c("samplename","TPM","genename","ReadableEmbryoID","Sampletype")]

genes_per_sample<-ddply(expressed_genes_samples,~samplename,nrow)

names(genes_per_sample)<-c("samplename","genescount")

numgenes_per_sample_annot<-merge(genes_per_sample,diff_metadata,by.x="samplename",by.y="ProcessingID")

numgenes_per_sample_annot<-numgenes_per_sample_annot[order(numgenes_per_sample_annot$ReadibleEmbryoNumber),]

numgenes_per_sample_annot$ReadableEmbryoID<-factor(numgenes_per_sample_annot$ReadableEmbryoID,levels=as.character(numgenes_per_sample_annot$ReadableEmbryoID))

pdf("numgenes_per_sample_dots_filtered.pdf")

ggplot(numgenes_per_sample_annot,aes(ReadableEmbryoID,genescount,colour=Sampletype))+geom_point()+theme_bw()+scale_color_manual(values=c("day3"="light blue","trophectodermbiopsy"="grey","wholeembryo"="black"))+theme(axis.text.x = element_text(angle = 90, hjust = 1))

dev.off()

pdf("boxplot_numgenes_sampletype_filtered.pdf")

ggplot(numgenes_per_sample_annot,aes(Sampletype,genescount,fill=Sampletype))+geom_boxplot()+theme_bw()+scale_fill_manual(values=c("day3"="light blue","trophectodermbiopsy"="grey","wholeembryo"="black"))

dev.off()

```

Table for Fig 1

MAY need to redo, removing filtered out samples and re-numbering!! (2/5/18 have not done this yet)

```{r embryo_summary_table}

table_data<-read.table("/Users/abigailgroff/Google Drive/Embryo/Figures/code/current_working/Fig1_Table_New_March2018.tab",header=TRUE,stringsAsFactors = FALSE)

table_data$EmbryoNumber<-as.numeric(gsub("E","",table_data$PaperID))

table_data<-table_data[order(table_data$EmbryoNumber),]

table_data$PaperID<-factor(table_data$PaperID,levels=table_data$PaperID[order(table_data$EmbryoNumber)])

table_data$Notes<-NULL

newmetadata<-merge(metadata,table_data,by.x="ReadableEmbryoID",by.y="OldReadableEmbryoID")

#write.table(newmetadata,file="/Volumes/valor2/users/agroff/seq/humanEmbryo/analysis/March2018_paperID_updated_metadata.txt",quote=FALSE,row.names = FALSE,sep="\t")

table_data$OldReadableEmbryoID<-NULL

table_data.melt<-melt(table_data,id.vars=c("PaperID","EmbryoNumber"))

#ggplot(table_data.melt,aes(variable,PaperID,fill=value))+geom_tile(colour="black")+theme_bw()+scale_fill_manual(values=c("yes"="black","no"="white"))+coord_equal()

pdf("sample_summary_table.pdf")

ggplot(table_data.melt,aes(PaperID,variable,fill=value))+geom_tile(colour="black")+theme_bw()+scale_fill_manual(values=c("YES"="black","NO"="white"))+coord_equal()+theme(axis.text.x = element_text(angle = 90, hjust = 1))

dev.off()

```

---

title: "R Notebook"

output:

html_document: default

html_notebook: default

---

Embryo Sexing - Figure 2

PAR1:

PLCXD1,GTPBP6,PPP2R3B,SHOX,CRLF2,CSF2RA,IL3RA,SLC25A6,ASMTL,P2RY8,CXYorf3,ASMT,DHRSXY,ZBED1,CD99,XG

# Setup

```{r setup}

library(DESeq2)

library(tximport)

library(readr)

library(stringr)

library(ggplot2)

library(reshape2)

library(plyr)

library(dplyr)

rsem_count_dir<-"~/Dropbox/Rinnlab/manuscripts/EmbryoPaper2018/Review1/support_for_code/rsem_files/"

#make transcript to gene conversion file using dat1 from above

files <- list.files(rsem_count_dir,pattern="*.genes.results",full.names=TRUE)

filenames<-list.files(rsem_count_dir,pattern="*.genes.results")

filenames<-gsub(".genes.results","",filenames)

names(files) <- filenames

metadata<-read.table("~/Dropbox/Rinnlab/manuscripts/EmbryoPaper2018/Review1/FigsSup/metadata_021219.txt",header=TRUE,stringsAsFactors = FALSE)

txn_chr_info<-read.table("~/Dropbox/PageLab/Projects/SexDiffs/supporting/txn_chr_info.tab",header=TRUE)

chr_sizes<-read.table("~/Dropbox/PageLab/Projects/SexDiffs/supporting/chrom_sizes.tab",header=TRUE)

tx2gene<-read.table("/Volumes/solexa_page/agroff/seq/humanEmbryo/analysis_old/analysis/tx2gene.tab",header=TRUE)

tx2gene$info<-paste(tx2gene$TXNAME,tx2gene$GENEID,sep="_")

annot_short<-read.table("~/Dropbox/Rinnlab/manuscripts/EmbryoPaper2018/Review1/FigsSup/embryo_transcript_annotation_gencodev19_hg19.tab")

potentially_tetraploid<-c("NRAG41","NRAG78","NRAG82","NRAG90","NRAG93","NRAG95","NRAG66")

metadata<-metadata[!metadata$ProcessingID%in%potentially_tetraploid,]

```

# Define and load samples

```{r definesamples_and_load, include=FALSE}

diff_files<-files[which(names(files)%in%metadata$ProcessingID)]

diff_metadata<-metadata

txi <- tximport(diff_files, type="rsem", tx2gene=tx2gene)

rownames(diff_metadata)<-diff_metadata$ProcessingID

diff_metadata<-diff_metadata[order(diff_metadata$ProcessingID),]

#add 1 to all lengths in txi

#https://support.bioconductor.org/p/92763/

txi$length<-txi$length+1

ddsTxi <- DESeqDataSetFromTximport(txi, colData=diff_metadata,design=~Sampletype)

```

# Quality Filter

Remove genes that are not expressed in any sample

- can add binary screening so must be expressed >1 in any one sample

```{r quality_filter}

#expressed_genes<-read.table("/Volumes/valor2/users/agroff/seq/humanEmbryo/analysis/March2018_expressed_genes.tab",header=TRUE)

samples_keep<-read.table("~/Dropbox/Rinnlab/manuscripts/EmbryoPaper2018/Review1/support_for_code/rsem_files/March2018_samples_passing_QC.tab",header=TRUE)

#quality filtering

#remove genes with no counts

dds<-ddsTxi

nrow(dds)

dds <- dds[ rowSums(counts(dds)) > 1, ] #get rid of super noisy expression

nrow(dds)

dim(dds)

dds<-dds[,(colnames(dds)%in%samples_keep$ProcessingID)] #get rid of low expressing samples

dim(dds)

#how many X and Y genes kept?

txn_chr_info$info<-paste(txn_chr_info$TXNAME,txn_chr_info$GENEID,sep="_")

#1TMP, 1 sample -->

xygenes_1_1<-txn_chr_info[expressed_genes,]

xygenes_1_1<-xygenes_1_1[grep("X|Y",xygenes_1_1$chr),]

xygenes_1_1.sum<-ddply(xygenes_1_1,.(chr),nrow)

#1 chrX 902

#2 chrY 87

#TPMs<-txi$abundance

#binary_TPM<-TPMs[,samples_keep$ProcessingID]

#binary_TPM[binary_TPM<1]<-0

#binary_TPM[binary_TPM>=1]<-1

#binary_TPM<-as.data.frame(binary_TPM)

#binary_TPM$sums<-rowSums(binary_TPM)

#binary_TPM<-binary_TPM[which(binary_TPM$sums>=51),] #genes exp in all samples at least 1 TPM

#expressed_genes<-as.character(row.names(binary_TPM)) #print this list to read into other files

#xygenes_1_51<-txn_chr_info[txn_chr_info$info %in% as.character(expressed_genes),]

#xygenes_1_51<-xygenes_1_51[grep("X|Y",xygenes_1_51$chr),] ## 23 X genes, no Y genes

#xygenes_1_51.sum<-ddply(xygenes_1_51,.(chr),nrow)

TPMs<-txi$abundance

#par1_genes<-counttable[which(counttable$genename %in% c(par1,par2)),"geneinfo"]

binary_TPM<-TPMs[!(row.names(TPMs)%in%par1_genes),samples_keep$ProcessingID]

binary_TPM[binary_TPM<1]<-0

binary_TPM[binary_TPM>=1]<-1

binary_TPM<-as.data.frame(binary_TPM)

binary_TPM$sums<-rowSums(binary_TPM)

binary_TPM<-binary_TPM[which(binary_TPM$sums>=dim(binary_TPM)[2]/2),] #genes exp in half samples at least 1 TPM

expressed_genes<-as.character(row.names(binary_TPM)) #print this list to read into other files

xygenes_1_51<-txn_chr_info[txn_chr_info$info %in% as.character(expressed_genes),]

xygenes_1_51<-xygenes_1_51[grep("X|Y",xygenes_1_51$chr),]

xygenes_1_51.sum<-ddply(xygenes_1_51,.(chr),nrow)

#319 X genes, 4 Y genes

#how many X genes are from balaton list?

escape_list_balaton_2015<-c("ARSH","CXorf28","PRKX","HDHD1","STS","PNPLA4","FAM9C","ACE2","CA5BP1","ZRSR2","S100G","SYAP1","TXLNG","EIF1AX","EIF2S3","ZFX","VENTXP1","LOC347411","CXorf38","DDX3X","FUNDC1","UBA1","INE1","JPX","TAF7L","NXF5","ZCCHC16","GPR112","VGLL1","GYG2","ARSD","MXRA5","NLGN4X","KAL1","TCEANC","RAB9A","TRAPPC2","OFD1","GPM6B","GEMIN8","CA5B","AP1S2","CTPS2","RBBP7","USP9X","KDM6A","CDK16","KDM5C","IQSEC2","SMC1A","RPS4X","NAP1L3","AK026512","HTR2C","L1CAM")

X_expressedinhalf_escapee<-xygenes_1_51[xygenes_1_51$GENEID %in% escape_list_balaton_2015,]

X_expressedinhalf_escapee_TPMs<-TPMs[!(row.names(TPMs)%in%X_expressedinhalf_escapee$info),samples_keep$ProcessingID]

favorite_x_genes<-X_expressedinhalf_escapee_TPMs$info

#remove x genes and replace with escapee only

#expressed_genes<-rbind(expressed_genes_chr_A,xygenes_1_51)

#keep only remaining expressed genes (>=1TPM in at least 1 sample)

expressed_genes<-read.table("~/Dropbox/Rinnlab/manuscripts/EmbryoPaper2018/Review1/support_for_code/rsem_files/March2018_expressed_genes.tab")

expressed_genes<-expressed_genes$x

#expressed_genes_chr<-txn_chr_info[txn_chr_info$info %in% as.character(expressed_genes),]

#expressed_genes_chr_A<-expressed_genes_chr[grep("X|Y|M",expressed_genes_chr$chr,invert=TRUE),]

#expressed_genes_chrX<-expressed_genes_chr[grep("X",expressed_genes_chr$chr),]

#expressed_genes_chrY<-expressed_genes_chr[grep("Y",expressed_genes_chr$chr),]

#escape_list_balaton_2015<-c("ARSH","CXorf28","PRKX","HDHD1","STS","PNPLA4","FAM9C","ACE2","CA5BP1","ZRSR2","S100G","SYAP1","TXLNG","EIF1AX","EIF2S3","ZFX","VENTXP1","LOC347411","CXorf38","DDX3X","FUNDC1","UBA1","INE1","JPX","TAF7L","NXF5","ZCCHC16","GPR112","VGLL1","GYG2","ARSD","MXRA5","NLGN4X","KAL1","TCEANC","RAB9A","TRAPPC2","OFD1","GPM6B","GEMIN8","CA5B","AP1S2","CTPS2","RBBP7","USP9X","KDM6A","CDK16","KDM5C","IQSEC2","SMC1A","RPS4X","NAP1L3","AK026512","HTR2C","L1CAM")

##expressed_genes_chrX<-expressed_genes_chrX[grep(paste(escape_list_balaton_2015,collapse="|"),expressed_genes_chrX$GENEID),]

#expressed_genes_chrX<-expressed_genes_chrX[expressed_genes_chrX$GENEID%in%escape_list_balaton_2015,]

#expressed_genes<-rbind(expressed_genes_chr_A,expressed_genes_chrX,expressed_genes_chrY)

dds<-dds[row.names(dds)%in%expressed_genes$info,]

#remove mitochondrial reads here --much of this is now repetitive

counttable<-as.data.frame(counts(dds))

counttable$geneinfo<-row.names(counttable)

counttable$txname<-str_split_fixed(counttable$geneinfo,"_",2)[,1]

counttable$genename<-str_split_fixed(counttable$geneinfo,"_",2)[,2]

counttable_chr<-merge(counttable,txn_chr_info,by.x="txname",by.y="TXNAME")

mito<-counttable_chr[which(counttable_chr$chr=="chrM"),]

counttable<-counttable_chr[which(counttable_chr$chr!="chrM"),]

genomic_genes<-counttable$geneinfo

dds<-dds[row.names(dds)%in%genomic_genes,]

#remove PAR1 genes

par1<-c("PLCXD1","GTPBP6","PPP2R3B","SHOX","CRLF2","CSF2RA","IL3RA","SLC25A6","ASMTL","P2RY8","CXYorf3","ASMT","DHRSX","ZBED1","CD99","XG")

par2<-c("VAMP7","IL9R","WASH6P")

#PRY3, SYBL1, IL9R and CXYorf1 --> SYBL1 == VAMP7; CXYorf1 is WASH6P; cant find PRY3

par1_genes<-counttable[which(counttable$genename %in% c(par1,par2)),"geneinfo"]

dim(dds)

```

```{r quality_filter_xyupdate}

samples_keep<-read.table("~/Dropbox/Rinnlab/manuscripts/EmbryoPaper2018/Review1/support_for_code/rsem_files/March2018_samples_passing_QC.tab",header=TRUE)

#quality filtering

#remove genes with no counts

dds<-ddsTxi

nrow(dds)

dds <- dds[ rowSums(counts(dds)) > 1, ] #get rid of super noisy expression

nrow(dds)

dim(dds)

dds<-dds[,(colnames(dds)%in%samples_keep$ProcessingID)] #get rid of low expressing samples

dim(dds)

#how many X and Y genes kept?

txn_chr_info$info<-paste(txn_chr_info$TXNAME,txn_chr_info$GENEID,sep="_")

#1TMP, 1 sample -->

xygenes_1_1<-txn_chr_info[expressed_genes,]

xygenes_1_1<-xygenes_1_1[grep("X|Y",xygenes_1_1$chr),]

xygenes_1_1.sum<-ddply(xygenes_1_1,.(chr),nrow)

#1 chrX 902

#2 chrY 87

#TPMs<-txi$abundance

#binary_TPM<-TPMs[,samples_keep$ProcessingID]

#binary_TPM[binary_TPM<1]<-0

#binary_TPM[binary_TPM>=1]<-1

#binary_TPM<-as.data.frame(binary_TPM)

#binary_TPM$sums<-rowSums(binary_TPM)

#binary_TPM<-binary_TPM[which(binary_TPM$sums>=51),] #genes exp in all samples at least 1 TPM

#expressed_genes<-as.character(row.names(binary_TPM)) #print this list to read into other files

#xygenes_1_51<-txn_chr_info[txn_chr_info$info %in% as.character(expressed_genes),]

#xygenes_1_51<-xygenes_1_51[grep("X|Y",xygenes_1_51$chr),] ## 23 X genes, no Y genes

#xygenes_1_51.sum<-ddply(xygenes_1_51,.(chr),nrow)

TPMs<-txi$abundance

#par1_genes<-counttable[which(counttable$genename %in% c(par1,par2)),"geneinfo"]

binary_TPM<-TPMs[!(row.names(TPMs)%in%par1_genes),samples_keep$ProcessingID]

binary_TPM[binary_TPM<1]<-0

binary_TPM[binary_TPM>=1]<-1

binary_TPM<-as.data.frame(binary_TPM)

binary_TPM$sums<-rowSums(binary_TPM)

binary_TPM<-binary_TPM[which(binary_TPM$sums>=dim(binary_TPM)[2]/2),] #genes exp in half samples at least 1 TPM

expressed_genes<-as.character(row.names(binary_TPM)) #print this list to read into other files

xygenes_1_51<-txn_chr_info[txn_chr_info$info %in% as.character(expressed_genes),]

xygenes_1_51<-xygenes_1_51[grep("X|Y",xygenes_1_51$chr),]

xygenes_1_51.sum<-ddply(xygenes_1_51,.(chr),nrow)

#319 X genes, 4 Y genes

#how many X genes are from balaton list?

escape_list_balaton_2015<-c("ARSH","CXorf28","PRKX","HDHD1","STS","PNPLA4","FAM9C","ACE2","CA5BP1","ZRSR2","S100G","SYAP1","TXLNG","EIF1AX","EIF2S3","ZFX","VENTXP1","LOC347411","CXorf38","DDX3X","FUNDC1","UBA1","INE1","JPX","TAF7L","NXF5","ZCCHC16","GPR112","VGLL1","GYG2","ARSD","MXRA5","NLGN4X","KAL1","TCEANC","RAB9A","TRAPPC2","OFD1","GPM6B","GEMIN8","CA5B","AP1S2","CTPS2","RBBP7","USP9X","KDM6A","CDK16","KDM5C","IQSEC2","SMC1A","RPS4X","NAP1L3","AK026512","HTR2C","L1CAM")

X_expressedinhalf_escapee<-xygenes_1_51[xygenes_1_51$GENEID %in% escape_list_balaton_2015,]

X_expressedinhalf_escapee_TPMs<-TPMs[!(row.names(TPMs)%in%X_expressedinhalf_escapee$info),samples_keep$ProcessingID]

favorite_x_genes<-X_expressedinhalf_escapee_TPMs$info

specific_Y_genes_names<-c("EIF1AY", "DDX3Y", "RPS4Y1")

specific_Y_genes<-Ygenes[Ygenes$GENEID %in% specific_Y_genes_names,]

specific_Y_genes$info<-paste(specific_Y_genes$TXNAME,specific_Y_genes$GENEID,sep="_")

#keep only remaining expressed genes (>=1TPM in at least 1 sample)

expressed_genes<-read.table("~/Dropbox/Rinnlab/manuscripts/EmbryoPaper2018/Review1/support_for_code/rsem_files/March2018_expressed_genes.tab")

expressed_genes<-expressed_genes$x

expressed_genes_chr<-txn_chr_info[txn_chr_info$info %in% as.character(expressed_genes),]

expressed_genes_chr_A<-expressed_genes_chr[grep("X|Y|M",expressed_genes_chr$chr,invert=TRUE),]

#expressed_genes_chrX<-expressed_genes_chr[grep("X",expressed_genes_chr$chr),]

#expressed_genes_chrY<-expressed_genes_chr[grep("Y",expressed_genes_chr$chr),]

#escape_list_balaton_2015<-c("ARSH","CXorf28","PRKX","HDHD1","STS","PNPLA4","FAM9C","ACE2","CA5BP1","ZRSR2","S100G","SYAP1","TXLNG","EIF1AX","EIF2S3","ZFX","VENTXP1","LOC347411","CXorf38","DDX3X","FUNDC1","UBA1","INE1","JPX","TAF7L","NXF5","ZCCHC16","GPR112","VGLL1","GYG2","ARSD","MXRA5","NLGN4X","KAL1","TCEANC","RAB9A","TRAPPC2","OFD1","GPM6B","GEMIN8","CA5B","AP1S2","CTPS2","RBBP7","USP9X","KDM6A","CDK16","KDM5C","IQSEC2","SMC1A","RPS4X","NAP1L3","AK026512","HTR2C","L1CAM")

##expressed_genes_chrX<-expressed_genes_chrX[grep(paste(escape_list_balaton_2015,collapse="|"),expressed_genes_chrX$GENEID),]

#expressed_genes_chrX<-expressed_genes_chrX[expressed_genes_chrX$GENEID%in%escape_list_balaton_2015,]

expressed_genes<-rbind(expressed_genes_chr_A$info,favorite_x_genes,specific_Y_genes$info)

dds<-dds[row.names(dds)%in%expressed_genes,]

#remove mitochondrial reads here --much of this is now repetitive

counttable<-as.data.frame(counts(dds))

counttable$geneinfo<-row.names(counttable)

counttable$txname<-str_split_fixed(counttable$geneinfo,"_",2)[,1]

counttable$genename<-str_split_fixed(counttable$geneinfo,"_",2)[,2]

counttable_chr<-merge(counttable,txn_chr_info,by.x="txname",by.y="TXNAME")

mito<-counttable_chr[which(counttable_chr$chr=="chrM"),]

counttable<-counttable_chr[which(counttable_chr$chr!="chrM"),]

genomic_genes<-counttable$geneinfo

dds<-dds[row.names(dds)%in%genomic_genes,]

#remove PAR1 genes

par1<-c("PLCXD1","GTPBP6","PPP2R3B","SHOX","CRLF2","CSF2RA","IL3RA","SLC25A6","ASMTL","P2RY8","CXYorf3","ASMT","DHRSX","ZBED1","CD99","XG")

par2<-c("VAMP7","IL9R","WASH6P")

#PRY3, SYBL1, IL9R and CXYorf1 --> SYBL1 == VAMP7; CXYorf1 is WASH6P; cant find PRY3

par1_genes<-counttable[which(counttable$genename %in% c(par1,par2)),"geneinfo"]

dim(dds)

```

## Genotype by read counts

```{r genotype_by_readcounts_wholeembryo_COUNTS}

countdat<-as.data.frame(counts(dds))

countdat$info<-row.names(countdat)

geneinfo2<-str_split_fixed(countdat$info,"_",2)

countdat$txname<-geneinfo2[,1]

countdat$genename<-geneinfo2[,2]

countdat_chr<-merge(countdat,txn_chr_info,by.x="txname","TXNAME")

#count #genes per chr

genes_per_chr<-ddply(countdat_chr,~chr,nrow)

# number of genes -- how to see diffs across samples?/

countdat_chr.melt<-melt(countdat_chr)

############## if using counts, use this ##############

#sum counts per chr per sample

sum_counts_per_chr_per_sample<-acast(countdat_chr.melt,variable+chr~.,sum)

reads_per_sample<-as.data.frame(acast(countdat_chr.melt,variable~.,sum))

names(reads_per_sample)<-"sample_depth"

reads_per_sample$sampleid<-row.names(reads_per_sample)

sum_counts_per_chr_per_sample<-as.data.frame(sum_counts_per_chr_per_sample)

names(sum_counts_per_chr_per_sample)<-"count"

sum_counts_per_chr_per_sample$sample_chr<-row.names(sum_counts_per_chr_per_sample)

sum_counts_per_chr_per_sample$chr<-str_split_fixed(sum_counts_per_chr_per_sample$sample_chr,"_",2)[,2]

sum_counts_per_chr_per_sample$sample<-str_split_fixed(sum_counts_per_chr_per_sample$sample_chr,"_",2)[,1]

dat<-merge(sum_counts_per_chr_per_sample,metadata,by.x="sample",by.y="ProcessingID")

dat2<-merge(dat,reads_per_sample,by.x="sample",by.y="sampleid")

dat<-dat2

dat$normcount<-dat$count/dat$sample_depth

#ggplot hist counts sums per chr

#ggplot(dat,aes(count,color=Sampletype))+geom_histogram()+facet_wrap(~chr,scales="free_x")+theme_bw()

```

```{r stats_COUNTS}

# STATISTICAL TESTING...

# For now, calculate average and SD

# for sex chrs, should this be avg for males/females, or avg for all?

# sexless, by CHR!

# mean_normcount_nosex<-ddply(sexchroms[,c("chr","count")],.(chr),mean)

# sexchroms$sd_normcount_nosex<-sd(sexchroms$normcount)

mean_normcount<-aggregate(dat[,c("normcount")],by=list(dat$chr,dat$Sampletype),mean)

names(mean_normcount)<-c("chr","Sampletype","mean")

mean_normcount$sample_chr<-paste(mean_normcount$Sampletype,mean_normcount$chr,sep="_")

sd_normcount<-aggregate(dat[,c("normcount")],by=list(dat$chr,dat$Sampletype),sd)

names(sd_normcount)<-c("chr","Sampletype","sd")

sd_normcount$sample_chr<-paste(sd_normcount$Sampletype,sd_normcount$chr,sep="_")

meanandsd<-merge(mean_normcount,sd_normcount, by.x="sample_chr",by.y="sample_chr")

meanandsd<-meanandsd[,grep("[.]",names(meanandsd),invert=TRUE)]

dat$sampletype_chr<-paste(dat$Sampletype,dat$chr,sep="_")

dat2<-merge(dat,meanandsd,by.x="sampletype_chr",by.y="sample_chr")

dat<-dat2

dat$zscore<-(dat$normcount-dat$mean)/dat$sd

#ONLY SEX CHROMOSOMES

sexchroms<-dat[grep('X|Y',dat$chr),]

sexchroms<-sexchroms[grep("day3",sexchroms$Sampletype,invert=TRUE),]

```

```{r plots}

#sexchroms<-sexchroms[order(sexchroms$Sampletype,sexchroms$chr,sexchroms$zscore,decreasing=TRUE),]

sexchroms<-sexchroms[order(sexchroms$Sampletype,sexchroms$chr,sexchroms$zscore),]

sexchroms$PaperID<-factor(sexchroms$PaperID,levels=unique(sexchroms$PaperID))

TEBx<-subset(sexchroms,Sampletype=="trophectodermbiopsy")

#TEBx<-TEBx[order(TEBx$Sampletype,TEBx$chr,TEBx$zscore,decreasing=TRUE),]

TEBx<-TEBx[order(TEBx$Sampletype,TEBx$chr,TEBx$zscore),]

TEBx$PaperID<-factor(TEBx$PaperID,levels=unique(TEBx$PaperID))

pdf("SexZscoreDotPlots_sorted_TE_revision_stringentgenes.pdf")#changed back to normal

ggplot(TEBx,aes(PaperID,zscore,color=Sampletype))+geom_point()+theme_bw()+facet_wrap(~chr,scales="free")+ggtitle("TEBx")+theme(axis.text.x = element_text(angle = 90, hjust = 1))+scale_color_manual(values=c("day3"="light blue","trophectodermbiopsy"="grey","wholeembryo"="black"))

dev.off()

pdf("SexZscoreDotPlots_sorted_WE_revision_stringentgenes.pdf")#changed back to normal

ggplot(subset(sexchroms,Sampletype=="wholeembryo"),aes(PaperID,zscore,color=Sampletype))+geom_point()+theme_bw()+facet_wrap(~chr,scales="free")+ggtitle("WE")+theme(axis.text.x = element_text(angle = 90, hjust = 1))+scale_color_manual(values=c("day3"="light blue","trophectodermbiopsy"="grey","wholeembryo"="black"))

dev.off()

```

Assess agreement between euploid known WE and TE and then unknown WE and TE (only plot embryos w WE and TE)

For these, plot Y counts only

```{r AgreementPlots}

dat<-sexchroms

dat<-dat[order(dat$Sex),]

dat<-dat[grep("Y",dat$chr),]

dat$PaperID<-factor(dat$PaperID,levels=unique(dat$PaperID))

#number of samples per embryoID (plot only samples w 2)

countsamples<-ddply(dat,.(PaperID),nrow)

countsamples<-countsamples[which(countsamples$V1>1),]

dat<-dat[dat$PaperID %in% countsamples$PaperID,]

dat<-dat[order(dat$Sex,decreasing=TRUE),]

dat$label<-paste(dat$Sampletype,dat$Sex,sep="_")

dat$PaperID<-factor(dat$PaperID,levels=unique(dat$PaperID))

pdf("WETESexAgreement_dots_par1and2.pdf")

ggplot(dat,aes(PaperID,zscore,colour=Sampletype,group=PaperID))+geom_point(size=2)+theme_bw()+theme(axis.text.x = element_text(angle = 65, hjust = 1))+scale_color_manual(values=c("trophectodermbiopsy"="gray","wholeembryo"="black"))+geom_line(color="dark gray")

dev.off()

```

```{r find_Y_genes}

#E1: contains Y by PGT-A, but no evidence of Y-chr reads in WE RNAseq. We don’t have a TE sample for this for comparison, but this is consistent with a Y-chr loss event in the majority of the embryo.

#TE: E12 and E35 contain no Y, but by PGT-A contain Y – also consistent with Y loss.

#For E12 we don’t have a matching WE sample, but we do for E35 (which agrees with PGT-A).

#- look at Y TPMs for this sample vs validated XX and validated XY and also total read depth for these samples

#Look at E1, E12, E35, E24 (XY), and E15 (XX)

#samples_of_interest<-diff_metadata[diff_metadata$PaperID %in% c("E1","E12","E35","E24","E15"),]

#samples_of_interest<-samples_of_interest[grep("whole|troph",samples_of_interest$Sampletype),]

#Ygenes<-txn_chr_info[grep("Y",txn_chr_info$chr),]

samples_of_interest<-diff_metadata

samples_of_interest<-samples_of_interest[grep("whole|troph",samples_of_interest$Sampletype),]

potentially_tetraploid<-c("NRAG41","NRAG78","NRAG82","NRAG90","NRAG93","NRAG95","NRAG66")

samples_of_interest<-samples_of_interest[!(samples_of_interest$ProcessingID %in% potentially_tetraploid),]

Ygenes<-txn_chr_info[grep("Y",txn_chr_info$chr),]

Ygenes$info<-paste(Ygenes$TXNAME,Ygenes$GENEID,sep="_")

Ygenes<-Ygenes[grep(paste(c(par1,par2),collapse="|"),Ygenes$GENEID,invert=TRUE),] #remove PAR1/2 genes

#TPMS

TPMs_ofinterest<-txi$abundance[Ygenes$info,samples_of_interest$ProcessingID]

TPMs_ofinterest<-as.data.frame(TPMs_ofinterest[grep(paste(par1,collapse="|"),row.names(TPMs_ofinterest),invert=TRUE),])

binary_TPM_ofinterest<-TPMs_ofinterest

binary_TPM_ofinterest[binary_TPM_ofinterest<1]<-0

binary_TPM_ofinterest[binary_TPM_ofinterest>=1]<-1

#binary_TPM_ofinterest[binary_TPM_ofinterest<2]<-0

#binary_TPM_ofinterest[binary_TPM_ofinterest>=2]<-1

binary_TPM_ofinterest<-as.data.frame(binary_TPM_ofinterest)

binary_TPM_ofinterest$sums<-rowSums(binary_TPM_ofinterest)

binary_TPM_ofinterest<-binary_TPM_ofinterest[which(binary_TPM_ofinterest$sums>=dim(binary_TPM_ofinterest)[2]/2),] #genes exp in half samples at least 1 TPM

expressed_genes<-as.character(row.names(binary_TPM_ofinterest)) #print this list to read into other files

TPMs_ofinterest<-TPMs_ofinterest[expressed_genes,]

TPMs_ofinterest$info<-row.names(TPMs_ofinterest)

TPMs_ofinterest.melt<-melt(TPMs_ofinterest)

TPMs_ofinterest.melt.annot<-merge(TPMs_ofinterest.melt,samples_of_interest,by.x="variable",by.y="ProcessingID")

#boxplot to show expression of all Y genes

ggplot(TPMs_ofinterest.melt.annot, aes(info,value,color=Sampletype))+geom_boxplot()+theme_bw()+theme(axis.text.x = element_text(angle = 90, hjust = 1))

#ggplot(TPMs_ofinterest.melt.annot, aes(info,value,color=Sampletype))+geom_jitter()+theme_bw()+theme(axis.text.x = element_text(angle = 90, hjust = 1))

#ggplot(TPMs_ofinterest.melt.annot, aes(info,value,color=Sampletype))+geom_point()+theme_bw()+theme(axis.text.x = element_text(angle = 90, hjust = 1))

#specific_Y_genes_names<-c("PRKY", "NLGN4Y", "TBL1Y", "AMELY", "TMSB4Y", "EIF1AY", "ZFY", "USP9Y", "DDX3Y", "UTY", "TSPY", "KDM5D","RPS4Y")

specific_Y_genes_names<-c("EIF1AY", "DDX3Y", "RPS4Y1")

#specific_Y_genes_names<-c("EIF1AY", "DDX3Y", "RPS4Y1","RP11-65G9.1","DHRSX")

specific_Y_genes<-Ygenes[Ygenes$GENEID %in% specific_Y_genes_names,]

specific_Y_genes$info<-paste(specific_Y_genes$TXNAME,specific_Y_genes$GENEID,sep="_")

#TPMS

TPMs_ofinterest<-txi$abundance[specific_Y_genes$info,samples_of_interest$ProcessingID]

TPMs_ofinterest.melt<-melt(TPMs_ofinterest)

names(TPMs_ofinterest.melt)<-c("info","ProcessingID","TPM")

TPMs_ofinterest.melt.annot<-merge(TPMs_ofinterest.melt,samples_of_interest,by.x="ProcessingID",by.y="ProcessingID")

#3 of these genes are expressed >1 TPM in approximately half of our samples, indicating RNA-based evidence of a Y chromosome*** (EIF1AY, DDX3Y, RPS4Y1, INCLUDE A BOXPLOT OF EXPRESSION VALUES FOR EACH?).

#boxplot to show expression of all

ggplot(TPMs_ofinterest.melt.annot, aes(info,TPM,color=Sampletype))+geom_boxplot()+theme_bw()+theme(axis.text.x = element_text(angle = 90, hjust = 1))

ggplot(TPMs_ofinterest.melt.annot, aes(info,TPM,color=Sampletype))+geom_jitter()+theme_bw()+theme(axis.text.x = element_text(angle = 90, hjust = 1))

ggplot(TPMs_ofinterest.melt.annot, aes(info,TPM,color=Sampletype))+geom_point()+theme_bw()+theme(axis.text.x = element_text(angle = 90, hjust = 1))

ggplot(TPMs_ofinterest.melt.annot, aes(info,TPM,color=Sex))+geom_boxplot()+theme_bw()+theme(axis.text.x = element_text(angle = 90, hjust = 1))+facet_wrap(~Sampletype)

pdf("Fav_y_gene_exp_embryos.pdf")

#ggplot(TPMs_ofinterest.melt.annot, aes(info,TPM,color=Sampletype))+geom_point()+theme_bw()+theme(axis.text.x = element_text(angle = 90, hjust = 1))

ggplot(TPMs_ofinterest.melt.annot, aes(info,TPM,color=Sex))+geom_boxplot()+theme_bw()+theme(axis.text.x = element_text(angle = 90, hjust = 1))+facet_wrap(~Sampletype)+scale_color_manual(values=c("XX"="black","XY"="red","Unknown"="pink"))

dev.off()

#ggplot(TPMs_ofinterest.melt.annot, aes(PaperID,value))+geom_boxplot()+theme_bw()+facet_wrap(~Sampletype)

#ggplot(TPMs_ofinterest.melt.annot, aes(PaperID,log(value)))+geom_boxplot()+theme_bw()+facet_wrap(~Sampletype)

#ggplot(TPMs_ofinterest.melt.annot, aes(PaperID,value))+geom_boxplot()+theme_bw()+facet_wrap(~Sampletype)+ylim(c(0,200))

#gplot(TPMs_ofinterest.melt.annot, aes(PaperID,value))+geom_boxplot()+theme_bw()+facet_wrap(~Sampletype)+ylim(c(0,50))

```

Y-gene update: sum counts

```{r ycalls_all samples}

samples_of_interest<-diff_metadata

samples_of_interest<-samples_of_interest[grep("whole|troph",samples_of_interest$Sampletype),]

potentially_tetraploid<-c("NRAG41","NRAG78","NRAG82","NRAG90","NRAG93","NRAG95","NRAG66")

samples_of_interest<-samples_of_interest[!(samples_of_interest$ProcessingID %in% potentially_tetraploid),]

Ygenes<-txn_chr_info[grep("Y",txn_chr_info$chr),]

#specific_Y_genes_names<-c("EIF1AY", "DDX3Y", "RPS4Y1","RP11-65G9.1","DHRSX")

specific_Y_genes_names<-c("EIF1AY", "DDX3Y", "RPS4Y1")

specific_Y_genes<-Ygenes[Ygenes$GENEID %in% specific_Y_genes_names,]

specific_Y_genes$info<-paste(specific_Y_genes$TXNAME,specific_Y_genes$GENEID,sep="_")

#counts

#countstable<-as.data.frame(counts(dds))

#TPMs_ofinterest<-countstable[specific_Y_genes$info,]

#ygene_tpm_sums<-as.data.frame(colSums(TPMs_ofinterest))

#names(ygene_tpm_sums)<-c("ygene_sum")

#ygene_tpm_sums$ProcessingID<-row.names(ygene_tpm_sums)

#ygene_tpm_sums<-merge(ygene_tpm_sums,diff_metadata,by.x="ProcessingID",by.y="ProcessingID")

#ygene_tpm_sums<-ygene_tpm_sums[order(ygene_tpm_sums$ygene_sum),]

#ygene_tpm_sums$PaperID<-factor(ygene_tpm_sums$PaperID,levels=unique(ygene_tpm_sums$PaperID))

#pdf("Embryo_favorite_ygene_sums.pdf")

#ggplot(ygene_tpm_sums,aes(PaperID,ygene_sum,color=Sampletype))+geom_point()+theme_bw()+scale_color_manual(values=c("day3"="light blue","trophectodermbiopsy"="grey","wholeembryo"="black"))

#dev.off()

#ggplot(ygene_tpm_sums,aes(ygene_sum))+geom_histogram(bins=100)+theme_bw()+scale_color_manual(values=c("day3"="light blue","trophectodermbiopsy"="grey","wholeembryo"="black"))+facet_wrap(~Sampletype,scales="free_x")

#ggplot(ygene_tpm_sums,aes(ygene_sum))+geom_histogram(bins=100)+theme_bw()+scale_color_manual(values=c("day3"="light blue","trophectodermbiopsy"="grey","wholeembryo"="black"))+facet_wrap(~Sampletype,scales="free_x")+xlim(0,1000)

#TPMs_ofinterest$info<-row.names(TPMs_ofinterest)

#TPMs_ofinterest.melt<-melt(TPMs_ofinterest)

#TPMs_ofinterest.melt.annot<-merge(TPMs_ofinterest.melt,samples_of_interest,by.x="variable",by.y="ProcessingID")

##tmp<-TPMs_ofinterest.melt.annot[,c("PaperID","variable","info","value","Sampletype")]

#TPMs_ofinterest.melt.annot<-TPMs_ofinterest.melt.annot[order(TPMs_ofinterest.melt.annot$ygene_sum),]

#TPMs_ofinterest.melt.annot$PaperID<-factor(TPMs_ofinterest.melt.annot$PaperID,levels=unique(TPMs_ofinterest.melt.annot$PaperID))

#pdf("Embryo_fav_ygene_dotsandboxes.pdf")

#ggplot(TPMs_ofinterest.melt.annot, aes(PaperID,value,color=Sampletype))+geom_boxplot()+theme_bw()+scale_color_manual(values=c("day3"="light blue","trophectodermbiopsy"="grey","wholeembryo"="black"))

#ggplot(TPMs_ofinterest.melt.annot, aes(PaperID,value,color=Sampletype))+geom_point()+theme_bw()+scale_color_manual(values=c("day3"="light blue","trophectodermbiopsy"="grey","wholeembryo"="black"))

#ggplot(TPMs_ofinterest.melt.annot, aes(PaperID,value,color=Sampletype))+geom_boxplot()+geom_point()+theme_bw()+scale_color_manual(values=c("day3"="light blue","trophectodermbiopsy"="grey","wholeembryo"="black"))

#dev.off()

#ggplot(TPMs_ofinterest.melt.annot, aes(PaperID,log(value),color=Sampletype))+geom_boxplot()+theme_bw()

#ggplot(TPMs_ofinterest.melt.annot, aes(PaperID,value))+geom_boxplot()+theme_bw()+facet_wrap(~Sampletype)+ylim(c(0,200))

#ggplot(TPMs_ofinterest.melt.annot, aes(PaperID,value))+geom_boxplot()+theme_bw()+facet_wrap(~Sampletype)+ylim(c(0,50))

#TPMS

TPMs_ofinterest<-txi$abundance[specific_Y_genes$info,samples_of_interest$ProcessingID]

ygene_tpm_sums<-as.data.frame(colSums(TPMs_ofinterest[,1:54]))

names(ygene_tpm_sums)<-c("ygene_sum")

ygene_tpm_sums$ProcessingID<-row.names(ygene_tpm_sums)

ygene_tpm_sums<-merge(ygene_tpm_sums,diff_metadata,by.x="ProcessingID",by.y="ProcessingID")

ygene_tpm_sums<-ygene_tpm_sums[order(ygene_tpm_sums$ygene_sum),]

ygene_tpm_sums$PaperID<-factor(ygene_tpm_sums$PaperID,levels=unique(ygene_tpm_sums$PaperID))

#ggplot(ygene_tpm_sums,aes(PaperID,ygene_sum,color=Sampletype))+geom_point()+theme_bw()+scale_color_manual(values=c("day3"="light blue","trophectodermbiopsy"="grey","wholeembryo"="black"))

TE_ygene_tpmsums<-ygene_tpm_sums[grep("troph",ygene_tpm_sums$Sampletype),]

TE_ygene_tpmsums_means<-ddply(TE_ygene_tpmsums,.(Sex),summarize,meanTPM=mean(ygene_sum))

pdf("embryo_ygenesums_tpms_histograms.pdf")

ggplot(ygene_tpm_sums,aes(ygene_sum))+geom_histogram(bins=100)+theme_bw()+scale_color_manual(values=c("day3"="light blue","trophectodermbiopsy"="grey","wholeembryo"="black"))+facet_wrap(~Sampletype,scales="free_x")

ggplot(ygene_tpm_sums,aes(ygene_sum))+geom_histogram(bins=100)+theme_bw()+scale_color_manual(values=c("day3"="light blue","trophectodermbiopsy"="grey","wholeembryo"="black"))+facet_wrap(~Sampletype,scales="free_x")+xlim(0,100)

dev.off()

pdf("Embryo_favorite_ygene_sums_TPMS_clear.pdf")

ggplot(ygene_tpm_sums,aes(PaperID,ygene_sum,color=Sampletype))+geom_point()+theme_bw()+scale_color_manual(values=c("day3"="light blue","trophectodermbiopsy"="grey","wholeembryo"="black"))+facet_wrap(~Sampletype,scales="free_x")+geom_hline(yintercept=25)

dev.off()

ygene_tpm_sums_WETEpairs<-ygene_tpm_sums[ygene_tpm_sums$PaperID %in% countsamples$PaperID,]

pdf("embryo_wete_ysum_agreement.pdf")

ggplot(ygene_tpm_sums_WETEpairs,aes(PaperID,ygene_sum,color=Sampletype))+geom_point()+theme_bw()+scale_color_manual(values=c("day3"="light blue","trophectodermbiopsy"="grey","wholeembryo"="black"))

dev.off()

#TPMs_ofinterest$info<-row.names(TPMs_ofinterest)

#TPMs_ofinterest.melt<-melt(TPMs_ofinterest)

#TPMs_ofinterest.melt.annot<-merge(TPMs_ofinterest.melt,samples_of_interest,by.x="variable",by.y="ProcessingID")

#tmp<-TPMs_ofinterest.melt.annot[,c("PaperID","variable","info","value","Sampletype")]

#TPMs_ofinterest.melt.annot<-TPMs_ofinterest.melt.annot[order(TPMs_ofinterest.melt.annot$ygene_sum),]

#TPMs_ofinterest.melt.annot$PaperID<-factor(TPMs_ofinterest.melt.annot$PaperID,levels=unique(TPMs_ofinterest.melt.annot$PaperID))

#pdf("Embryo_fav_ygene_dotsandboxes.pdf")

#ggplot(TPMs_ofinterest.melt.annot, aes(PaperID,value,color=Sampletype))+geom_boxplot()+theme_bw()+scale_color_manual(values=c("day3"="light blue","trophectodermbiopsy"="grey","wholeembryo"="black"))

#ggplot(TPMs_ofinterest.melt.annot, aes(PaperID,value,color=Sampletype))+geom_point()+theme_bw()+scale_color_manual(values=c("day3"="light blue","trophectodermbiopsy"="grey","wholeembryo"="black"))

#ggplot(TPMs_ofinterest.melt.annot, aes(PaperID,value,color=Sampletype))+geom_boxplot()+geom_point()+theme_bw()+scale_color_manual(values=c("day3"="light blue","trophectodermbiopsy"="grey","wholeembryo"="black"))

#dev.off()

#ggplot(TPMs_ofinterest.melt.annot, aes(PaperID,log(value),color=Sampletype))+geom_boxplot()+theme_bw()

#ggplot(TPMs_ofinterest.melt.annot, aes(PaperID,value))+geom_boxplot()+theme_bw()+facet_wrap(~Sampletype)+ylim(c(0,200))

#ggplot(TPMs_ofinterest.melt.annot, aes(PaperID,value))+geom_boxplot()+theme_bw()+facet_wrap(~Sampletype)+ylim(c(0,50))

```

```{r plots}

#ygene_tpm_sums<-ygene_tpm_sums[order(ygene_tpm_sums$ygene_sum),]

#ygene_tpm_sums$PaperID<-factor(ygene_tpm_sums$PaperID,levels=unique(ygene_tpm_sums$PaperID))

sexchroms$PaperID<-factor(sexchroms$PaperID,levels=unique(ygene_tpm_sums$PaperID))

sexchroms<-sexchroms[order(sexchroms$PaperID),]

sexchroms$PaperID<-factor(sexchroms$PaperID,levels=unique(ygene_tpm_sums$PaperID))

#sexchroms<-sexchroms[order(sexchroms$Sampletype,sexchroms$chr,sexchroms$zscore,decreasing=TRUE),]

#sexchroms<-sexchroms[order(sexchroms$Sampletype,sexchroms$chr,sexchroms$zscore),]

#sexchroms$PaperID<-factor(sexchroms$PaperID,levels=unique(sexchroms$PaperID))

TEBx<-subset(sexchroms,Sampletype=="trophectodermbiopsy")

#TEBx<-TEBx[order(TEBx$Sampletype,TEBx$chr,TEBx$zscore,decreasing=TRUE),]

#TEBx<-TEBx[order(TEBx$Sampletype,TEBx$chr,TEBx$zscore),]

#TEBx$PaperID<-factor(TEBx$PaperID,levels=unique(TEBx$PaperID))

pdf("SexZscoreDotPlots_sorted_TE_revision_stringentgenes.pdf")#changed back to normal

ggplot(TEBx,aes(PaperID,zscore,color=Sampletype))+geom_point()+theme_bw()+facet_wrap(~chr,scales="free")+ggtitle("TEBx")+theme(axis.text.x = element_text(angle = 90, hjust = 1))+scale_color_manual(values=c("day3"="light blue","trophectodermbiopsy"="grey","wholeembryo"="black"))

dev.off()

pdf("SexZscoreDotPlots_sorted_WE_revision_stringentgenes.pdf")#changed back to normal

ggplot(subset(sexchroms,Sampletype=="wholeembryo"),aes(PaperID,zscore,color=Sampletype))+geom_point()+theme_bw()+facet_wrap(~chr,scales="free")+ggtitle("WE")+theme(axis.text.x = element_text(angle = 90, hjust = 1))+scale_color_manual(values=c("day3"="light blue","trophectodermbiopsy"="grey","wholeembryo"="black"))

dev.off()

```

update X zscore: escapee genes only [not used]

```{r x-genes-escape-zscore}

escape_list_balaton_2015<-c("ARSH","CXorf28","PRKX","HDHD1","STS","PNPLA4","FAM9C","ACE2","CA5BP1","ZRSR2","S100G","SYAP1","TXLNG","EIF1AX","EIF2S3","ZFX","VENTXP1","LOC347411","CXorf38","DDX3X","FUNDC1","UBA1","INE1","JPX","TAF7L","NXF5","ZCCHC16","GPR112","VGLL1","GYG2","ARSD","MXRA5","NLGN4X","KAL1","TCEANC","RAB9A","TRAPPC2","OFD1","GPM6B","GEMIN8","CA5B","AP1S2","CTPS2","RBBP7","USP9X","KDM6A","CDK16","KDM5C","IQSEC2","SMC1A","RPS4X","NAP1L3","AK026512","HTR2C","L1CAM")

samples_of_interest<-diff_metadata

samples_of_interest<-samples_of_interest[grep("whole|troph",samples_of_interest$Sampletype),]

Xgenes<-txn_chr_info[grep("X",txn_chr_info$chr),]

Xgenes_escapee<-Xgenes[grep(paste(escape_list_balaton_2015,collapse="|"),Xgenes$GENEID),]

Xgenes_escapee<-Xgenes[Xgenes$GENEID%in%escape_list_balaton_2015,]

#TPMS

TPMs_ofinterest<-txi$abundance[Xgenes_escapee$info,samples_of_interest$ProcessingID]

TPMs_ofinterest<-as.data.frame(TPMs_ofinterest[grep(paste(par1,collapse="|"),row.names(TPMs_ofinterest),invert=TRUE),])

xescape_gene_tpm_sums<-as.data.frame(colSums(TPMs_ofinterest[,1:54]))

names(xescape_gene_tpm_sums)<-c("xescape_gene_sum")

xescape_gene_tpm_sums$ProcessingID<-row.names(xescape_gene_tpm_sums)

xescape_gene_tpm_sums<-merge(xescape_gene_tpm_sums,diff_metadata,by.x="ProcessingID",by.y="ProcessingID")

xescape_gene_tpm_sums<-xescape_gene_tpm_sums[order(xescape_gene_tpm_sums$xescape_gene_sum),]

xescape_gene_tpm_sums$PaperID<-factor(xescape_gene_tpm_sums$PaperID,levels=unique(xescape_gene_tpm_sums$PaperID))

ggplot(xescape_gene_tpm_sums,aes(PaperID,xescape_gene_sum,color=Sampletype))+geom_point()+theme_bw()+scale_color_manual(values=c("day3"="light blue","trophectodermbiopsy"="grey","wholeembryo"="black"))

```

Whole Embryo Male-Female Analysis Notebook

DOUBLE CHECK IMPUTED CALLS AGAINST FIG2 --> remove E1

# Setup

```{r setup}

library(DESeq2)

library(tximport)

library(readr)

library(stringr)

library(ggplot2)

library(reshape2)

library(plyr)

library(dplyr)

rsem_count_dir<-"~/Dropbox/Rinnlab/manuscripts/EmbryoPaper2018/Review1/support_for_code/rsem_files/"

#make transcript to gene conversion file using dat1 from above

files <- list.files(rsem_count_dir,pattern="*.genes.results",full.names=TRUE)

filenames<-list.files(rsem_count_dir,pattern="*.genes.results")

filenames<-gsub(".genes.results","",filenames)

names(files) <- filenames

metadata<-read.table("/Users/abigailgroff/Dropbox/Rinnlab/manuscripts/EmbryoPaper2018/Review1/support_for_code/metadata_021219.txt",header=TRUE,stringsAsFactors = FALSE)

metadata<-metadata[grep("whole",metadata$Sampletype),]

#metadata<-metadata[grep("Euploid",metadata$Geno),]

txn_chr_info<-read.table("~/Dropbox/PageLab/Projects/SexDiffs/supporting/txn_chr_info.tab",header=TRUE)

txn_chr_info$info<-paste(txn_chr_info$TXNAME,txn_chr_info$GENEID,sep="_")

chr_sizes<-read.table("~/Dropbox/PageLab/Projects/SexDiffs/supporting/chrom_sizes.tab",header=TRUE)

tx2gene<-read.table("~/Dropbox/PageLab/Projects/SexDiffs/supporting/tx2gene.tab",header=TRUE)

metadata_abrev<-metadata[,c("ProcessingID","Sex","ImputedorKnownSex","PaperID")]

#remove E1 (M by PGTA, F by Y reads)

uncertain_sex_calls<-c("E1")

metadata<-metadata_abrev[!metadata_abrev$PaperID%in%uncertain_sex_calls,]

```

# Define and load samples WE

```{r definesamples_and_load, include=FALSE}

diff_metadata<-metadata

diff_files<-files[which(names(files)%in%diff_metadata$ProcessingID)]

txi <- tximport(diff_files, type="rsem", tx2gene=tx2gene,abundanceCol = TPM)#, reader=read_tsv)

rownames(diff_metadata)<-diff_metadata$ProcessingID

diff_metadata<-diff_metadata[order(diff_metadata$ProcessingID),]

#add 1 to all lengths in txi

#https://support.bioconductor.org/p/92763/

txi$length<-txi$length+1

ddsTxi <- DESeqDataSetFromTximport(txi, colData=diff_metadata,design=~ImputedorKnownSex)

```

# Quality Filter

Remove genes that are not expressed in any sample

- can add binary screening so must be expressed >1 in any one sample

```{r quality_filter}

#input ddsTxi, output dds

quality_filter<-function(ddsTxi){

expressed_genes<-read.table("~/Dropbox/Rinnlab/manuscripts/EmbryoPaper2018/Review1/support_for_code/rsem_files/March2018_expressed_genes.tab")

expressed_genes<-expressed_genes$x

samples_keep<-read.table("~/Dropbox/Rinnlab/manuscripts/EmbryoPaper2018/Review1/support_for_code/rsem_files/March2018_samples_passing_QC.tab",header=TRUE)

#quality filtering

#remove genes with no counts

dds<-ddsTxi

nrow(dds)

dds <- dds[ rowSums(counts(dds)) > 1, ] #get rid of super noisy expression

nrow(dds)

dim(dds)

dds<-dds[,(colnames(dds)%in%samples_keep$ProcessingID)] #get rid of low expressing samples

dim(dds)

#keep only remaining expressed genes (>=1TPM in at least 1 sample in full dataset)

dds<-dds[row.names(dds)%in%expressed_genes,]

#and now >1TPM in at least 2 of THESE samples

binary<-txi$abundance

binary<-binary[which(row.names(binary)%in%expressed_genes),]

binary[binary<1]<-0

binary[binary>1]<-1

genes_to_keep<-row.names(binary[which(rowSums(binary)>=2),])

dds<-dds[row.names(dds)%in%genes_to_keep,]

#remove mitochondrial reads here

counttable<-as.data.frame(counts(dds))

counttable$geneinfo<-row.names(counttable)

counttable$txname<-str_split_fixed(counttable$geneinfo,"_",2)[,1]

counttable$genename<-str_split_fixed(counttable$geneinfo,"_",2)[,2]

counttable_chr<-merge(counttable,txn_chr_info,by.x="txname",by.y="TXNAME")

mito<-counttable_chr[which(counttable_chr$chr=="chrM"),]

counttable<-counttable_chr[which(counttable_chr$chr!="chrM"),]

genomic_genes<-counttable$geneinfo

dds<-dds[row.names(dds)%in%genomic_genes,]

#remove PAR1 genes

par1<-c("PLCXD1","GTPBP6","PPP2R3B","SHOX","CRLF2","CSF2RA","IL3RA","SLC25A6","ASMTL","P2RY8","CXYorf3","ASMT","DHRSX","ZBED1","CD99","XG")

par2<-c("VAMP7","IL9R","WASH6P")

#PRY3, SYBL1, IL9R and CXYorf1 --> SYBL1 == VAMP7; CXYorf1 is WASH6P; cant find PRY3

par1_genes<-counttable[which(counttable$genename %in% c(par1,par2)),"geneinfo"]

dds<-dds[!(row.names(dds)%in%par1_genes),]

}

dds<-quality_filter(ddsTxi)

```

imputed WE from ALL harvest days.

```{r imputedWE_PCA}

vsd<-varianceStabilizingTransformation(dds)

par(mfrow=c(1, 2))

dds <- estimateSizeFactors(dds)

#plotPCA(vsd, intgroup = c("ImputedorKnownSex"))+ggtitle("PCA, WE imputed sex")+theme_bw()

```

Differential expression imputedsex WE

```{r WE_imputedsex_DiffExp}

dds<-DESeq(dds)

resultsNames(dds)

res<-results(dds,alpha=0.05)

ressig<-as.data.frame(res[which(res$padj<0.05),])

ressig$info<-row.names(ressig)

ressig$genenames<-str_split_fixed(ressig$info,"_",2)[,2]

ressig$txname<-str_split_fixed(ressig$info,"_",2)[,1]

comb_dat<-as.data.frame(merge(ressig,annot_short,by.x="txname","txname"))

dat<-as.data.frame(res)

dat$sig<-"no"

dat$sig[which(dat$padj<0.05)]<-"yes"

dat$info<-row.names(dat)

dat$genenames<-str_split_fixed(dat$info,"_",2)[,2]

dat$txname<-str_split_fixed(dat$info,"_",2)[,1]

#write.table(dat,file="062019_WE_XX-v-XY_diff.txt",sep="\t",quote=FALSE,row.names=FALSE,col.names=TRUE)

```

# Chromosome distribution of sig genes

```{r WE_imputedsex_chromosome_distribution_sig_genes}

sigdat<-merge(ressig,txn_chr_info,by.x="info","info")

sigdat<-merge(ressig,txn_chr_info,by.x="info","info")

write.table(sigdat,file="062219_WE_XX-v-XY_siggenes.txt",sep="\t",quote=FALSE,row.names=FALSE,col.names=TRUE)

sigdat$num<-str_split_fixed(sigdat$chr,"chr",2)[,2]

sigdat$num<-factor(dat$num,levels=c("1","2","3","4","5","6","7","8","9","10","11","12","13","14","15","16","17","18","19","20","21","22","X","Y"))

sigdat$chr<-factor(dat$chr,levels=dat$chr)

sigdat.auto<-sigdat[grep("X|Y",sigdat$chr,invert=TRUE),]

pdf("WE_imputedsex_chrgenedist.pdf")

ggplot(sigdat,aes(num))+geom_histogram(stat="count")+theme_bw()+theme(axis.text.x = element_text(angle = 90, hjust = 1))

dev.off()

```

---

title: "R Notebook"

output:

html_notebook: default

pdf_document: default

---

RNA karyotype- Fig3 and SupFig3

```{r setup}

library(DESeq2)

library(tximport)

library(readr)

library(stringr)

library(ggplot2)

library(reshape2)

library(plyr)

library(dplyr)

#rsem_count_dir<-"/Volumes/valor2/users/agroff/seq/humanEmbryo/quants/rsem/50bptrim"

rsem_count_dir<-"~/Dropbox/Rinnlab/manuscripts/EmbryoPaper2018/Review1/support_for_code/rsem_files/"

#make transcript to gene conversion file using dat1 from above

files <- list.files(rsem_count_dir,pattern="*.genes.results",full.names=TRUE)

filenames<-list.files(rsem_count_dir,pattern="*.genes.results")

filenames<-gsub(".genes.results","",filenames)

names(files) <- filenames

metadata<-read.table("~/Dropbox/Rinnlab/manuscripts/EmbryoPaper2018/Review1/FigsSup/metadata_021219.txt",header=TRUE,stringsAsFactors = FALSE)

txn_chr_info<-read.table("~/Dropbox/PageLab/Projects/SexDiffs/supporting/txn_chr_info.tab",header=TRUE)

chr_sizes<-read.table("~/Dropbox/PageLab/Projects/SexDiffs/supporting/chrom_sizes.tab",header=TRUE)

tx2gene<-read.table("/Volumes/solexa_page/agroff/seq/humanEmbryo/analysis_old/analysis/tx2gene.tab",header=TRUE)

annot_short<-read.table("~/Dropbox/Rinnlab/manuscripts/EmbryoPaper2018/Review1/FigsSup/embryo_transcript_annotation_gencodev19_hg19.tab")

potentially_tetraploid<-c("NRAG41","NRAG78","NRAG82","NRAG90","NRAG93","NRAG95","NRAG66")

metadata<-metadata[!metadata$ProcessingID%in%potentially_tetraploid,]

```

# Define and load samples

```{r definesamples_and_load, include=FALSE}

diff_files<-files[which(names(files)%in%metadata$ProcessingID)]

diff_metadata<-metadata

txi <- tximport(diff_files, type="rsem", tx2gene=tx2gene)

rownames(diff_metadata)<-diff_metadata$ProcessingID

diff_metadata<-diff_metadata[order(diff_metadata$ProcessingID),]

#add 1 to all lengths in txi

#https://support.bioconductor.org/p/92763/

txi$length<-txi$length+1

ddsTxi <- DESeqDataSetFromTximport(txi, colData=diff_metadata,design=~Sampletype)

```

# Quality Filter

Remove genes that are not expressed in any sample

- can add binary screening so must be expressed >1 in any one sample

```{r quality_filter}

expressed_genes<-read.table("/Users/abigailgroff/Dropbox/Rinnlab/manuscripts/EmbryoPaper2018/Review1/support_for_code/rsem_files/March2018_expressed_genes.tab",header=TRUE)

expressed_genes<-expressed_genes$x

samples_keep<-read.table("/Users/abigailgroff/Dropbox/Rinnlab/manuscripts/EmbryoPaper2018/Review1/support_for_code/rsem_files/March2018_samples_passing_QC.tab",header=TRUE)

#quality filtering

#remove genes with no counts

dds<-ddsTxi

nrow(dds)

dds <- dds[ rowSums(counts(dds)) > 1, ] #get rid of super noisy expression

nrow(dds)

dim(dds)

dds<-dds[,(colnames(dds)%in%samples_keep$ProcessingID)] #get rid of low expressing samples

dim(dds)

#keep only remaining expressed genes (>=1TPM in at least 1 sample)

dds<-dds[row.names(dds)%in%expressed_genes,]

#remove mitochondrial reads here

counttable<-as.data.frame(counts(dds))

counttable$geneinfo<-row.names(counttable)

counttable$txname<-str_split_fixed(counttable$geneinfo,"_",2)[,1]

counttable$genename<-str_split_fixed(counttable$geneinfo,"_",2)[,2]

counttable_chr<-merge(counttable,txn_chr_info,by.x="txname",by.y="TXNAME")

mito<-counttable_chr[which(counttable_chr$chr=="chrM"),]

counttable<-counttable_chr[which(counttable_chr$chr!="chrM"),]

genomic_genes<-counttable$geneinfo

dds<-dds[row.names(dds)%in%genomic_genes,]

#remove PAR1 genes

par1<-c("PLCXD1","GTPBP6","PPP2R3B","SHOX","CRLF2","CSF2RA","IL3RA","SLC25A6","ASMTL","P2RY8","CXYorf3","ASMT","DHRSXY","ZBED1","CD99","XG")

par1_genes<-counttable[which(counttable$genename %in% par1),"geneinfo"]

dds<-dds[!(row.names(dds)%in%par1_genes),]

dim(dds)

samples_remaining<-colnames(dds)

#filtered_metadata<-metadata[which(metadata$ProcessingID %in% samples_remaining),]

```

## Karyotype using read counts normalized by sample size and calc per sampletype (using all QC'd samples per sampletype)

```{r genotype_by_readcounts}

countdat<-as.data.frame(counts(dds))

countdat$info<-row.names(countdat)

geneinfo2<-str_split_fixed(countdat$info,"_",2)

countdat$txname<-geneinfo2[,1]

countdat$genename<-geneinfo2[,2]

countdat_chr<-merge(countdat,txn_chr_info,by.x="txname","TXNAME")

countdat_chr_save<-countdat_chr

#count #genes per chr

genes_per_chr<-ddply(countdat_chr,~chr,nrow)

# number of genes -- how to see diffs across samples?/

countdat_chr.melt<-melt(countdat_chr)

#sum counts per chr per sample

sum_counts_per_chr_per_sample<-acast(countdat_chr.melt,variable+chr~.,sum)

reads_per_sample<-as.data.frame(acast(countdat_chr.melt,variable~.,sum))

names(reads_per_sample)<-"sample_depth"

reads_per_sample$sampleid<-row.names(reads_per_sample)

sum_counts_per_chr_per_sample<-as.data.frame(sum_counts_per_chr_per_sample)

names(sum_counts_per_chr_per_sample)<-"count"

sum_counts_per_chr_per_sample$sample_chr<-row.names(sum_counts_per_chr_per_sample)

sum_counts_per_chr_per_sample$chr<-str_split_fixed(sum_counts_per_chr_per_sample$sample_chr,"_",2)[,2]

sum_counts_per_chr_per_sample$sample<-str_split_fixed(sum_counts_per_chr_per_sample$sample_chr,"_",2)[,1]

dat<-merge(sum_counts_per_chr_per_sample,metadata,by.x="sample",by.y="ProcessingID")

dat2<-merge(dat,reads_per_sample,by.x="sample",by.y="sampleid")

reads_per_sample_save<-reads_per_sample

dat<-dat2

dat$normcount<-dat$count/dat$sample_depth

dat$sampletype_chr<-paste(dat$Sampletype,dat$chr,sep="_")

```

```{r stats}

# STATISTICAL TESTING...

# For now, calculate average and SD

# for sex chrs, should this be avg for males/females, or avg for all?

# sexless, by CHR!

# mean_normcount_nosex<-ddply(sexchroms[,c("chr","count")],.(chr),mean)

# sexchroms$sd_normcount_nosex<-sd(sexchroms$normcount)

############### ALL SAMPLES BY SAMPLETYPE ###############

#calculate dat$mean by sampletype!!

mean_normcount<-aggregate(dat[,c("normcount")],by=list(dat$chr,dat$Sampletype),mean)

names(mean_normcount)<-c("chr","Sampletype","mean")

mean_normcount$sample_chr<-paste(mean_normcount$Sampletype,mean_normcount$chr,sep="_")

#calculate dat$sd by sampletype!!

sd_normcount<-aggregate(dat[,c("normcount")],by=list(dat$chr,dat$Sampletype),sd)

names(sd_normcount)<-c("chr","Sampletype","sd")

sd_normcount$sample_chr<-paste(sd_normcount$Sampletype,sd_normcount$chr,sep="_")

meanandsd<-merge(mean_normcount,sd_normcount, by.x="sample_chr",by.y="sample_chr")

meanandsd<-meanandsd[,grep("[.]",names(meanandsd),invert=TRUE)]

dat2<-merge(dat,meanandsd,by.x="sampletype_chr",by.y="sample_chr")

dat<-dat2

dat$zscore<-(dat$normcount-dat$mean)/dat$sd

dat$PaperID<-factor(dat$PaperID)

dat$sigpotential<-"no"

dat$sigpotential[which(abs(dat$zscore)>3)]<-"yes"

#zscore_var<-aggregate(dat[,c("zscore")],by=list(dat$sample),var)

#keep samples w variance <1.5?

dat$WEChrCall<-"eu"

dat$WEChrCall[which(dat$zscore>3)]<-"gain"

dat$WEChrCall[which(dat$zscore<(-3))]<-"loss"

dat<-dat[grep("X|Y",dat$chr,invert=TRUE),]

dat$PaperID<-factor(dat$PaperID, levels=dat$PaperID[order(dat$EmbryoNumber)])

dat$chrnum<-as.numeric(gsub("chr","",dat$chr))

dat$chr<-factor(dat$chr, levels=dat$chr[order(dat$chrnum)])

dat_save<-dat

ggplot(dat,aes(PaperID,zscore,color=Sampletype))+geom_point()+theme_bw()+facet_wrap(~chr)+theme(axis.text.x = element_text(angle = 65, hjust = 1))+scale_color_manual(values=c("day3"="light blue","trophectodermbiopsy"="grey","wholeembryo"="black"))

```

```{r distribution_of_normcountscores}

#ggplot(dat,aes(normcount))+geom_histogram()+theme_bw()+facet_wrap(~chr+Sampletype,scales="free_x")#+theme(axis.text.x = element_text(angle = 65, hjust = 1))+scale_color_manual(values=c("day3"="light blue","trophectodermbiopsy"="grey","wholeembryo"="black"))

ggplot(subset(dat,Sampletype=="wholeembryo"),aes(normcount))+geom_histogram()+theme_bw()+facet_wrap(~chr,scales="free_x")

ggplot(subset(dat,Sampletype=="wholeembryo"),aes(normcount))+geom_histogram()+theme_bw()+facet_wrap(~chr)

```

# plot all individual samples (by sample)

```{r plot_samples}

#WE

WE_dat<-dat[grep("whole",dat$Sampletype),]

#ggplot(WE_dat,aes(sample,chr,fill=sigpotential))+geom_tile(colour="white")+theme_bw()+facet_wrap(~Sampletype)+theme(axis.text.x = element_text(angle = 65, hjust = 1))+ggtitle("All Samples Heatmap By Sig")+scale_fill_manual(values=c("no"="black","yes"="red"))

#pdf("WE_karyotype_summary.pdf") #using 3 cutoff, no shuffle

#ggplot(WE_dat,aes(PaperID,chr,fill=WEChrCall))+geom_tile(colour="white")+theme_bw()+facet_wrap(~Sampletype)+theme(axis.text.x = element_text(angle = 65, hjust = 1))+ggtitle("WE Heatmap")+scale_fill_manual(values=c("eu"="black","gain"="dark red", "loss"="dark blue"))

#dev.off()

#TEBx

TE_dat<-dat[grep("troph",dat$Sampletype),]

#ggplot(TE_dat,aes(sample,chr,fill=sigpotential))+geom_tile(colour="white")+theme_bw()+facet_wrap(~Sampletype)+theme(axis.text.x = element_text(angle = 65, hjust = 1))+ggtitle("All Samples Heatmap By Sig")+scale_fill_manual(values=c("no"="gray","yes"="red"))

#pdf("TEBx_karyotype_summary.pdf")#using 3 cutoff, no shuffle

#ggplot(TE_dat,aes(sample,chr,fill=WEChrCall))+geom_tile(colour="white")+theme_bw()+facet_wrap(~Sampletype)+theme(axis.text.x = element_text(angle = 65, hjust = 1))+ggtitle("TE Heatmap")+scale_fill_manual(values=c("eu"="gray","gain"="red", "loss"="blue"))

#dev.off()

```

zscore range vs transcriptome coverage

```{r range-v-coverage}

#calculate range per sample

dat_table<-data.table(dat)

z_range<-ddply(dat_table,.(sample),summarise,Zmin=min(zscore),Zmax=max(zscore))

z_range$zrange<-z_range$Zmax-z_range$Zmin

#read in transcriptome coverage annotation

txn_coverage<-read.table("/Volumes/valor2/users/agroff/seq/humanEmbryo/analysis/transcriptome_capture_bysampletype.txt",header=TRUE,stringsAsFactors = FALSE)

comparedat<-merge(txn_coverage,z_range,by.x="ProcessingID",by.y="sample")

ggplot(comparedat,aes(percent_txome,zrange,color=Sampletype))+geom_point()+theme_bw()+scale_color_manual(values=c("trophectodermbiopsy"="gray","wholeembryo"="black"))

cor(comparedat$percent_txome,comparedat$zrange) #-0.6357873

fit<-lm(comparedat$percent_txome~comparedat$zrange)

summary(fit)

#compare to all exp genes

txn_coverage_expgenes<-read.table("/Volumes/valor2/users/agroff/seq/humanEmbryo/analysis/transcriptome_capture_allexpgenes_bysampletype.txt",header=TRUE,stringsAsFactors = FALSE)

comparedat_expgenes<-merge(txn_coverage_expgenes,z_range,by.x="ProcessingID",by.y="sample")

pdf("allgenes_zscorerange_v_txome_coverage.pdf")

ggplot(comparedat_expgenes,aes(zrange,percent_txome,color=Sampletype))+geom_point()+theme_bw()+scale_color_manual(values=c("trophectodermbiopsy"="gray","wholeembryo"="black"))

dev.off()

cor(comparedat_expgenes$percent_txome,comparedat_expgenes$zrange) #-0.1463933

fit<-lm(comparedat_expgenes$percent_txome~comparedat_expgenes$zrange)

summary(fit)

WEonly<-comparedat_expgenes[grep("wholeembryo",comparedat_expgenes$Sampletype),]

cor(WEonly$percent_txome,WEonly$zrange) #-0.5138228

fit<-lm(WEonly$percent_txome~WEonly$zrange)

summary(fit)

```

# Plot a set of individual embryos -- euploid, gain, loss

```{r individualgenoplot}

dat$chrnumeric<-str_split_fixed(dat$chr,"chr",2)[,2]

dat$chrnumeric<-as.numeric(dat$chrnumeric)

dat<-dat[order(dat$chrnumeric),]

dat$chrnumeric<-factor(dat$chrnumeric,levels=dat$chrnumeric)

#pdf("gainandlossexamples_ploidy.pdf",height=4,width=8) #3cutoff no shuffle--but ends up being the same..

ggplot(subset(dat,sample=="NRAG21"|sample=="NRAG28"|sample=="NRAG20"),aes(chrnumeric,zscore,colour=sigpotential))+geom_point()+theme_bw()+theme(axis.text.x = element_text(angle = 90, hjust = 1))+facet_wrap(~sample)+scale_colour_manual(values=c("no"="black","yes"="red"))

#dev.off()

```

# bootstrap zscores

- how many of these chr zscores are unique given the transcriptome of this sample? --> indication of true aneuploidy

- how many samples are just super noisy -- separately, this may be an indication of error (ie complex or gross unregulated expression which will lead to senescence/death rather than a coordinated program of development)

```{r bootstrapzscores}

orig_dat<-dat_save

countdat_chr<-countdat_chr_save

countdat_chr<-countdat_chr[,grep("NRAG*|chr",colnames(countdat_chr))]

reads_per_sample<-reads_per_sample_save

shuffled_countdat<-countdat_chr

#nshuffle

nshuffle=3000

#input shuffled_countdat #(merge(countdat,txn_chr_info,by.x="txname","TXNAME")), sans txname etc. info only.

set.seed(1) #didnt do this on first 10k shuffle -_-

shuffle_zscores<-function(shuffled_countdat,nshuffle,reads_per_sample,orig_dat){

zscore_shuffle_metadata<-data.frame()

zscore_shuffles<-list()

for (i in (seq(1:nshuffle))){

#shuffle labels of shuffled_countdat (countdat_chr)

shuffled_countdat$chr<-sample(shuffled_countdat$chr)

countdat_chr.melt<-melt(shuffled_countdat) #melt for ease summing

#sum counts per chr per sample

sum_counts_per_chr_per_sample<-acast(countdat_chr.melt,variable+chr~.,sum)

sum_counts_per_chr_per_sample<-as.data.frame(sum_counts_per_chr_per_sample)

names(sum_counts_per_chr_per_sample)<-"count"

sum_counts_per_chr_per_sample$sample_chr<-row.names(sum_counts_per_chr_per_sample)

sum_counts_per_chr_per_sample$chr<-str_split_fixed(sum_counts_per_chr_per_sample$sample_chr,"_",2)[,2]

sum_counts_per_chr_per_sample$sample<-str_split_fixed(sum_counts_per_chr_per_sample$sample_chr,"_",2)[,1]

dat_curr<-merge(sum_counts_per_chr_per_sample,metadata,by.x="sample",by.y="ProcessingID")

dat_curr2<-merge(dat_curr,reads_per_sample,by.x="sample",by.y="sampleid")

dat_curr<-dat_curr2

dat_curr$normcount<-dat_curr$count/dat_curr$sample_depth

dat_curr$sampletype_chr<-paste(dat_curr$Sampletype,dat_curr$chr,sep="_")

############### ALL SAMPLES BY SAMPLETYPE ###############

#calculate dat_curr$mean by sampletype!!

mean_normcount_curr<-aggregate(dat_curr[,c("normcount")],by=list(dat_curr$chr,dat_curr$Sampletype),mean)

names(mean_normcount_curr)<-c("chr","Sampletype","mean")

mean_normcount_curr$sample_chr<-paste(mean_normcount_curr$Sampletype,mean_normcount_curr$chr,sep="_")

#calculate dat_curr$sd by sampletype!!

sd_normcount_curr<-aggregate(dat_curr[,c("normcount")],by=list(dat_curr$chr,dat_curr$Sampletype),sd)

names(sd_normcount_curr)<-c("chr","Sampletype","sd")

sd_normcount_curr$sample_chr<-paste(sd_normcount_curr$Sampletype,sd_normcount_curr$chr,sep="_")

meanandsd_curr<-merge(mean_normcount_curr,sd_normcount_curr, by.x="sample_chr",by.y="sample_chr")

meanandsd_curr<-meanandsd_curr[,grep("[.]",names(meanandsd_curr),invert=TRUE)]

dat_curr2<-merge(dat_curr,meanandsd_curr,by.x="sampletype_chr",by.y="sample_chr")

dat_curr<-dat_curr2

dat_curr$zscore<-(dat_curr$normcount-dat_curr$mean)/dat_curr$sd

dat_curr<-dat_curr[grep("X|Y",dat_curr$chr,invert=TRUE),]

dat_curr<-dat_curr[order(dat_curr$sample_chr),]

zscore_shuffles[[i]]<-as.data.frame(dat_curr$zscore)

zscore_shuffle_metadata<-dat_curr[,c("sampletype_chr","sample","sample_chr","chr","PaperID","Sampletype")]

dat_curr$PaperID<-factor(dat_curr$PaperID)

dat_curr$sigpotential<-"no"

dat_curr$sigpotential[which(abs(dat_curr$zscore)>3)]<-"yes"

dat_curr$PaperID<-factor(dat_curr$PaperID, levels=dat_curr$PaperID[order(dat_curr$ReadibleEmbryoNumber)])

dat_curr$chrnum<-as.numeric(gsub("chr","",dat_curr$chr))

dat_curr$chr<-factor(dat_curr$chr, levels=dat_curr$chr[order(dat_curr$chrnum)])

ggplot(dat_curr,aes(chr,zscore,colour=Sampletype,shape=sigpotential))+geom_point()+theme_bw()+facet_wrap(~sample)+theme(axis.text.x = element_text(angle = 65, hjust = 1))+ggtitle("All Samples")+scale_color_manual(values=c("day3"="light blue","trophectodermbiopsy"="grey","wholeembryo"="black"))

}

#collect all shuffled zscores

zscore_shuffles_dat<-do.call("cbind",zscore_shuffles)

names(zscore_shuffles_dat)<-paste("zscore",seq(1:i),sep="_")

#associate to their meta-data (zscore_shuffle_metadata)

zscores_dat<-cbind(zscore_shuffle_metadata,zscore_shuffles_dat)

#combine with true sample zscore

orig_dat<-orig_dat[order(orig_dat$sample_chr),]

zscores_dat$true_zscore<-orig_dat$zscore

zscores_dat

}

#zdat<-shuffle_zscores(shuffled_countdat,nshuffle,reads_per_sample,orig_dat)

#save(zdat,file="~/Google Drive/Embryo/Figures/code/current_working/embryo_shuffled_zscores.Rdata")

load("~/Google Drive/Embryo/Figures/code/current_working/embryo_shuffled_zscores.Rdata")

#non-zscore-cols

zdatinfo<-grep("zscore",names(zdat),invert=TRUE,value=TRUE)

# where does true z score lie within the distribution of zscores for this sample_chr?

# start with examples from above sample=="NRAG21"|sample=="NRAG28"|sample=="NRAG20")

zdat_examples<-zdat[grep("NRAG21|NRAG28|NRAG20",zdat$sample),]

zdat_examples.melt<-melt(zdat_examples,id.vars=zdatinfo)

NRAG28_chr4<-zdat_examples.melt[which(zdat_examples.melt$sample_chr=="NRAG28_chr4"),]

NRAG28_chr4_true<-NRAG28_chr4[grep("true",NRAG28_chr4$variable),]

ggplot(NRAG28_chr4,aes(value))+geom_density()+theme_bw()+geom_vline(aes(xintercept=NRAG28_chr4_true$value))

get_sample_chr_dist<-function(sample,chr,zdat){

zdat_curr<-zdat[grep(sample,zdat$sample),]

zdat_curr.melt<-melt(zdat_curr,id.vars=zdatinfo)

s_curr<-zdat_curr.melt[which(zdat_curr.melt$sample_chr==paste(sample,chr,sep="_")),]

s_curr_true<-s_curr[grep("true",s_curr$variable),]

ggplot(s_curr,aes(value))+geom_density()+theme_bw()+geom_vline(aes(xintercept=s_curr_true$value),color="red")+ggtitle(paste(sample,chr,sep=" "))

}

NRAG28_chr1_plot<-get_sample_chr_dist("NRAG28","chr1",zdat)

NRAG28_chr4_plot<-get_sample_chr_dist("NRAG28","chr4",zdat)

NRAG28_chr18_plot<-get_sample_chr_dist("NRAG28","chr18",zdat)

NRAG28_chr16_plot<-get_sample_chr_dist("NRAG28","chr16",zdat)

NRAG21_chr1_plot<-get_sample_chr_dist("NRAG21","chr1",zdat)

NRAG21_chr4_plot<-get_sample_chr_dist("NRAG21","chr4",zdat)

NRAG21_chr16_plot<-get_sample_chr_dist("NRAG21","chr16",zdat)

NRAG20_chr4_plot<-get_sample_chr_dist("NRAG20","chr4",zdat)

NRAG20_chr16_plot<-get_sample_chr_dist("NRAG20","chr16",zdat)

NRAG96_chr1_plot<-get_sample_chr_dist("NRAG96","chr1",zdat)

NRAG96_chr15_plot<-get_sample_chr_dist("NRAG96","chr15",zdat)

pdf("NRAG28_chr4_dist.pdf")

NRAG28_chr4_plot

dev.off()

pdf("NRAG28_chr16_dist.pdf")

NRAG28_chr16_plot

dev.off()

pdf("NRAG21_chr16.pdf")

NRAG21_chr16_plot

dev.off()

pdf("NRAG21_chr4.pdf")

NRAG21_chr4_plot

dev.off()

pdf("NRAG20_chr4.pdf")

NRAG20_chr4_plot

dev.off()

pdf("NRAG20_chr16.pdf")

NRAG20_chr16_plot

dev.off()

# COUNT ZSCORES, CALC EMPIRICAL PVALUE AND MTC.

zscore_mat<-zdat[,(length(zdatinfo)+1):((length(zdatinfo)+1)+nshuffle-1)]

zscore_mat_binary<-zscore_mat

zscore_mat_binary<-apply(zscore_mat_binary,2,function(x){

#compare each column to true_zscore and decide if each occurance is more or less extreme than true_zscore

x_new<-x

x_new[abs(x)<abs(zdat$true_zscore)]<-0

x_new[abs(x)>=abs(zdat$true_zscore)]<-1

x_new

})

zscore_empirical_pvalue<-rowSums(zscore_mat_binary)/nshuffle

zdat$zscore_pval<-zscore_empirical_pvalue

zdat_short<-zdat[,c(zdatinfo,"true_zscore","zscore_pval")]

# BH correction

# put pvalues in ascending order

zdat_short<-zdat_short[order(zdat_short$zscore_pval),]

num_tests<-dim(zdat_short)[1]

# assign ranks

zdat_short$rank<-seq(1:num_tests)

# smallest = 1, largest = M

# BHQvalue= (i/m)Q --> i = rank, m = total # tests, Q = FDR ie 0.05 = 5%

FDR=0.05

zdat_short$qval<-((zdat_short$rank/num_tests)*FDR)

# compare p to q and find pargest p less than q. everything above this is significant!

zdat_short$sig<-"no"

plessthanq<-zdat_short$qval-zdat_short$zscore_pval #highest ranking positive val

#if any pos values in plessthanq:

if(any(plessthanq>0)){

cutoff_index<-max(which(plessthanq>0))

zdat_short$sig[1:cutoff_index]<-"yes" #everything above this is significant!

}

zdat_short$chrnum<-as.numeric(gsub("chr","",zdat_short$chr))

zdat_short<-zdat_short[order(zdat_short$chrnum),]

zdat_short$chr<-factor(zdat_short$chr,levels=zdat_short$chr[order(zdat_short$chrnum)])

zdat_short_annot<-merge(zdat_short,metadata,by.x="sample",by.y="ProcessingID")

pdf("allsamples_zscoreplots.pdf")

ggplot(zdat_short_annot,aes(chr,true_zscore,colour=Sampletype.y))+geom_point()+theme_bw()+facet_wrap(~PaperID)+theme(axis.text.x = element_text(angle = 90, hjust = 1))+ggtitle("All Samples")+scale_color_manual(values=c("day3"="light blue","trophectodermbiopsy"="grey","wholeembryo"="black"))

dev.off()

```

Heatmap summary from shuffled zscore karyotypes for WHOLE EMBRYOS - in paper use zscore cutoff as permisive and pvalcall as stringent (no mtc)

```{r shuffled_zscore_heatmap}

zdat_short$Call<-"normal"

zdat_short$Call[which(zdat_short$sig=="yes"&zdat_short$true_zscore<0)]<-"loss"

zdat_short$Call[which(zdat_short$sig=="yes"&zdat_short$true_zscore>0)]<-"gain"

#XXXXXXXXXXXXXXXXXXXXXXX WHATS GOING ON HERE? XXXXXXXXXXXXXXXX

filtered_metadata<-metadata[which(metadata$ReadableEmbryoID %in% zdat$ReadableEmbryoID),]

zdat_short<-merge(zdat_short,filtered_metadata[,c("ReadableEmbryoID","PaperID","EmbryoNumber")],)

#XXXXXXXXXXXXXXXXXXXXXXX WHATS GOING ON HERE? XXXXXXXXXXXXXXXX

zdat_short_WE<-zdat_short[grep("whole",zdat_short$Sampletype),]

zdat_short_WE$Embryonum<-as.numeric(gsub("E","",zdat_short_WE$PaperID))

zdat_short_WE$PaperID<-factor(zdat_short_WE$PaperID,levels=zdat_short_WE$PaperID[order(zdat_short_WE$Embryonum)])

zdat_short_WE$chrnum<-as.numeric(gsub("chr","",zdat_short_WE$chr))

zdat_short_WE$chr<-factor(zdat_short_WE$chr,levels=zdat_short_WE$chr[order(zdat_short_WE$chrnum)])

zdat_short_WE<-ddply(zdat_short_WE,.(sample_chr),head,1)

#pdf("WE_heatmap_shufflezscoresig_3000.pdf") #using corrected shuffled pvals

ggplot(zdat_short_WE,aes(PaperID,chr,fill=Call))+geom_tile(colour="white")+theme_bw()+facet_wrap(~Sampletype)+theme(axis.text.x = element_text(angle = 90, hjust = 1))+ggtitle("WE Heatmap")+scale_fill_manual(values=c("normal"="gray","gain"="dark red", "loss"="dark blue"))#uses MTC zscore and old embryo ID

#using uncorrected pval

zdat_short_WE$PvalCall<-"normal"

zdat_short_WE$PvalCall[which(zdat_short_WE$zscore_pval<0.01&zdat_short_WE$true_zscore<0)]<-"loss"

zdat_short_WE$PvalCall[which(zdat_short_WE$zscore_pval<0.01&zdat_short_WE$true_zscore>0)]<-"gain"

ggplot(zdat_short_WE,aes(PaperID,chr,fill=PvalCall))+geom_tile(colour="white")+theme_bw()+facet_wrap(~Sampletype)+theme(axis.text.x = element_text(angle = 90, hjust = 1))+ggtitle("WE Heatmap")+scale_fill_manual(values=c("normal"="gray","gain"="dark red", "loss"="dark blue"))

#zscore call -- most permissive at a glance!

zdat_short_WE$ZCall<-"normal"

zdat_short_WE$ZCall[which(zdat_short_WE$true_zscore<(-2))]<-"loss"

zdat_short_WE$ZCall[which(zdat_short_WE$true_zscore>2)]<-"gain"

pdf("WE_zscores_RNAploidy_permissive.pdf")

ggplot(zdat_short_WE,aes(PaperID,chr,fill=ZCall))+geom_tile(colour="white")+theme_bw()+facet_wrap(~Sampletype)+theme(axis.text.x = element_text(angle = 90, hjust = 1))+ggtitle("WE Heatmap")+scale_fill_manual(values=c("normal"="gray","gain"="dark red", "loss"="dark blue"))

dev.off()

#stringent used in paper: (not MTC, but use bootstrapped pval)

#PvalCall

pdf("WE_ploidy_summary_stringent_notmtc.pdf")

ggplot(zdat_short_WE,aes(PaperID,chr,fill=PvalCall))+geom_tile(colour="white")+theme_bw()+facet_wrap(~Sampletype)+theme(axis.text.x = element_text(angle = 90, hjust = 1))+ggtitle("WE Heatmap")+scale_fill_manual(values=c("normal"="gray","gain"="dark red", "loss"="dark blue"))

dev.off()

# permissive is Zcall

# uncorrected shuffle pval is PvalCall

#number gain

length(which(zdat_short_WE$ZCall=="gain")) #20 #permissive

length(which(zdat_short_WE$PvalCall=="gain")) #12

#number loss

length(which(zdat_short_WE$ZCall=="loss")) #15 #permissive

length(which(zdat_short_WE$PvalCall=="loss")) #4

# gain or loss num embryos

aneuploid_affected_permissive<-zdat_short_WE[grep("normal",zdat_short_WE$ZCall,invert=TRUE),] #permissive

length(unique(aneuploid_affected_permissive$PaperID))#15

aneuploid_affected_uncorrectedshuffle<-zdat_short_WE[grep("normal",zdat_short_WE$PvalCall,invert=TRUE),]

length(unique(aneuploid_affected_uncorrectedshuffle$PaperID))#12

```

Heatmap summary from shuffled zscore karyotypes for TEBX

```{r TEBX_shuffled_zscore_heatmap}

zdat_short_TE<-zdat_short[grep("troph",zdat_short$Sampletype),]

zdat_short_TE$Embryonum<-as.numeric(gsub("E","",zdat_short_TE$PaperID))

zdat_short_TE$PaperID<-factor(zdat_short_TE$PaperID,levels=zdat_short_TE$PaperID[order(zdat_short_TE$Embryonum)])

zdat_short_TE$chrnum<-as.numeric(gsub("chr","",zdat_short_TE$chr))

zdat_short_TE$chr<-factor(zdat_short_TE$chr,levels=zdat_short_TE$chr[order(zdat_short_TE$chrnum)])

zdat_short_TE<-ddply(zdat_short_TE,.(sample_chr),head,1)

#using uncorrected pval #paper stringent

zdat_short_TE$PvalCall<-"normal"

zdat_short_TE$PvalCall[which(zdat_short_TE$zscore_pval<0.01&zdat_short_TE$true_zscore<0)]<-"loss"

zdat_short_TE$PvalCall[which(zdat_short_TE$zscore_pval<0.01&zdat_short_TE$true_zscore>0)]<-"gain"

#zscore call -- paper permissive

zdat_short_TE$ZCall<-"normal"

zdat_short_TE$ZCall[which(zdat_short_TE$true_zscore<(-2))]<-"loss"

zdat_short_TE$ZCall[which(zdat_short_TE$true_zscore>2)]<-"gain"

pdf("TE_zscores_RNAploidy_permissive.pdf")

ggplot(zdat_short_TE,aes(PaperID,chr,fill=ZCall))+geom_tile(colour="white")+theme_bw()+facet_wrap(~Sampletype)+theme(axis.text.x = element_text(angle = 90, hjust = 1))+ggtitle("TE Heatmap")+scale_fill_manual(values=c("normal"="gray","gain"="dark red", "loss"="dark blue"))

dev.off()

#stringent used in paper: (not MTC, but use bootstrapped pval)

#PvalCall

pdf("TE_ploidy_summary_stringent_notmtc.pdf")

ggplot(zdat_short_TE,aes(PaperID,chr,fill=PvalCall))+geom_tile(colour="white")+theme_bw()+facet_wrap(~Sampletype)+theme(axis.text.x = element_text(angle = 90, hjust = 1))+ggtitle("TE Heatmap")+scale_fill_manual(values=c("normal"="gray","gain"="dark red", "loss"="dark blue"))

dev.off()

```

generate imputed ploidy call using most permissive zscore method (+/- zscore 2)

```{r RNA_ploidy_call}

zdat_short_WE_ploidysummary<-ddply(zdat_short_WE,.(PaperID,ZCall),nrow)

zdat_short_WE_ploidysummary<-zdat_short_WE_ploidysummary[grep("normal",zdat_short_WE_ploidysummary$ZCall),]

zdat_short_WE_ploidysummary$ploidy<-"NA"

zdat_short_WE_ploidysummary$ploidy[zdat_short_WE_ploidysummary$V1==22]<-"euploid"

zdat_short_WE_ploidysummary$ploidy[zdat_short_WE_ploidysummary$V1!=22]<-"aneuploid"

summaryOfPloidy<-ddply(zdat_short_WE_ploidysummary,.(ploidy),nrow)

zdat_short_WE_ploidysummary$ZCall<-NULL

zdat_short_WE_ploidysummary$V1<-NULL

names(zdat_short_WE_ploidysummary)<-c("PaperID","RNAPloidy")

#metadata<-merge(metadata,zdat_short_WE_ploidysummary,by.x="PaperID",by.y="PaperID")

#write.table(metadata,file="/Volumes/valor2/users/agroff/seq/humanEmbryo/analysis/032018_WE_ploidy_metadata.txt",quote = FALSE, col.names = TRUE,row.names = FALSE)

```

highlight those samples with an abnormal distribution of shuffled zscores (i think these would be noisy right? ... possibly complex abnormal etx) -- how do you measure this? start by plotting zscores for all chrs of a sample by sample

```{r shuffled_zscore_distributions}

zdat.melt<-melt(zdat,id.vars=zdatinfo)

shuffled_zdat.melt<-zdat.melt[grep("true",zdat.melt$variable,invert=TRUE),]

#ggplot(shuffled_zdat.melt,aes(sample,value,fill=Sampletype))+geom_violin()+theme_bw()+theme(axis.text.x = element_text(angle = 90, hjust = 1))+scale_fill_manual(values=c("day3"="light blue","trophectodermbiopsy"="grey","wholeembryo"="black"))+ggtitle("Shuffled Zscore Only spread by sampletype--measure of txn noise")

shuffled_zdat.melt2<-shuffled_zdat.melt

shuffled_zdat.melt2$ReadableEmbryoID<-NULL

shuffled_zdat.melt2$sampletype_chr<-NULL

ID_annotation<-metadata[,c("ProcessingID","PaperID","EmbryoNumber")]

shuffled_zdat.melt_IDannot<-merge(ID_annotation,shuffled_zdat.melt2,by.x="ProcessingID",by.y="sample")

shuffled_zdat.melt_IDannot<-shuffled_zdat.melt_IDannot[order(shuffled_zdat.melt_IDannot$EmbryoNumber),]

shuffled_zdat.melt_IDannot$PaperID<-factor(shuffled_zdat.melt_IDannot$PaperID,levels=shuffled_zdat.melt_IDannot$PaperID)

pdf("3000shuffled_zscore_distributions_by_sampletype_noiseanalysis.pdf")

ggplot(shuffled_zdat.melt_IDannot,aes(PaperID,value,fill=Sampletype))+geom_violin()+theme_bw()+theme(axis.text.x = element_text(angle = 90, hjust = 1))+scale_fill_manual(values=c("day3"="light blue","trophectodermbiopsy"="grey","wholeembryo"="black"))+ggtitle("Shuffled Zscore Only spread by sampletype--measure of txn noise")

dev.off()

# yeah NRAG35 has largest spread.. (know this one is complex abnormal)

# NRAG35, 45, 67, 71, 96 are noisiest

#true_zdat.melt<-zdat.melt[grep("true",zdat.melt$variable),]

#ggplot(true_zdat.melt,aes(sample,value,fill=Sampletype))+geom_violin()+theme_bw()+theme(axis.text.x = element_text(angle = 90, hjust = 1))+scale_fill_manual(values=c("day3"="light blue","trophectodermbiopsy"="grey","wholeembryo"="black"))+ggtitle("True Zscore spread by sampletype--measure of txn noise")

```

# Correlation of zscore profiles across samples

```{r zscore_correlations}

# chr_zscore x sample matrix --> sample x sample cor values

cor_dat<-orig_dat[grep("troph|whole",orig_dat$Sampletype),]

zscore_cor_dat<-reshape(cor_dat[,c("zscore","sample","chr")],idvar=c("chr"),timevar="sample",direction="wide")

names(zscore_cor_dat)<-gsub("zscore[.]","",names(zscore_cor_dat))

row.names(zscore_cor_dat)<-zscore_cor_dat$chr

zscore_cor_dat$chr<-NULL

#cor_matrix<-cor(zscore_cor_dat)

library(Hmisc)

out<-rcorr(as.matrix(zscore_cor_dat), type = c("pearson")) #or spearman

#output:

#r : the correlation matrix

#n : the matrix of the number of observations used in analyzing each pair of variables

#P : the p-values corresponding to the significance levels of correlations.

pearson_pvals_cor<-out$P

pearson_cor_values<-out$r

cor_metadata<-metadata[metadata$ProcessingID %in% colnames(pearson_cor_values),]

cor_metadata$ProcessingID<-factor(cor_metadata$ProcessingID,levels=colnames(pearson_cor_values))

cor_metadata<-cor_metadata[order(cor_metadata$ProcessingID),]

col<- colorRampPalette(c("black", "white", "red"))(20)

```

WE-TE correlations

```{r WETEcors}

#embryo IDs from TE-WE pairs

#TE_WE_pairs_Embryo_IDs<-c("E50","E22", "E12","E13","E48", "E32", "E39", "E2", "E5")

TE_WE_pairs_Embryo_IDs<-c("E2","E4", "E5","E8","E9", "E10", "E14", "E15", "E17", "E24", "E33", "E35", "E38", "E39")

TE_WE_samplenames<-cor_metadata[cor_metadata$PaperID %in% TE_WE_pairs_Embryo_IDs, "ProcessingID"]

TE_WE_cor_vals<-pearson_cor_values[colnames(pearson_cor_values) %in% TE_WE_samplenames,colnames(pearson_cor_values) %in% TE_WE_samplenames]

#heatmap(x = TE_WE_cor_vals, col = col)

#heatmap(x = TE_WE_cor_vals, col = col, labRow=cor_metadata$Sampletype,labCol=cor_metadata$Sampletype)

cor_metadata$Sampletype<-gsub("wholeembryo","WE",cor_metadata$Sampletype)

cor_metadata$Sampletype<-gsub("trophectodermbiopsy","TEBx",cor_metadata$Sampletype)

cor_metadata$Embryo_Sampletype<-paste(cor_metadata$PaperID,cor_metadata$Sampletype,sep="_")

```

## How do WE-TE pairs compare to random WE-TE?

```{r WE-TEcors_vs_other}

cor_to_melt<-pearson_cor_values

cor_to_melt[lower.tri(cor_to_melt)]<-NA

cor.melted<-melt(cor_to_melt,na.rm=TRUE)

#rm self-cors

cor.melted<-cor.melted[which(cor.melted$Var1!=cor.melted$Var2),]

FROMSAMPLE<-cor_metadata[,c("ProcessingID","Sampletype","PaperID")]

#sampletype-1, #sampletype-2 , embryo 1, embryo 2

cormelted_annot<-merge(cor.melted,FROMSAMPLE,by.x="Var1",by.y="ProcessingID")

colnames(cormelted_annot)<-gsub("Sampletype","SampletypeVar1",colnames(cormelted_annot))

colnames(cormelted_annot)<-gsub("PaperID","PaperIDVar1",colnames(cormelted_annot))

cormelted_both_annot<-merge(cormelted_annot,FROMSAMPLE,by.x="Var2",by.y="ProcessingID")

names(cormelted_both_annot)[6:7]<-c("SampletypeVar2","PaperIDVar2")

#compare values from TE-WE pair to TE-WE not pair

#TE-WE pair

TEWEcorpair<-cormelted_both_annot[which(cormelted_both_annot$PaperIDVar1==cormelted_both_annot$PaperIDVar2),]

TEWEcorpair$pair<-"pair"

TEWEcor_notpair<-cormelted_both_annot[which(cormelted_both_annot$PaperIDVar1!=cormelted_both_annot$PaperIDVar2 & cormelted_both_annot$SampletypeVar1!=cormelted_both_annot$SampletypeVar2),]

TEWEcor_notpair$pair<-"notpair"

WETEcoranalysis<-rbind(TEWEcorpair,TEWEcor_notpair)

#ggplot(WETEcoranalysis,aes(value,fill=pair))+geom_density()+theme_bw()

pdf("cor_of_pairedorunpaired_TEWE_zscores.pdf")

ggplot(WETEcoranalysis,aes(pair,value,fill=pair))+geom_boxplot()+theme_bw()+geom_point(data=TEWEcorpair,aes(pair,value))+scale_fill_manual(values=c("pair"="red","notpair"="black"))

dev.off()

#ggplot(TEWEcor_notpair,aes(value))+geom_density()+theme_bw()

wilcox.test(TEWEcorpair$value,TEWEcor_notpair$value) #approaches significance, need more pairs

#p-value = 0.01553

wilcox.test(TEWEcorpair$value,TEWEcor_notpair$value,alternative="g") #p-value = 0.007766

```

# WE-TE pairs

- obvious caveat here is biopsy quality and noise in super small input...

- could do WE-PGS, but this has mosaicism caveat

- point of paper is to say TE biopsy maybe useful for informing on WE (*MAY* be useful) so do WE-TE agreement

- what metric to use? sigpotential agreement? distance from 0 threshold for each sampletype? difference between the two zscores regardless of position? <- i think difference of the zscores is best metric of 'agreement' a priori...

- correlation of zscore profiles...

```{r WE-TE-allchrs}

dat<-dat_save

dat<-dat[grep("whole|troph",dat$Sampletype),]

dat$WEChrCall<-"contained"

dat$WEChrCall[which(dat$zscore>2)]<-"gain"

dat$WEChrCall[which(dat$zscore<(-2))]<-"loss"

dat$sigpotential<-"no"

dat$sigpotential[which(dat$zscore>2 | dat$zscore<(-2))]<-"yes"

#break into matching TE and WE

TE_WE_pairs<-reshape(dat[,c("zscore","PaperID","Sampletype","chr")],idvar=c("PaperID","chr"),timevar="Sampletype",direction="wide")

samples_to_remove<-TE_WE_pairs$PaperID[which(is.na(TE_WE_pairs$zscore.trophectodermbiopsy)|is.na(TE_WE_pairs$zscore.wholeembryo))]

TE_WE_pairs2<-TE_WE_pairs[which(!(TE_WE_pairs$PaperID %in% samples_to_remove)),]

TE_WE_pairs<-TE_WE_pairs2

TE_WE_pairs$WEChrCall<-"euploid"

TE_WE_pairs$WEChrCall[which(TE_WE_pairs$zscore.wholeembryo>2)]<-"aneuploid"

TE_WE_pairs$WEChrCall[which(TE_WE_pairs$zscore.wholeembryo<(-2))]<-"aneuploid"

TE_WE_pairs_toplot<-dat[which(dat$PaperID %in% TE_WE_pairs$PaperID),]

TE_WE_pairs_toplot$chrnum<-factor(TE_WE_pairs_toplot$chrnum,levels=TE_WE_pairs_toplot$chrnum[order(TE_WE_pairs_toplot$chrnum)])

pdf("TE-WE_karyotype_agreement.pdf")

#ggplot(TE_WE_pairs_toplot,aes(chr,zscore,colour=sigpotential,shape=Sampletype))+geom_point()+theme_bw()+facet_wrap(~PaperID)+theme(axis.text.x = element_text(angle = 65, hjust = 1))+ggtitle("WE-TE pairs")+scale_colour_manual(values=c("no"="black","yes"="red"))

ggplot(TE_WE_pairs_toplot,aes(chrnum,zscore,colour=Sampletype,shape=sigpotential,group=chrnum))+geom_point(size=2)+theme_bw()+facet_wrap(~PaperID)+theme(axis.text.x = element_text(angle = 90, hjust = 1))+geom_line(color="black")+ggtitle("WE-TE pairs")+scale_color_manual(values=c("day3"="light blue","trophectodermbiopsy"="grey","wholeembryo"="black"))

dev.off()

```

Distribution of WE-TE zscore differences

```{r WETEzscorediffs}

TE_WE_pairs$zmax<-pmax(TE_WE_pairs$zscore.wholeembryo,TE_WE_pairs$zscore.trophectodermbiopsy)

TE_WE_pairs$zmin<-pmin(TE_WE_pairs$zscore.wholeembryo,TE_WE_pairs$zscore.trophectodermbiopsy)

TE_WE_pairs$diff<-TE_WE_pairs$zmax-TE_WE_pairs$zmin

TE_WE_pairs$E_chr<-paste(TE_WE_pairs$PaperID,TE_WE_pairs$chr,sep="_")

example_diff<-TE_WE_pairs[grep("E8_chr17",TE_WE_pairs$E_chr),]

pdf("DiffDist_E8chr17.pdf")

ggplot(TE_WE_pairs,aes(diff))+geom_density()+theme_bw()+geom_vline(aes(xintercept=example_diff$diff),color="red")+geom_text(aes(x=example_diff$diff,y=.3,label=example_diff$E_chr))+ggtitle("E8 chr17")

dev.off()

example_diff<-TE_WE_pairs[grep("E35_chr4",TE_WE_pairs$E_chr),]

pdf("DiffDist_E35chr4.pdf")

ggplot(TE_WE_pairs,aes(diff))+geom_density()+theme_bw()+geom_vline(aes(xintercept=example_diff$diff),color="red")+geom_text(aes(x=example_diff$diff,y=.2,label=example_diff$E_chr))+ggtitle("E35 chr4")

dev.off()

```

---

title: "RevisedFig4_Competence_karyotype"

output: html_notebook

---

Competence-associated differential gene expression analyses:

(1) RNA-inferred karyotype

(2) morphology

(3) morphokinetic

# Setup for RNA-karyotype

```{r setup}

library(DESeq2)

library(tximport)

library(readr)

library(stringr)

library(ggplot2)

library(reshape2)

library(plyr)

library(dplyr)

#rsem_count_dir<-"/Volumes/valor2/users/agroff/seq/humanEmbryo/quants/rsem/50bptrim"

rsem_count_dir<-"~/Dropbox/Rinnlab/manuscripts/EmbryoPaper2018/Review1/support_for_code/rsem_files/"

#make transcript to gene conversion file using dat1 from above

files <- list.files(rsem_count_dir,pattern="*.genes.results",full.names=TRUE)

filenames<-list.files(rsem_count_dir,pattern="*.genes.results")

filenames<-gsub(".genes.results","",filenames)

names(files) <- filenames

metadata<-read.table("/Users/abigailgroff/Dropbox/Rinnlab/manuscripts/EmbryoPaper2018/Review1/support_for_code/032018_WE_ploidy_metadata.txt",header=TRUE,stringsAsFactors = FALSE)

txn_chr_info<-read.table("~/Dropbox/PageLab/Projects/SexDiffs/supporting/txn_chr_info.tab",header=TRUE)

chr_sizes<-read.table("~/Dropbox/PageLab/Projects/SexDiffs/supporting/chrom_sizes.tab",header=TRUE)

tx2gene<-read.table("/Volumes/solexa_page/agroff/seq/humanEmbryo/analysis_old/analysis/tx2gene.tab",header=TRUE)

#annot_short<-read.table("~/Dropbox/Rinnlab/manuscripts/EmbryoPaper2018/Review1/FigsSup/embryo_transcript_annotation_gencodev19_hg19.tab")

potentially_tetraploid<-c("NRAG41","NRAG78","NRAG82","NRAG90","NRAG93","NRAG95","NRAG66")

metadata<-metadata[!metadata$ProcessingID%in%potentially_tetraploid,]

```

# Define and load samples

```{r defineeuploidaneuploid}

metadata<-metadata[which(metadata$Sampletype=="wholeembryo"),]

diff_files<-files[which(names(files)%in%metadata$ProcessingID)]

diff_metadata<-metadata[which(metadata$ProcessingID%in%names(diff_files)),]

txi <- tximport(diff_files, type="rsem", tx2gene=tx2gene)

rownames(diff_metadata)<-diff_metadata$ProcessingID

diff_metadata$RNAPloidy<-factor(diff_metadata$RNAPloidy)

txi$length<-txi$length+1

diff_metadata<-diff_metadata[order(diff_metadata$ProcessingID),]

#ddsTxi <- DESeqDataSetFromTximport(txi, colData=diff_metadata,design=~MorphoGradeCall+ImputedorKnownSex+MorphoGradeCall:ImputedorKnownSex)

#https://support.bioconductor.org/p/62355/

ddsTxi <- DESeqDataSetFromTximport(txi, colData=diff_metadata,design=~RNAPloidy)

```

```{r mito}

#COUNTS

#counttable<-as.data.frame(counts(ddsTxi))

#counttable$geneinfo<-row.names(counttable)

#counttable$txname<-str_split_fixed(counttable$geneinfo,"_",2)[,1]

#counttable$genename<-str_split_fixed(counttable$geneinfo,"_",2)[,2]

#counttable_chr<-merge(counttable,txn_chr_info,by.x="txname",by.y="TXNAME")

#mito<-counttable_chr[which(counttable_chr$chr=="chrM"),]

#mito.melt<-melt(mito)

#mito.melt.annot<-merge(mito.melt,diff_metadata,by.x="variable",by.y="ProcessingID")

#ggplot(mito.melt.annot,aes(RNAPloidy,value))+geom_boxplot(notch=TRUE)+geom_jitter()+facet_wrap(~Sampletype)+theme_bw()+facet_wrap(~genename, scales="free_y")

##ggplot(mito.melt.annot,aes(RNAPloidy,log(value)))+geom_boxplot(notch=TRUE)+geom_jitter()+facet_wrap(~Sampletype)+theme_bw()

##ggplot(mito.melt.annot,aes(RNAPloidy,log(value)))+geom_boxplot(notch=TRUE)+geom_jitter()+facet_wrap(~Sampletype)+theme_bw()+facet_wrap(~genename, scales="free_y")

#mito.melt.sum<-ddply(mito.melt[,c("variable","value")],.(variable),summarize,sampleSums=sum(value))

#mito.melt.sum.annot<-merge(mito.melt.sum,diff_metadata,by.x="variable",by.y="ProcessingID")

#ggplot(mito.melt.sum.annot,aes(RNAPloidy,sampleSums))+geom_boxplot(notch=TRUE)+geom_jitter()+facet_wrap(~Sampletype)+theme_bw()

##ggplot(mito.melt.sum.annot,aes(RNAPloidy,log(sampleSums)))+geom_boxplot(notch=TRUE)+geom_jitter()+facet_wrap(~Sampletype)+theme_bw()

##mito.melt.annot<-merge(mito.melt,diff_metadata,by.x="variable",by.y="ProcessingID")

##ggplot(mito.melt.annot,aes(PaperID,value,color=Sampletype))+geom_boxplot()#geom_jitter()

##ggplot(mito.melt.annot,aes(Geno,log(value)))+geom_boxplot(notch=TRUE)+facet_wrap(~Sampletype)

#TPMS

TPMs<-txi$abundance

mitogenesinfo<-tx2gene[tx2gene$TXNAME %in% mito$txname,]

mitogenesinfo$info<-paste(mitogenesinfo$TXNAME,mitogenesinfo$GENEID,sep="_")

mito_TPMs<-TPMs[mitogenesinfo$info,]

rnaploidy_metadata<-read.table("/Users/abigailgroff/Dropbox/Rinnlab/manuscripts/EmbryoPaper2018/Review1/support_for_code/032018_WE_ploidy_metadata.txt",header=TRUE,stringsAsFactors = FALSE)

paperIDmetadata<-read.table("~/Dropbox/Rinnlab/manuscripts/EmbryoPaper2018/Review1/FigsSup/metadata_021219.txt",header=TRUE,stringsAsFactors = FALSE)

metadata<-merge(rnaploidy_metadata[,c("RNAPloidy","ProcessingID","Sampletype")],paperIDmetadata[,c("ProcessingID","PaperID")],by.x="ProcessingID",by.y="ProcessingID")

mito.melt<-melt(mito_TPMs)

names(mito.melt)<-c("info","ProcessingID","TPM")

mito.melt$genename<-str_split_fixed(mito.melt$info,"_",2)[,2]

mito.melt.annot<-merge(mito.melt,metadata,by.x="ProcessingID",by.y="ProcessingID")

pdf("Embryo_mito_TPMs_byRNAploidy.pdf")

ggplot(mito.melt.annot,aes(RNAPloidy,TPM))+geom_boxplot(notch=TRUE)+geom_jitter()+facet_wrap(~Sampletype)+theme_bw()+facet_wrap(~genename, scales="free_y")

dev.off()

mito.melt.sum<-ddply(mito.melt[,c("ProcessingID","TPM")],.(ProcessingID),summarize,sampleSums=sum(value))

mito.melt.sum.annot<-merge(mito.melt.sum,metadata,by.x="variable",by.y="ProcessingID")

pdf("embryo_mito_sums_TPMs_byploidy.pdf")

ggplot(mito.melt.sum.annot,aes(RNAPloidy,sampleSums))+geom_boxplot(notch=TRUE)+geom_jitter()+theme_bw()

dev.off()

ggplot(mito.melt.sum.annot,aes(RNAPloidy,sampleSums,label=PaperID,color=Sampletype))+geom_boxplot(notch=TRUE)+geom_jitter()+theme_bw()+geom_text()

```

# RNAploidy Quality Filter

Remove genes that are not expressed in any sample

- can add binary screening so must be expressed >1 in any one sample

```{r quality_filter}

samples_keep<-read.table("~/Dropbox/Rinnlab/manuscripts/EmbryoPaper2018/Review1/rsem_files/March2018_samples_passing_QC.tab",header=TRUE)

#quality filtering

#remove genes with no counts

dds<-ddsTxi

nrow(dds)

dds <- dds[ rowSums(counts(dds)) > 1, ] #get rid of super noisy expression

nrow(dds)

#keep only remaining expressed genes (>=1TPM in at least 1 sample)

expressed_genes<-read.table("~/Dropbox/Rinnlab/manuscripts/EmbryoPaper2018/Review1/rsem_files/March2018_expressed_genes.tab")

expressed_genes<-expressed_genes$x

dds<-dds[row.names(dds)%in%expressed_genes,]

#remove mitochondrial reads here

counttable<-as.data.frame(counts(dds))

counttable$geneinfo<-row.names(counttable)

counttable$txname<-str_split_fixed(counttable$geneinfo,"_",2)[,1]

counttable$genename<-str_split_fixed(counttable$geneinfo,"_",2)[,2]

counttable_chr<-merge(counttable,txn_chr_info,by.x="txname",by.y="TXNAME")

mito<-counttable_chr[which(counttable_chr$chr=="chrM"),]

counttable<-counttable_chr[which(counttable_chr$chr!="chrM"),]

genomic_genes<-counttable$geneinfo

dds<-dds[row.names(dds)%in%genomic_genes,]

dim(dds)

dds<-dds[,(colnames(dds)%in%samples_keep$ProcessingID)] #get rid of low expressing samples

dim(dds)

#remove PAR1 genes

par1<-c("PLCXD1","GTPBP6","PPP2R3B","SHOX","CRLF2","CSF2RA","IL3RA","SLC25A6","ASMTL","P2RY8","CXYorf3","ASMT","DHRSXY","ZBED1","CD99","XG")

par1_genes<-counttable[which(counttable$genename %in% par1),"geneinfo"]

dds<-dds[!(row.names(dds)%in%par1_genes),]

dim(dds)

#update diff_files after QC

diff_files<-diff_files[names(diff_files)%in%dds@colData$ProcessingID]

```

PCA RNAploidy samples

```{r morphologicalPCA}

vsd<-varianceStabilizingTransformation(dds)

par(mfrow=c(1, 2))

dds <- estimateSizeFactors(dds)

plotPCA(vsd, intgroup = c("RNAPloidy"))+ggtitle("PCA, RNA ploidy")+theme_bw()

```

Differential expression RNAploidy grade

```{r DiffExp}

dds<-DESeq(dds)

resultsNames(dds)

res<-results(dds,alpha=0.05)

ressig<-as.data.frame(res[which(res$padj<0.05),])

ressig$info<-row.names(ressig)

ressig$genenames<-str_split_fixed(ressig$info,"_",2)[,2]

ressig$txname<-str_split_fixed(ressig$info,"_",2)[,1]

comb_dat<-as.data.frame(merge(ressig,annot_short,by.x="txname","txname"))

dat<-as.data.frame(res)

dat$sig<-"no"

dat$sig[which(dat$padj<0.05)]<-"yes"

dat$info<-row.names(dat)

dat$genenames<-str_split_fixed(dat$info,"_",2)[,2]

dat$txname<-str_split_fixed(dat$info,"_",2)[,1]

#write.table(dat,file="ploidy_diff.txt",sep="\t",quote=FALSE,row.names=FALSE,col.names=TRUE)

dat_orig<-read.table("~/Dropbox/Rinnlab/manuscripts/EmbryoPaper2018/Review1/ploidy_diff.txt",header=TRUE)

```

RNAploidy DE volcano

```{r ploiduDE}

pdf("ploidy_DE_volcano.pdf")

#ggplot(dat,aes(log2FoldChange,-log10(pvalue),colour=sig))+geom_point()+theme_bw()+scale_colour_manual(values=c("no"="black","yes"="red"))

ggplot(dat,aes(log2FoldChange,-log10(pvalue),label=genenames))+geom_bin2d()+theme_bw()+scale_fill_gradient("low"="light gray","high"="black")+geom_point(data=subset(dat,sig=="yes"),aes(log2FoldChange,-log10(pvalue)),color="red")#+geom_text(data=subset(dat,sig=="yes"),color="red")

dev.off()

```

ploidy DE TPM-based gene expression jitter plots

```{r TPM_jitters}

tpm_files<-lapply(diff_files,read.table,header=TRUE)

expression_info<-lapply(tpm_files,function(x){

newx<-x[,c("gene_id","TPM")]

newx

})

exp_info2<-lapply(seq_along(expression_info),function(x){

new<-expression_info[[x]]

new$samplename<-names(expression_info)[x]

as.data.frame(new)

})

expression_info<-do.call("rbind",exp_info2)

expression_info$genename<-str_split_fixed(expression_info$gene_id,"_",2)[,2]

expression_info<-expression_info[which(expression_info$genename %in% ressig$genenames),]

expression_annot<-merge(expression_info,metadata,by.x="samplename",by.y="ProcessingID")

library(data.table)

expression_annot_dt<-data.table(expression_annot)

expression_annot_dt$infocondition<-paste(expression_annot_dt$gene_id,expression_annot_dt$RNAploidy,sep="_")

ploidy_diff_genes<-unique(expression_annot_dt$genename)

#pdf("ploidy_diff_genes_D6_WE_jitter.pdf",height=30,width=20)

ggplot(expression_annot_dt,aes(RNAPloidy,TPM,colour=RNAPloidy))+geom_jitter()+theme_bw()+facet_wrap(~genename,scales="free")+geom_text(aes(label=ReadableEmbryoID))+scale_colour_manual(values=c("euploid"="Gray","aneuploid"="red"))

#dev.off()

pdf("RNAPloidy_diff_genes_WE_boxplot.pdf",height=30,width=20)

ggplot(expression_annot_dt,aes(RNAPloidy,TPM,colour=RNAPloidy))+geom_boxplot(notch=TRUE)+theme_bw()+facet_wrap(~genename,scales="free")+scale_colour_manual(values=c("euploid"="Gray","aneuploid"="red"))

dev.off()

```

**Expression of select genes in TE biopsies...**

Select all TE & WE samples for which we have TE&WE matched.

Read in TPMs for these files

```{r TE_biopsy_TPMs}

matched_TE_biopsies<-metadata[grep("TE&WE",metadata$avialabledata),]

matched_TE_biopsies<-matched_TE_biopsies[grep("day3",matched_TE_biopsies$Sampletype,invert=TRUE),]

#matched_TE_biopsies<-matched_TE_biopsies[grep("troph",matched_TE_biopsies$Sampletype),]

matched_TE_biopsies_files<-files[which(names(files)%in%matched_TE_biopsies$ProcessingID)]

tpm_files<-lapply(matched_TE_biopsies_files,read.table,header=TRUE)

expression_info_match<-lapply(tpm_files,function(x){

newx<-x[grep(paste(ressig$genenames,collapse="|"),x$gene_id),]

newx<-x[,c("gene_id","TPM")]

newx

})

exp_info2_match<-lapply(seq_along(expression_info_match),function(x){

new<-expression_info_match[[x]]

new$samplename<-names(expression_info_match)[x]

as.data.frame(new)

})

expression_info_match<-do.call("rbind",exp_info2_match)

expression_info_match$genename<-str_split_fixed(expression_info_match$gene_id,"_",2)[,2]

expression_info_match<-expression_info_match[which(expression_info_match$genename %in% morphokinetic_diff_genes),]

expression_annot_match<-merge(expression_info_match,metadata,by.x="samplename",by.y="ProcessingID")

library(data.table)

expression_annot_dt_match<-data.table(expression_annot_match)

expression_annot_dt_match$infocondition<-paste(expression_annot_dt_match$gene_id,expression_annot_dt_match$MorphokineticCall,sep="_")

expression_annot_dt_match$MorphokineticCall[is.na(expression_annot_dt_match$MorphokineticCall)]<-"NoCall"

expression_annot_dt_match$ReadableEmbryoID<-factor(expression_annot_dt_match$ReadableEmbryoID,levels=expression_annot_dt_match$ReadableEmbryoID[order(expression_annot_dt_match$MorphokineticCall,expression_annot_dt_match$ReadibleEmbryoNumber)])

ggplot(expression_annot_dt_match,aes(ReadableEmbryoID,log(TPM),colour=Sampletype,shape=MorphokineticCall))+theme_bw()+geom_point()+scale_colour_manual(values=c("trophectodermbiopsy"="gray","wholeembryo"="black"))+facet_wrap(~genename,scales="free")

ggplot(expression_annot_dt_match,aes(ReadableEmbryoID,TPM,colour=Sampletype,shape=MorphokineticCall))+theme_bw()+geom_point()+scale_colour_manual(values=c("trophectodermbiopsy"="gray","wholeembryo"="black"))+facet_wrap(~genename,scales="free")

expression_annot_dt_match$sampletype_call<-paste(expression_annot_dt_match$Sampletype,expression_annot_dt_match$MorphokineticCall,sep=" ")

expression_annot_dt_match$sampletype_call<-factor(expression_annot_dt_match$sampletype_call,levels=expression_annot_dt_match$sampletype_call[order(expression_annot_dt_match$MorphokineticCall,expression_annot_dt_match$Sampletype)])

#pdf("TE-WE-set_sig_morphokinetic_genes_boxplot.pdf",height=30,width=20)

ggplot(expression_annot_dt_match,aes(sampletype_call,TPM,colour=MorphokineticCall))+theme_bw()+facet_wrap(~genename,scales="free")+geom_boxplot()+scale_colour_manual(values=c("GOOD"="red","BAD"="black","NoCall"="light gray"))+theme(axis.text.x=element_text(angle=90, hjust=0))

#dev.off()

pdf("TE-WE-set_sig_morphokinetic_genes_jitter.pdf",height=30,width=20)

ggplot(expression_annot_dt_match,aes(sampletype_call,TPM,colour=MorphokineticCall,group=ReadableEmbryoID,shape=Sampletype))+theme_bw()+facet_wrap(~genename,scales="free")+geom_point()+geom_text(aes(label=ReadableEmbryoID))+geom_line()+scale_colour_manual(values=c("GOOD"="red","BAD"="black","NoCall"="light gray"))+theme(axis.text.x=element_text(angle=90, hjust=0))#+scale_colour_manual(values=c("trophectodermbiopsy"="gray","wholeembryo"="black"))

dev.off()

```

RNAPloidy DE GO TERMS

# GO term analysis of sig DE genes

```{r go_sig_genes, eval=FALSE, include=FALSE}

#source("https://bioconductor.org/biocLite.R")

#biocLite("org.Hs.eg.db")#,lib="/n/home06/agroff/R/x86_64-unknown-linux-gnu-library/3.3")

library(ReactomePA)

library(DOSE)

library(clusterProfiler)

require(biomaRt)

Down<-ressig[which(ressig$log2FoldChange<0),"genenames"]

Up<-ressig[which(ressig$log2FoldChange>0),"genenames"]

#ensembl <- useMart("ENSEMBL_MART_ENSEMBL","hsapiens_gene_ensembl",

# host="www.ensembl.org") # <- No longer works ...

ensembl = useMart(biomart="ENSEMBL_MART_ENSEMBL", host="grch37.ensembl.org", path="/biomart/martservice" ,dataset="hsapiens_gene_ensembl")

#https://support.bioconductor.org/p/62064/

#listAttributes(ensembl)

filters<-listFilters(ensembl)

#grep('gene',filters$name,value=TRUE, ignore.case=TRUE)

getEntrezIDs<-function (geneNames)

{

tmp <- getBM(attributes = c("entrezgene"), filters = "hgnc_symbol", values = geneNames, mart = ensembl)

tmp

}

require(org.Hs.eg.db)

doGO<-function(title,geneNames,ensembl){

#ensembl = useMart(biomart="ENSEMBL_MART_ENSEMBL", host="grch37.ensembl.org", path="/biomart/martservice" ,dataset="hsapiens_gene_ensembl")

#https://support.bioconductor.org/p/62064/

filters<-listFilters(ensembl)

sigEntrez<-getEntrezIDs(geneNames)

sigEZ<-strsplit(as.character(sigEntrez), ", ")

sigEZ<-unlist(sigEZ)

goBP<-enrichGO(gene=sigEZ,'org.Hs.eg.db',ont="BP",pvalueCutoff=0.05,readable=T)

goMF<-enrichGO(gene=sigEZ,'org.Hs.eg.db',ont="MF",pvalueCutoff=0.05,readable=T)

goCC<-enrichGO(gene=sigEZ,'org.Hs.eg.db',ont="CC",pvalueCutoff=0.05,readable=T)

makeGOplot<-function(goobj,catnum,p_cutoff){

res<-goobj@result

res<-res[which(res$p.adjust<p_cutoff),]

res<-res[order(res$p.adjust),]

res$Description<-factor(res$Description,levels=res$Description)

if(dim(res)[1]>catnum){res<-res[1:catnum,]}

ggplot(res,aes(Description,-log10(p.adjust)))+geom_bar(stat="identity")+theme_bw()+theme(axis.text.x=element_text(angle=-90, hjust=0))

#ggplot(res,aes(Description,Count))+geom_bar(stat="identity")+theme_bw()+theme(axis.text.x=element_text(angle=-90, hjust=0))

}

A<-makeGOplot(goBP,20,0.05)+ggtitle(paste("GO BP",title,sep = " "))

B<-makeGOplot(goMF,20,0.05)+ggtitle(paste("GO MF",title,sep = " "))

C<-makeGOplot(goCC,20,0.05)+ggtitle(paste("GO CC",title,sep = " "))

#write.table(goBP@result,file=paste(filename,"_goBP_result.tab",sep="\t"),quote=FALSE)

#write.table(goMF@result,paste(filename,"_goMF_result.tab",sep="\t"),quote=FALSE)

#write.table(goCC@result,paste(filename,"_goCC_result.tab",sep="\t"),quote=FALSE)

goBPres<-goBP@result

goMFres<-goMF@result

goCCres<-goCC@result

return(list(A,B,C,goBPres,goMFres,goCCres))

}

DOWN_outputList<-doGO("Down RNAploidy DE", Down,ensembl)

DOWN_outputList[[1]] #lots

DOWN_outputList[[2]] #structural constituent of ribosome

DOWN_outputList[[3]] #lots - ribosomes, endosomes, and mitochondria

#pdf("RNAPloidy_GO_BP_DE.pdf")

#DOWN_outputList[[1]]

#dev.off()

#pdf("RNAPloidy_GO_MF_DE.pdf")

#DOWN_outputList[[2]]

#dev.off()

#pdf("RNAPloidy_GO_CC_DE.pdf")

#DOWN_outputList[[3]]

#dev.off()

Down_goBP_result<-DOWN_outputList[[4]]

Down_goMF_result<-DOWN_outputList[[5]]

Down_goCC_result<-DOWN_outputList[[6]]

write.table(Down_goBP_result,file="DownGOBP_RNAPloidy.txt",sep="\t",quote=FALSE,col.names=TRUE)

write.table(Down_goMF_result,file="DownGOMF_RNAPloidy.txt",sep="\t",quote=FALSE,col.names=TRUE)

write.table(Down_goCC_result,file="DownGOCC_RNAPloidy.txt",sep="\t",quote=FALSE,col.names=TRUE)

#jitter plots of the genes contributing to this go enrichment

rm(genesofinterest)

genesofinterest<-unique(Down_goBP_result$geneID,Down_goCC_result$geneID)

genesofinterest_list<-unique(unlist(str_split(genesofinterest,"/")))

#genesofinterest_genes<-expression_annot_dt[grep(paste(genesofinterest_list,collapse="|"),expression_annot_dt$genename),]

genesofinterest_genes<-expression_annot_dt[expression_annot_dt$genename %in% genesofinterest_list,]

pdf("GOBP_andCC_RNAPloidy_jittersTPM.pdf")

ggplot(genesofinterest_genes,aes(RNAPloidy,TPM,colour=RNAPloidy))+geom_jitter()+theme_bw()+facet_wrap(~genename,scales="free")+scale_colour_manual(values=c("euploid"="Gray","aneuploid"="red"))#+geom_text(aes(label=ReadableEmbryoID))

#ggplot(genesofinterest_genes,aes(RNAPloidy,TPM,colour=RNAPloidy))+geom_boxplot(notch=TRUE)+theme_bw()+facet_wrap(~genename,scales="free")

dev.off()

pdf("GOBP_andCC_RNAPloidy_boxplotTPM.pdf")

ggplot(genesofinterest_genes,aes(RNAPloidy,TPM,colour=RNAPloidy))+geom_boxplot(notch=TRUE)+theme_bw()+facet_wrap(~genename,scales="free")+scale_colour_manual(values=c("euploid"="Gray","aneuploid"="red"))

dev.off()

ggplot(genesofinterest_genes,aes(RNAPloidy,TPM,colour=RNAPloidy))+geom_jitter()+theme_bw()+facet_wrap(~genename,scales="free")+scale_colour_manual(values=c("euploid"="Gray","aneuploid"="red"))

ggplot(genesofinterest_genes,aes(RNAPloidy,log(TPM),colour=RNAPloidy))+geom_jitter()+theme_bw()+facet_wrap(~genename,scales="free")+scale_colour_manual(values=c("euploid"="Gray","aneuploid"="red"))

ggplot(genesofinterest_genes,aes(RNAPloidy,TPM,colour=RNAPloidy))+geom_boxplot(notch=TRUE)+theme_bw()+facet_wrap(~genename,scales="free")+scale_colour_manual(values=c("euploid"="Gray","aneuploid"="red"))

ggplot(genesofinterest_genes,aes(RNAPloidy,log(TPM),colour=RNAPloidy))+geom_boxplot(notch=TRUE)+theme_bw()+facet_wrap(~genename,scales="free")+scale_colour_manual(values=c("euploid"="Gray","aneuploid"="red"))

UP_outputList<-doGO("UP RNAPloidy DE", Up,ensembl)

UP_outputList[[1]]#none

UP_outputList[[2]]#damage dna binding/etc

UP_outputList[[3]]#proteasome/peptidase etc

UP_goMF_result<-UP_outputList[[5]] #RAD23A

UP_goCC_result<-UP_outputList[[6]] #RAD23A and LY6E

write.table(UP_goMF_result,file="UPGOMF_RNAPloidy.txt",sep="\t",quote=FALSE,col.names=TRUE)

write.table(UP_goCC_result,file="UPGOCC_RNAPloidy.txt",sep="\t",quote=FALSE,col.names=TRUE)

#jitter plots of the genes contributing to this go enrichment

rm(genesofinterest)

genesofinterest<-c("RAD23A","LY6E")

genesofinterest_list<-unique(unlist(str_split(genesofinterest,"/")))

#genesofinterest_genes<-expression_annot_dt[grep(paste(genesofinterest_list,collapse="|"),expression_annot_dt$genename),]

genesofinterest_genes<-expression_annot_dt[expression_annot_dt$genename %in% genesofinterest_list,]

ggplot(genesofinterest_genes,aes(RNAPloidy,TPM,colour=RNAPloidy))+geom_boxplot(notch=TRUE)+theme_bw()+facet_wrap(~genename,scales="free")+scale_colour_manual(values=c("euploid"="Gray","aneuploid"="red"))

detach("package:biomaRt")

```

RNAPloidy GSEA

##GSEA setup

```{r gsea_diffs, include=FALSE}

library(GSA)

library(limma)

library(gplots)

library(marray)

library(RColorBrewer)

library(RMySQL)

reactome_gs <- GSA.read.gmt("/Volumes/valor2/users/agroff/GSEA/c2.cp.reactome.v4.0.symbols.gmt")

biocarta_gs <- GSA.read.gmt("/Volumes/valor2/users/agroff/GSEA/c2.cp.biocarta.v4.0.symbols.gmt")

res<-as.data.frame(res)

geneinfo<-row.names(res)

res$info<-geneinfo

geneinfo2<-str_split_fixed(geneinfo,"_",2)

res$txname<-geneinfo2[,1]

res$genename<-geneinfo2[,2]

dat<-merge(res,txn_chr_info,by.x="txname","TXNAME")

dat<-dat[order(dat$stat),]

gene_set_index <- function(genelist, short_names){

which(short_names %in% genelist)

}

get_gene_set_p_vals <- function(input, gs, alternative){

gene_set_indices <- lapply(gs$genesets, function(genelist){

gene_set_index(genelist,input$short_name)

})

pvl<-lapply(gene_set_indices,geneSetTest,input$test_stat, alternative=alternative)

pvl_mat<-as.data.frame(t(unlist(pvl)))

colnames(pvl_mat) <- gs$geneset.names

return(pvl_mat)

}

get_gene_set_q_vals <- function(pvl_mat, method="bonferroni"){

comp_corrected <- matrix(p.adjust(pvl_mat, method=method), nrow=nrow(pvl_mat), ncol=ncol(pvl_mat))

colnames(comp_corrected) <- colnames(pvl_mat)

rownames(comp_corrected) <- rownames(pvl_mat)

return(comp_corrected)

}

colMins<-function(x){

apply(x,2,min)

}

rowMins<-function(x){

apply(x,1,min)

}

InputCols<-maPalette(low="white",high="red",k=100)

ztest<-function(samp,pop){

(mean(samp,na.rm=T)-mean(pop,na.rm=T))/sd(pop,na.rm=T)

}

get_gene_set_ztest <- function(scoring_df, gs){

gene_set_indices <- lapply(gs$genesets, function(genelist){

gene_set_index(genelist, scoring_df$short_name)

})

zscores <- lapply(gene_set_indices,function(gsi){

ztest(scoring_df$test_stat[gsi],scoring_df$test_stat)

})

zscore_mat<-do.call(rbind,lapply(zscores,unlist))

rownames(zscore_mat) <- gs$geneset.names

colnames(zscore_mat) <- "zscore"

return(zscore_mat)

}

```

## GSEA computation

```{r gsea, include=FALSE}

df.pop<-data.frame("short_name"=toupper(dat$GENEID),"test_stat"=dat$stat)

df.pop.unique<-unique(df.pop)

rownames(df.pop.unique)<-NULL

df.pop.unique.ordered<-df.pop.unique[order(df.pop.unique$test_stat),]

Input.df<-df.pop.unique.ordered

Input.df$short_name<-as.character(Input.df$short_name)

gseaInput.df<-Input.df

gseaInput.df$test_stat[which(is.na(gseaInput.df$test_stat))]<-0

reactome_pvl_mat <- get_gene_set_p_vals(Input.df, reactome_gs,alternative="either")

reactome_pvl_corrected <- get_gene_set_q_vals(reactome_pvl_mat)

reactome_pvl_corrected<-rbind(reactome_pvl_corrected,reactome_pvl_corrected)

biocarta_pvl_mat <- get_gene_set_p_vals(Input.df, biocarta_gs, alternative="either")

biocarta_pvl_corrected <- get_gene_set_q_vals(biocarta_pvl_mat)

biocarta_pvl_corrected<-rbind(biocarta_pvl_corrected,biocarta_pvl_corrected)

reactome_zscores<-get_gene_set_ztest(Input.df,reactome_gs)

reactome_zscores<-cbind(reactome_zscores,reactome_zscores)

biocarta_zscores<-get_gene_set_ztest(Input.df,biocarta_gs)

biocarta_zscores<-cbind(biocarta_zscores,biocarta_zscores)

k <- 100

myColors<-maPalette(low="blue",mid="white",high="red",k=k)

myBreaks<-seq(-2,2,length.out=(k+1))

enrichmentBreaks<-seq(0,6,length.out=(k+1))

```

## GSEA figures

Biocarta zscore: BIOCARTA_PROTEASOME_PATHWAY - down (in euploid i think)

```{r gsea_zscore_biocarta, fig.height=8, fig.width=8}

x<-((biocarta_zscores[which(colMins(biocarta_pvl_corrected) < 0.01),]))

x_ordered<-x[order(x[,1], decreasing=TRUE),]

if(length(x_ordered)>50){x_ordered<-x_ordered[1:50,]}

x_ordered<-as.matrix(x_ordered)

if(dim(x_ordered)[1]>1){

heatmap.2(x_ordered, trace="none",col=myColors,breaks=myBreaks,margins=c(1,20),dendrogram="both",labCol=c(""),cexRow =1, offsetRow=0)

}else{print("Not enough significant categories to print a heatmap!")}

rm(x_ordered)

rm(x)

```

Reactome zscore:

```{r gsea_zscore_reactome, fig.height=8, fig.width=8}

x<-((reactome_zscores[which(colMins(reactome_pvl_corrected) < 0.01),]))

x_ordered<-x[order(x[,1], decreasing=TRUE),]

if(length(x_ordered)>50){x_ordered<-x_ordered[1:50,]}

x_ordered<-as.matrix(x_ordered)

if(dim(x_ordered)[1]>1){

heatmap.2(x_ordered, trace="none",col=myColors,breaks=myBreaks,margins=c(1,20),dendrogram="both",labCol=c(""),cexRow =1, offsetRow=0)

}else{print("Not enough significant categories to print a heatmap!")}

rm(x_ordered)

rm(x)

x<-((reactome_zscores[which(colMins(reactome_pvl_corrected) < 0.01),]))

x_ordered<-x[order(x[,1], decreasing=TRUE),]

if(length(x_ordered)>50){x_ordered<-x_ordered[1:50,]}

x_ordered<-as.matrix(x_ordered)

#pdf("Reactome_GSEA_RNAploidy_zscore.pdf")

heatmap.2(x_ordered, trace="none",col=myColors,breaks=myBreaks,margins=c(1,20),dendrogram="both",labCol=c(""),cexRow =1, offsetRow=0)

#dev.off()

rm(x_ordered)

rm(x)

```

Replicate scatters:

```{r repscatters}

tpm_files<-lapply(diff_files,read.table,header=TRUE)

expression_info<-lapply(tpm_files,function(x){

newx<-x[,c("gene_id","TPM")]

newx

})

exp_info2<-lapply(seq_along(expression_info),function(x){

new<-expression_info[[x]]

new$samplename<-names(expression_info)[x]

as.data.frame(new)

})

expression_info<-do.call("rbind",exp_info2)

expression_info$genename<-str_split_fixed(expression_info$gene_id,"_",2)[,2]

exp_info_wide<-dcast(expression_info,gene_id+genename~samplename,value.var="TPM")

library(GGally)

ggpairs(exp_info_wide[,3:8]) #currently only 5 genes--need to read in ALL!

```

---

title: " mito anlysis"

output: html_notebook

---

# Setup for review mito anlysis

```{r setup}

library(DESeq2)

library(tximport)

library(readr)

library(stringr)

library(ggplot2)

library(reshape2)

library(plyr)

library(dplyr)

#rsem_count_dir<-"/Volumes/valor2/users/agroff/seq/humanEmbryo/quants/rsem/50bptrim"

rsem_count_dir<-"~/Dropbox/Rinnlab/manuscripts/EmbryoPaper2018/Review1/support_for_code/rsem_files/"

#make transcript to gene conversion file using dat1 from above

files <- list.files(rsem_count_dir,pattern="*.genes.results",full.names=TRUE)

filenames<-list.files(rsem_count_dir,pattern="*.genes.results")

filenames<-gsub(".genes.results","",filenames)

names(files) <- filenames

metadata<-read.table("/Users/abigailgroff/Dropbox/Rinnlab/manuscripts/EmbryoPaper2018/Review1/support_for_code/032018_WE_ploidy_metadata.txt",header=TRUE,stringsAsFactors = FALSE)

txn_chr_info<-read.table("~/Dropbox/PageLab/Projects/SexDiffs/supporting/txn_chr_info.tab",header=TRUE)

chr_sizes<-read.table("~/Dropbox/PageLab/Projects/SexDiffs/supporting/chrom_sizes.tab",header=TRUE)

tx2gene<-read.table("/Volumes/solexa_page/agroff/seq/humanEmbryo/analysis_old/analysis/tx2gene.tab",header=TRUE)

#annot_short<-read.table("~/Dropbox/Rinnlab/manuscripts/EmbryoPaper2018/Review1/FigsSup/embryo_transcript_annotation_gencodev19_hg19.tab")

potentially_tetraploid<-c("NRAG41","NRAG78","NRAG82","NRAG90","NRAG93","NRAG95","NRAG66")

metadata<-metadata[!metadata$ProcessingID%in%potentially_tetraploid,]

```

# Define and load samples

```{r defineeuploidaneuploid}

metadata<-metadata[grep("wholeembryo|troph",metadata$Sampletype),]

diff_files<-files[which(names(files)%in%metadata$ProcessingID)]

diff_metadata<-metadata[which(metadata$ProcessingID%in%names(diff_files)),]

txi <- tximport(diff_files, type="rsem", tx2gene=tx2gene)

rownames(diff_metadata)<-diff_metadata$ProcessingID

txi$length<-txi$length+1

```

```{r mito}

#COUNTS

#counttable<-as.data.frame(counts(ddsTxi))

#counttable$geneinfo<-row.names(counttable)

#counttable$txname<-str_split_fixed(counttable$geneinfo,"_",2)[,1]

#counttable$genename<-str_split_fixed(counttable$geneinfo,"_",2)[,2]

#counttable_chr<-merge(counttable,txn_chr_info,by.x="txname",by.y="TXNAME")

#mito<-counttable_chr[which(counttable_chr$chr=="chrM"),]

#mito.melt<-melt(mito)

#mito.melt.annot<-merge(mito.melt,diff_metadata,by.x="variable",by.y="ProcessingID")

#ggplot(mito.melt.annot,aes(RNAPloidy,value))+geom_boxplot(notch=TRUE)+geom_jitter()+facet_wrap(~Sampletype)+theme_bw()+facet_wrap(~genename, scales="free_y")

##ggplot(mito.melt.annot,aes(RNAPloidy,log(value)))+geom_boxplot(notch=TRUE)+geom_jitter()+facet_wrap(~Sampletype)+theme_bw()

##ggplot(mito.melt.annot,aes(RNAPloidy,log(value)))+geom_boxplot(notch=TRUE)+geom_jitter()+facet_wrap(~Sampletype)+theme_bw()+facet_wrap(~genename, scales="free_y")

#mito.melt.sum<-ddply(mito.melt[,c("variable","value")],.(variable),summarize,sampleSums=sum(value))

#mito.melt.sum.annot<-merge(mito.melt.sum,diff_metadata,by.x="variable",by.y="ProcessingID")

#ggplot(mito.melt.sum.annot,aes(RNAPloidy,sampleSums))+geom_boxplot(notch=TRUE)+geom_jitter()+facet_wrap(~Sampletype)+theme_bw()

##ggplot(mito.melt.sum.annot,aes(RNAPloidy,log(sampleSums)))+geom_boxplot(notch=TRUE)+geom_jitter()+facet_wrap(~Sampletype)+theme_bw()

##mito.melt.annot<-merge(mito.melt,diff_metadata,by.x="variable",by.y="ProcessingID")

##ggplot(mito.melt.annot,aes(PaperID,value,color=Sampletype))+geom_boxplot()#geom_jitter()

##ggplot(mito.melt.annot,aes(Geno,log(value)))+geom_boxplot(notch=TRUE)+facet_wrap(~Sampletype)

#TPMS

TPMs<-txi$abundance

mitogenesinfo<-txn_chr_info[grep("M",txn_chr_info$chr),]

mitogenesinfo$info<-paste(mitogenesinfo$TXNAME,mitogenesinfo$GENEID,sep="_")

mito_TPMs<-TPMs[mitogenesinfo$info,]

rnaploidy_metadata<-read.table("/Users/abigailgroff/Dropbox/Rinnlab/manuscripts/EmbryoPaper2018/Review1/support_for_code/032018_WE_ploidy_metadata.txt",header=TRUE,stringsAsFactors = FALSE)

paperIDmetadata<-read.table("~/Dropbox/Rinnlab/manuscripts/EmbryoPaper2018/Review1/FigsSup/metadata_021219.txt",header=TRUE,stringsAsFactors = FALSE)

metadata<-merge(rnaploidy_metadata[,c("RNAPloidy","ProcessingID","Sampletype","MorphologicalGrade","MorphoGradeCall","MorphokineticCall")],paperIDmetadata[,c("ProcessingID","PaperID")],by.x="ProcessingID",by.y="ProcessingID")

mito.melt<-melt(mito_TPMs)

names(mito.melt)<-c("info","ProcessingID","TPM")

mito.melt$genename<-str_split_fixed(mito.melt$info,"_",2)[,2]

mito.melt.annot<-merge(mito.melt,metadata,by.x="ProcessingID",by.y="ProcessingID")

pdf("Embryo_mito_TPMs_byRNAploidy.pdf")

ggplot(mito.melt.annot,aes(RNAPloidy,TPM))+geom_boxplot(notch=FALSE)+geom_jitter()+theme_bw()+facet_wrap(~genename+Sampletype, scales="free_y")

dev.off()

mito.melt.sum<-ddply(mito.melt[,c("ProcessingID","TPM")],.(ProcessingID),summarize,sampleSums=sum(TPM))

mito.melt.sum.annot<-merge(mito.melt.sum,metadata,by.x="ProcessingID",by.y="ProcessingID")

pdf("embryo_mito_sums_TPMs_byploidy.pdf")

ggplot(mito.melt.sum.annot,aes(RNAPloidy,sampleSums,label=PaperID))+geom_boxplot(notch=TRUE)+geom_jitter()+theme_bw()+facet_wrap(~Sampletype, scales="free_y")+geom_text(color="red")

ggplot(mito.melt.sum.annot,aes(RNAPloidy,sampleSums))+geom_boxplot(notch=TRUE)+geom_jitter()+theme_bw()+facet_wrap(~Sampletype, scales="free_y")

dev.off()

#WHICH OF THESE ARE SIGNIFICANT? (T-TESTS)

mito_split<-split(mito.melt.annot,mito.melt.annot$Sampletype)

mito_WE<-mito_split[["wholeembryo"]]

mito_TE<-mito_split[["trophectodermbiopsy"]]

mito_WE_genelist<-split(mito_WE,mito_WE$genename)

WE_ttest_res<-lapply(mito_WE_genelist,function(x) t.test(subset(x,RNAPloidy=="euploid","TPM"),subset(x,RNAPloidy=="aneuploid","TPM"),alternative="greater"))

#WE_ttest_res<-lapply(mito_WE_genelist,function(x) t.test(subset(x,RNAPloidy=="euploid","TPM"),subset(x,RNAPloidy=="aneuploid","TPM")))

names(WE_ttest_res)[unlist(lapply(WE_ttest_res,function(x)x$p.value<0.01))]

#[1] "MT-ND5"

WE_ttest_res[["MT-ND5"]]

mito_TE_genelist<-split(mito_TE,mito_TE$genename)

TE_ttest_res<-lapply(mito_TE_genelist,function(x) t.test(subset(x,RNAPloidy=="euploid","TPM"),subset(x,RNAPloidy=="aneuploid","TPM"),alternative="greater"))

#TE_ttest_res<-lapply(mito_TE_genelist,function(x) t.test(subset(x,RNAPloidy=="euploid","TPM"),subset(x,RNAPloidy=="aneuploid","TPM")))

names(TE_ttest_res)[unlist(lapply(TE_ttest_res,function(x)x$p.value<0.01))]

#none

MTND5<-mito.melt.annot[grep("MT-ND5",mito.melt.annot$genename),]

MTND5_WE<-MTND5[grep("whole",MTND5$Sampletype),]

t.test(subset(MTND5_WE,RNAPloidy=="euploid","TPM"),subset(MTND5_WE,RNAPloidy=="aneuploid","TPM"))

t.test(subset(MTND5_WE,RNAPloidy=="euploid","TPM"),subset(MTND5_WE,RNAPloidy=="aneuploid","TPM"),alternative="greater")

MTND5_TE<-MTND5[grep("troph",MTND5$Sampletype),]

t.test(subset(MTND5_TE,RNAPloidy=="euploid","TPM"),subset(MTND5_TE,RNAPloidy=="aneuploid","TPM"))

t.test(subset(MTND5_TE,RNAPloidy=="euploid","TPM"),subset(MTND5_TE,RNAPloidy=="aneuploid","TPM"),alternative="greater")

MTATP8<-mito.melt.annot[grep("MT-ATP8",mito.melt.annot$genename),]

MTATP8_WE<-MTATP8[grep("whole",MTATP8$Sampletype),]

t.test(subset(MTATP8_WE,RNAPloidy=="euploid","TPM"),subset(MTATP8_WE,RNAPloidy=="aneuploid","TPM"))

t.test(subset(MTATP8_WE,RNAPloidy=="euploid","TPM"),subset(MTATP8_WE,RNAPloidy=="aneuploid","TPM"),alternative="greater")

MTATP8_TE<-MTATP8[grep("troph",MTATP8$Sampletype),]

t.test(subset(MTATP8_TE,RNAPloidy=="euploid","TPM"),subset(MTATP8_TE,RNAPloidy=="aneuploid","TPM"))

t.test(subset(MTATP8_TE,RNAPloidy=="euploid","TPM"),subset(MTATP8_TE,RNAPloidy=="aneuploid","TPM"),alternative="greater")

```

# Morphological

GOOD: (grade: AA)

BAD: (grade: CC)

3 good and 12 bad and wash5p. 10 sig genes. 5 up and 5 down.

# Setup

```{r knitropts}

knitr::opts_chunk$set(echo=FALSE, message=FALSE, warning=FALSE,fig.path = "/ploidy_images/", dev=c('png', 'pdf'))

```

```{r setup}

library(DESeq2)

library(tximport)

library(readr)

library(stringr)

library(ggplot2)

library(reshape2)

library(plyr)

library(dplyr)

#library(hexbin)

rsem_count_dir<-"/Volumes/valor2/users/agroff/seq/humanEmbryo/quants/rsem/50bptrim"

#make transcript to gene conversion file using dat1 from above

files <- list.files(rsem_count_dir,pattern="*.genes.results",full.names=TRUE)

filenames<-list.files(rsem_count_dir,pattern="*.genes.results")

filenames<-gsub(".genes.results","",filenames)

names(files) <- filenames

metadata<-read.table("/Volumes/valor2/users/agroff/seq/humanEmbryo/analysis/032018_WE_ploidy_metadata.txt",header=TRUE,stringsAsFactors = FALSE)

annot_short<-read.table("/Volumes/valor2/users/agroff/seq/humanEmbryo/analysis/embryo_transcript_annotation_gencodev19_hg19.tab")

tx2gene<-read.table("/Volumes/valor2/users/agroff/seq/humanEmbryo/analysis/tx2gene.tab",header=TRUE)

txn_chr_info<-read.table("/Volumes/valor2/users/agroff/seq/humanEmbryo/analysis/txn_chr_info.tab",header=TRUE)

chr_sizes<-read.table("/Volumes/valor2/users/agroff/seq/humanEmbryo/analysis/deseq_all_old/chrom_sizes.tab",header=TRUE)

potentially_tetraploid<-c("NRAG41","NRAG78","NRAG82","NRAG90","NRAG93","NRAG95","NRAG66")

metadata<-metadata[!metadata$ProcessingID%in%potentially_tetraploid,]

```

```{r defineMorpho}

morphosamples<-metadata[grep("Good|Bad",metadata$MorphoGradeCall),]

morphosamples<-morphosamples[grep("wholeembryo",morphosamples$Sampletype),]

morphosamples<-morphosamples[grep("AA|CC",morphosamples$MorphologicalGrade),]

diff_files<-files[which(names(files)%in%morphosamples$ProcessingID)]

diff_metadata<-metadata[which(metadata$ProcessingID%in%names(diff_files)),]

txi <- tximport(diff_files, type="rsem", tx2gene=tx2gene, reader=read_tsv)

rownames(diff_metadata)<-diff_metadata$ProcessingID

diff_metadata<-diff_metadata[order(diff_metadata$ProcessingID),]

diff_metadata$MorphoGradeCall<-factor(diff_metadata$MorphoGradeCall)

diff_metadata$EmbAgeatBxDay<-paste("Day", diff_metadata$EmbAgeatBx)

#add 1 to all lengths in txi

#https://support.bioconductor.org/p/92763/

txi$length<-txi$length+1

ddsTxi <- DESeqDataSetFromTximport(txi, colData=diff_metadata,design=~MorphoGradeCall)

```

# Morphology Quality Filter

Remove genes that are not expressed in any sample

- can add binary screening so must be expressed >10 in any one sample

```{r quality_filter}

expressed_genes<-read.table("/Volumes/valor2/users/agroff/seq/humanEmbryo/analysis/March2018_expressed_genes.tab",header=TRUE)

expressed_genes<-expressed_genes$x

samples_keep<-read.table("/Volumes/valor2/users/agroff/seq/humanEmbryo/analysis/March2018_samples_passing_QC.tab",header=TRUE)

#quality filtering

#remove genes with no counts

dds<-ddsTxi

nrow(dds)

dds <- dds[ rowSums(counts(dds)) > 1, ] #get rid of super noisy expression

nrow(dds)

dim(dds)

dds<-dds[,(colnames(dds)%in%samples_keep$ProcessingID)] #get rid of low expressing samples

dim(dds)

#keep only remaining expressed genes (>=1TPM in at least 1 sample)

dds<-dds[row.names(dds)%in%expressed_genes,]

#remove mitochondrial reads here

counttable<-as.data.frame(counts(dds))

counttable$geneinfo<-row.names(counttable)

counttable$txname<-str_split_fixed(counttable$geneinfo,"_",2)[,1]

counttable$genename<-str_split_fixed(counttable$geneinfo,"_",2)[,2]

counttable_chr<-merge(counttable,txn_chr_info,by.x="txname",by.y="TXNAME")

mito<-counttable_chr[which(counttable_chr$chr=="chrM"),]

counttable<-counttable_chr[which(counttable_chr$chr!="chrM"),]

genomic_genes<-counttable$geneinfo

dds<-dds[row.names(dds)%in%genomic_genes,]

#remove PAR1 genes

par1<-c("PLCXD1","GTPBP6","PPP2R3B","SHOX","CRLF2","CSF2RA","IL3RA","SLC25A6","ASMTL","P2RY8","CXYorf3","ASMT","DHRSXY","ZBED1","CD99","XG")

par1_genes<-counttable[which(counttable$genename %in% par1),"geneinfo"]

dds<-dds[!(row.names(dds)%in%par1_genes),]

dim(dds)

#update diff_files after QC

diff_files<-diff_files[names(diff_files)%in%dds@colData$ProcessingID]

```

PCA morpho samples

```{r morphologicalPCA}

vsd<-varianceStabilizingTransformation(dds)

par(mfrow=c(1, 2))

dds <- estimateSizeFactors(dds)

plotPCA(vsd, intgroup = c("MorphoGradeCall"))+ggtitle("PCA, MorphoGradeCall")+theme_bw()

```

Differential expression morphological grade

```{r DiffExp}

dds<-DESeq(dds)

resultsNames(dds)

#res<-results(dds,name="MorphoGradeCall_Good_vs_Bad",alpha=0.05)

res<-results(dds,alpha=0.05)

ressig<-as.data.frame(res[which(res$padj<0.05),])

ressig$info<-row.names(ressig)

ressig$genenames<-str_split_fixed(ressig$info,"_",2)[,2]

ressig$txname<-str_split_fixed(ressig$info,"_",2)[,1]

comb_dat<-as.data.frame(merge(ressig,annot_short,by.x="txname","txname"))

dat<-as.data.frame(res)

dat$sig<-"no"

dat$sig[which(dat$padj<0.05)]<-"yes"

dat$info<-row.names(dat)

dat$genenames<-str_split_fixed(dat$info,"_",2)[,2]

dat$txname<-str_split_fixed(dat$info,"_",2)[,1]

#write.table(dat,file="morphological_diff.txt",sep="\t",quote=FALSE,row.names=FALSE,col.names=TRUE)

```

Morphological DE volcano

```{r morphoDE}

pdf("morphological_DE_volcano_AAvCC.pdf")

#ggplot(dat,aes(log2FoldChange,-log10(pvalue),colour=sig,label=genenames))+geom_point()+theme_bw()+scale_colour_manual(values=c("no"="black","yes"="red"))+geom_text(data=subset(dat,dat$sig=="yes"))

ggplot(dat,aes(log2FoldChange,-log10(pvalue),label=genenames))+geom_bin2d()+theme_bw()+scale_fill_gradient("low"="light gray","high"="black")+geom_point(data=subset(dat,sig=="yes"),aes(log2FoldChange,-log10(pvalue)),color="red")#+geom_text(data=subset(dat,sig=="yes"),color="red")

dev.off()

```

Morphological DE TPM-based gene expression jitter plots

```{r TPM_jitters}

tpm_files<-lapply(diff_files,read.table,header=TRUE)

expression_info<-lapply(tpm_files,function(x){

newx<-x[,c("gene_id","TPM")]

newx

})

exp_info2<-lapply(seq_along(expression_info),function(x){

new<-expression_info[[x]]

new$samplename<-names(expression_info)[x]

as.data.frame(new)

})

expression_info<-do.call("rbind",exp_info2)

expression_info$genename<-str_split_fixed(expression_info$gene_id,"_",2)[,2]

all_dat<-expression_info

expression_info<-expression_info[which(expression_info$genename %in% ressig$genenames),]

expression_annot<-merge(expression_info,metadata,by.x="samplename",by.y="ProcessingID")

all_dat_annot<-merge(all_dat,metadata,by.x="samplename",by.y="ProcessingID")

library(data.table)

expression_annot_dt<-data.table(expression_annot)

expression_annot_dt$infocondition<-paste(expression_annot_dt$gene_id,expression_annot_dt$MorphoGradeCall,sep="_")

expression_annot_dt$TPM<-as.numeric(expression_annot_dt$TPM)

mean_by_condition<-aggregate(expression_annot_dt[,c("MorphoGradeCall","genename","TPM")],by=list(expression_annot_dt$MorphoGradeCall,expression_annot_dt$genename),mean)

mean_by_condition$MorphoGradeCall<-NULL

mean_by_condition$genename<-NULL

names(mean_by_condition)<-c("MorphoGradeCall","genename","MeanTPM")

mean_data<-reshape(mean_by_condition, idvar = "genename", timevar = "MorphoGradeCall", ids="MeanTPM",direction = "wide")

mean_data$log2foldchange_wrt_good<-log2(mean_data$MeanTPM.Good/mean_data$MeanTPM.Bad)

mean_data_binary<-mean_data

mean_data_binary<-mean_data_binary[,c("MeanTPM.Bad","MeanTPM.Good")]

mean_data_binary[mean_data_binary<10]<-0

mean_data_binary[mean_data_binary>=10]<-1

binary_sums<-rowSums(mean_data_binary) #keep 1 or 2, get rid of 0

mean_data_filtered<-mean_data[which(binary_sums>0),]

data_to_plot<-expression_annot_dt[grep(paste(expression_annot_dt$txid,collapse="|"), expression_annot_dt$infocondition),]

pdf("morphoAAvCC_siggenes_jitter.pdf")

ggplot(data_to_plot,aes(MorphoGradeCall,TPM,colour=MorphoGradeCall))+geom_jitter()+theme_bw()+facet_wrap(~genename,scales="free")+scale_colour_manual(values=c("Bad"="Red","Good"="Gray"))#+geom_text(aes(label=samplename))

dev.off()

pdf("morphoAAvCC_siggenes_boxplot.pdf")

ggplot(data_to_plot,aes(MorphoGradeCall,TPM,colour=MorphoGradeCall))+geom_boxplot(notch=TRUE)+theme_bw()+facet_wrap(~genename,scales="free")+scale_colour_manual(values=c("Bad"="Red","Good"="Gray"))

dev.off()

othergenes<-all_dat_annot[grep("SRF", all_dat_annot$genename),]

ggplot(othergenes,aes(MorphoGradeCall,TPM,colour=MorphoGradeCall))+geom_jitter()+theme_bw()+facet_wrap(~genename,scales="free")+scale_colour_manual(values=c("Bad"="Red","Good"="Gray"))#+geom_text(aes(label=samplename))

to_wide<-data_to_plot[,c("genename","samplename","TPM")]

all_dat_wide<-reshape(to_wide, idvar = "genename", timevar = "samplename", ids="TPM",direction = "wide")

names(all_dat_wide)<-gsub("TPM[.]","",names(all_dat_wide))

row.names(all_dat_wide)<-all_dat_wide$genename

all_dat_mat<-as.matrix(all_dat_wide[,2:(dim(all_dat_wide)[2])])

row.names(all_dat_mat)<-all_dat_wide$genename

library(gplots)

library(marray)

k <- 100

myColors<-maPalette(low="blue4",mid="white",high="brown",k=k)

heatmap.2(all_dat_mat,scale="row",col=myColors,trace="none",dendrogram="col")#,Rowv=FALSE)

```

Morphological DE GO TERMS

# GO term analysis of sig DE genes

```{r go_sig_genes, eval=FALSE, include=FALSE}

#source("https://bioconductor.org/biocLite.R")

#biocLite("org.Hs.eg.db")#,lib="/n/home06/agroff/R/x86_64-unknown-linux-gnu-library/3.3")

library(ReactomePA)

library(DOSE)

library(clusterProfiler)

library(biomaRt)

require(biomaRt)

#Down<-ressig[which(ressig$log2FoldChange<0),"genenames"]

#Up<-ressig[which(ressig$log2FoldChange>0),"genenames"]

#interesting_genes

Down<-mean_data_filtered[which(mean_data_filtered$log2foldchange_wrt_good<0),"genename"]

Up<-mean_data_filtered[which(mean_data_filtered$log2foldchange_wrt_good>0),"genename"]

#ensembl <- useMart("ENSEMBL_MART_ENSEMBL","hsapiens_gene_ensembl",

# host="www.ensembl.org") # <- No longer works ...

ensembl = useMart(biomart="ENSEMBL_MART_ENSEMBL", host="grch37.ensembl.org", path="/biomart/martservice" ,dataset="hsapiens_gene_ensembl")

#https://support.bioconductor.org/p/62064/

#listAttributes(ensembl)

filters<-listFilters(ensembl)

#grep('gene',filters$name,value=TRUE, ignore.case=TRUE)

getEntrezIDs<-function (geneNames)

{

tmp <- getBM(attributes = c("entrezgene"), filters = "hgnc_symbol", values = geneNames, mart = ensembl)

tmp

}

library(org.Hs.eg.db)

require(org.Hs.eg.db)

doGO<-function(title,geneNames,ensembl){

#ensembl = useMart(biomart="ENSEMBL_MART_ENSEMBL", host="grch37.ensembl.org", path="/biomart/martservice" ,dataset="hsapiens_gene_ensembl")

#https://support.bioconductor.org/p/62064/

filters<-listFilters(ensembl)

sigEntrez<-getEntrezIDs(geneNames)

sigEZ<-strsplit(as.character(sigEntrez), ", ")

sigEZ<-unlist(sigEZ)

goBP<-enrichGO(gene=sigEZ,'org.Hs.eg.db',ont="BP",pvalueCutoff=0.05,readable=T)

goMF<-enrichGO(gene=sigEZ,'org.Hs.eg.db',ont="MF",pvalueCutoff=0.05,readable=T)

goCC<-enrichGO(gene=sigEZ,'org.Hs.eg.db',ont="CC",pvalueCutoff=0.05,readable=T)

makeGOplot<-function(goobj,catnum,p_cutoff){

res<-goobj@result

res<-res[which(res$p.adjust<p_cutoff),]

res<-res[order(res$p.adjust),]

res$Description<-factor(res$Description,levels=res$Description)

if(dim(res)[1]>catnum){res<-res[1:catnum,]}

ggplot(res,aes(Description,-log10(p.adjust)))+geom_bar(stat="identity")+theme_bw()+theme(axis.text.x=element_text(angle=-90, hjust=0))

#ggplot(res,aes(Description,Count))+geom_bar(stat="identity")+theme_bw()+theme(axis.text.x=element_text(angle=-90, hjust=0))

}

A<-makeGOplot(goBP,20,0.05)+ggtitle(paste("GO BP",title,sep = " "))

B<-makeGOplot(goMF,20,0.05)+ggtitle(paste("GO MF",title,sep = " "))

C<-makeGOplot(goCC,20,0.05)+ggtitle(paste("GO CC",title,sep = " "))

#write.table(goBP@result,file=paste(filename,"_goBP_result.tab",sep="\t"),quote=FALSE)

#write.table(goMF@result,paste(filename,"_goMF_result.tab",sep="\t"),quote=FALSE)

#write.table(goCC@result,paste(filename,"_goCC_result.tab",sep="\t"),quote=FALSE)

goBPres<-goBP@result

goMFres<-goMF@result

goCCres<-goCC@result

return(list(A,B,C,goBPres,goMFres,goCCres))

}

DOWN_outputList<-doGO("Down Morphology DE", Down,ensembl)

DOWN_outputList[[1]] #oxygen and detox etc

DOWN_outputList[[2]] #oxygen transporter (from full list) #same + perixodase from filtered list

DOWN_outputList[[3]]#nothing #hemoglobin complex and ribosome

Down_goBP_result<-DOWN_outputList[[4]]

Down_goMF_result<-DOWN_outputList[[5]]#HBA2/HBA1 - DOWN IN GOOD

Down_goCC_result<-DOWN_outputList[[6]]#HBA2/HBA1

pdf("morphology_goBP_down_oxygentransport_filteredgenelist_originalDiff.pdf")

DOWN_outputList[[1]]

dev.off()

pdf("morphology_goMF_down_filteredgenelist_originalDiff.pdf")

DOWN_outputList[[2]]

dev.off()

pdf("morphology_goCC_down_filteredgenelist_originalDiff.pdf")

DOWN_outputList[[3]]

dev.off()

#pdf("morphology_goBP_down_oxygentransport_filteredgenelist.pdf")

DOWN_outputList[[1]]

#dev.off()

#pdf("morphology_goMF_down_filteredgenelist.pdf")

DOWN_outputList[[2]]

#dev.off()

#pdf("morphology_goCC_down_filteredgenelist.pdf")

DOWN_outputList[[3]]

#dev.off()

#down_genes_tpms<-data_to_plot[data_to_plot$genename%in%Down,]

#ggplot(down_genes_tpms,aes(MorphoGradeCall,TPM,colour=MorphoGradeCall))+geom_jitter()+theme_bw()+facet_wrap(~genename,scales="free")+scale_colour_manual(values=c("Bad"="gray","Good"="black"))

##pdf("morphology_down_goMF.pdf")

#DOWN_outputList[[2]] #use this!

##dev.off()

#jitter plots of the genes contributing to this go enrichment

genesofinterest<-Down_goBP_result$geneID[1]

genesofinterest_list<-unlist(str_split(genesofinterest,"/"))

genesofinterest_genes<-expression_annot_dt[grep(paste(genesofinterest_list,collapse="|"),expression_annot_dt$genename),]

#pdf("go_driving_genes_down_morphology_filteredlist_jitter.pdf")

ggplot(genesofinterest_genes,aes(MorphoGradeCall,TPM,colour=MorphoGradeCall))+geom_jitter()+theme_bw()+facet_wrap(~genename,scales="free")+scale_color_manual(values=c("Bad"="Red","Good"="Grey"))#+geom_text(aes(label=ReadableEmbryoID))

#dev.off()

##pdf("go_driving_genes_down_morphology_filteredlist_boxplot.pdf")

ggplot(genesofinterest_genes,aes(MorphoGradeCall,TPM,colour=MorphoGradeCall))+geom_boxplot(notch=TRUE)+theme_bw()+facet_wrap(~genename,scales="free")+scale_color_manual(values=c("Bad"="grey","Good"="Red"))#+geom_text(aes(label=ReadableEmbryoID))

##dev.off()

#ggplot(genesofinterest_genes,aes(MorphoGradeCall,TPM,colour=MorphoGradeCall))+geom_boxplot(notch=TRUE)+theme_bw()+facet_wrap(~genename,scales="free")

##pdf("morphology_down_goMFgenes.pdf")

#ggplot(genesofinterest_genes,aes(MorphoGradeCall,TPM,colour=MorphoGradeCall))+geom_jitter()+theme_bw()+facet_wrap(~genename,scales="free")+geom_text(aes(label=ReadableEmbryoID))

##dev.off()

#up in good

UP_outputList<-doGO("UP Morphology DE", Up,ensembl)

UP_outputList[[1]]#nothing

UP_outputList[[2]] # chloride, sodium, calcium channels

#UP_outputList[[3]]#nothing

UP_goMF_result<-UP_outputList[[5]]

genesofinterest<-unique(UP_goMF_result$geneID)

genesofinterest_list<-unlist(str_split(genesofinterest,"/"))

genesofinterest_genes<-expression_annot_dt[grep(paste(genesofinterest_list,collapse="|"),expression_annot_dt$genename),]

ggplot(genesofinterest_genes,aes(MorphoGradeCall,TPM,colour=MorphoGradeCall))+geom_jitter()+theme_bw()+facet_wrap(~genename,scales="free")+geom_text(aes(label=ReadableEmbryoID))

ggplot(genesofinterest_genes,aes(MorphoGradeCall,TPM,colour=MorphoGradeCall))+geom_boxplot(notch=TRUE)+theme_bw()+facet_wrap(~genename,scales="free")

#individual lists are more interesting

#ALL_outputList<-doGO("ALL Morphology DE", c(Down,Up),ensembl)

#ALL_outputList[[1]] #nothing

#ALL_outputList[[2]] #not that interesting

#ALL_outputList[[3]] #not that interesting

detach("package:biomaRt")

```

Morphological DE GSEA

##GSEA setup

```{r gsea_diffs, include=FALSE}

library(GSA)

library(limma)

library(gplots)

library(marray)

library(RColorBrewer)

library(RMySQL)

#reactome_gs <- GSA.read.gmt("/n/rinn_data2/users/agroff/GSEA/c2.cp.reactome.v4.0.symbols.gmt")

#biocarta_gs <- GSA.read.gmt("/n/rinn_data2/users/agroff/GSEA/c2.cp.biocarta.v4.0.symbols.gmt")

reactome_gs <- GSA.read.gmt("/Volumes/valor2/users/agroff/GSEA/c2.cp.reactome.v4.0.symbols.gmt")

biocarta_gs <- GSA.read.gmt("/Volumes/valor2/users/agroff/GSEA/c2.cp.biocarta.v4.0.symbols.gmt")

res<-as.data.frame(res)

geneinfo<-row.names(res)

res$info<-geneinfo

geneinfo2<-str_split_fixed(geneinfo,"_",2)

res$txname<-geneinfo2[,1]

res$genename<-geneinfo2[,2]

dat<-merge(res,txn_chr_info,by.x="txname","TXNAME")

dat<-dat[order(dat$stat),]

gene_set_index <- function(genelist, short_names){

which(short_names %in% genelist)

}

get_gene_set_p_vals <- function(input, gs, alternative){

gene_set_indices <- lapply(gs$genesets, function(genelist){

gene_set_index(genelist,input$short_name)

})

pvl<-lapply(gene_set_indices,geneSetTest,input$test_stat, alternative=alternative)

pvl_mat<-as.data.frame(t(unlist(pvl)))

colnames(pvl_mat) <- gs$geneset.names

return(pvl_mat)

}

get_gene_set_q_vals <- function(pvl_mat, method="bonferroni"){

comp_corrected <- matrix(p.adjust(pvl_mat, method=method), nrow=nrow(pvl_mat), ncol=ncol(pvl_mat))

colnames(comp_corrected) <- colnames(pvl_mat)

rownames(comp_corrected) <- rownames(pvl_mat)

return(comp_corrected)

}

colMins<-function(x){

apply(x,2,min)

}

rowMins<-function(x){

apply(x,1,min)

}

InputCols<-maPalette(low="white",high="red",k=100)

ztest<-function(samp,pop){

(mean(samp,na.rm=T)-mean(pop,na.rm=T))/sd(pop,na.rm=T)

}

get_gene_set_ztest <- function(scoring_df, gs){

gene_set_indices <- lapply(gs$genesets, function(genelist){

gene_set_index(genelist, scoring_df$short_name)

})

zscores <- lapply(gene_set_indices,function(gsi){

ztest(scoring_df$test_stat[gsi],scoring_df$test_stat)

})

zscore_mat<-do.call(rbind,lapply(zscores,unlist))

rownames(zscore_mat) <- gs$geneset.names

colnames(zscore_mat) <- "zscore"

return(zscore_mat)

}

```

## GSEA computation

```{r gsea, include=FALSE}

df.pop<-data.frame("short_name"=toupper(dat$GENEID),"test_stat"=dat$stat)

df.pop.unique<-unique(df.pop)

rownames(df.pop.unique)<-NULL

df.pop.unique.ordered<-df.pop.unique[order(df.pop.unique$test_stat),]

Input.df<-df.pop.unique.ordered

Input.df$short_name<-as.character(Input.df$short_name)

gseaInput.df<-Input.df

gseaInput.df$test_stat[which(is.na(gseaInput.df$test_stat))]<-0

reactome_pvl_mat <- get_gene_set_p_vals(Input.df, reactome_gs,alternative="either")

reactome_pvl_corrected <- get_gene_set_q_vals(reactome_pvl_mat)

reactome_pvl_corrected<-rbind(reactome_pvl_corrected,reactome_pvl_corrected)

biocarta_pvl_mat <- get_gene_set_p_vals(Input.df, biocarta_gs, alternative="either")

biocarta_pvl_corrected <- get_gene_set_q_vals(biocarta_pvl_mat)

biocarta_pvl_corrected<-rbind(biocarta_pvl_corrected,biocarta_pvl_corrected)

reactome_zscores<-get_gene_set_ztest(Input.df,reactome_gs)

reactome_zscores<-cbind(reactome_zscores,reactome_zscores)

biocarta_zscores<-get_gene_set_ztest(Input.df,biocarta_gs)

biocarta_zscores<-cbind(biocarta_zscores,biocarta_zscores)

k <- 100

myColors<-maPalette(low="blue",mid="white",high="red",k=k)

myBreaks<-seq(-2,2,length.out=(k+1))

enrichmentBreaks<-seq(0,6,length.out=(k+1))

```

## GSEA figures

Biocarta zscore:

```{r gsea_zscore_biocarta, fig.height=8, fig.width=8}

x<-((biocarta_zscores[which(colMins(biocarta_pvl_corrected) < 0.01),]))

x_ordered<-x[order(x[,1], decreasing=TRUE),]

if(length(x_ordered)>50){x_ordered<-x_ordered[1:50,]}

x_ordered<-as.matrix(x_ordered)

if(dim(x_ordered)[1]>1){

heatmap.2(x_ordered, trace="none",col=myColors,breaks=myBreaks,margins=c(1,20),dendrogram="both",labCol=c(""),cexRow =1, offsetRow=0)

}else{print("Not enough significant categories to print a heatmap!")}

rm(x_ordered)

rm(x)

```

Reactome zscore:

```{r gsea_zscore_reactome, fig.height=8, fig.width=8}

x<-((reactome_zscores[which(colMins(reactome_pvl_corrected) < 0.01),]))

x_ordered<-x[order(x[,1], decreasing=TRUE),]

if(length(x_ordered)>50){x_ordered<-x_ordered[1:50,]}

x_ordered<-as.matrix(x_ordered)

if(dim(x_ordered)[1]>1){

pdf("Morphology_AAvCC_reactome_gsa.pdf")

heatmap.2(x_ordered, trace="none",col=myColors,breaks=myBreaks,margins=c(1,20),dendrogram="both",labCol=c(""),cexRow =1, offsetRow=0)

dev.off()

}else{print("Not enough significant categories to print a heatmap!")}

rm(x_ordered)

rm(x)

```

Replicate scatters:

```{r repscatters}

tpm_files<-lapply(diff_files,read.table,header=TRUE)

expression_info<-lapply(tpm_files,function(x){

newx<-x[,c("gene_id","TPM")]

newx

})

exp_info2<-lapply(seq_along(expression_info),function(x){

new<-expression_info[[x]]

new$samplename<-names(expression_info)[x]

as.data.frame(new)

})

expression_info<-do.call("rbind",exp_info2)

expression_info$genename<-str_split_fixed(expression_info$gene_id,"_",2)[,2]

exp_info_wide<-dcast(expression_info,gene_id+genename~samplename,value.var="TPM")

library(GGally)

ggpairs(exp_info_wide[,3:8]) #currently only 5 genes--need to read in ALL!

```

#Morphokinetic

Day (6 v 7) may be a confounding factor? but difficult to say and we're limited by sample availability... sex may also be confounding..

# Setup

```{r knitropts}

knitr::opts_chunk$set(echo=FALSE, message=FALSE, warning=FALSE,fig.path = "/ploidy_images/", dev=c('png', 'pdf'))

```

```{r setup}

library(DESeq2)

library(tximport)

library(readr)

library(stringr)

library(ggplot2)

library(reshape2)

library(plyr)

library(dplyr)

rsem_count_dir<-"/Volumes/valor2/users/agroff/seq/humanEmbryo/quants/rsem/50bptrim"

#make transcript to gene conversion file using dat1 from above

files <- list.files(rsem_count_dir,pattern="*.genes.results",full.names=TRUE)

filenames<-list.files(rsem_count_dir,pattern="*.genes.results")

filenames<-gsub(".genes.results","",filenames)

names(files) <- filenames

metadata<-read.table("/Volumes/valor2/users/agroff/seq/humanEmbryo/analysis/061917_updated_metadata.txt",header=TRUE,stringsAsFactors = FALSE)

annot_short<-read.table("/Volumes/valor2/users/agroff/seq/humanEmbryo/analysis/embryo_transcript_annotation_gencodev19_hg19.tab")

tx2gene<-read.table("/Volumes/valor2/users/agroff/seq/humanEmbryo/analysis/tx2gene.tab",header=TRUE)

txn_chr_info<-read.table("/Volumes/valor2/users/agroff/seq/humanEmbryo/analysis/txn_chr_info.tab",header=TRUE)

chr_sizes<-read.table("/Volumes/valor2/users/agroff/seq/humanEmbryo/analysis/deseq_all_old/chrom_sizes.tab",header=TRUE)

potentially_tetraploid<-c("NRAG41","NRAG78","NRAG82","NRAG90","NRAG93","NRAG95","NRAG66")

metadata<-metadata[!metadata$ProcessingID%in%potentially_tetraploid,]

```

# Define and load samples

# Morphokinetic

```{r defineMorpho}

morphosamples<-metadata[grep("GOOD|BAD",metadata$MorphokineticCall),]

morphosamples<-morphosamples[grep("wholeembryo",morphosamples$Sampletype),]

diff_files<-files[which(names(files)%in%morphosamples$ProcessingID)]

diff_metadata<-metadata[which(metadata$ProcessingID%in%names(diff_files)),]

txi <- tximport(diff_files, type="rsem", tx2gene=tx2gene, reader=read_tsv)

rownames(diff_metadata)<-diff_metadata$ProcessingID

diff_metadata$MorphokineticCall<-factor(diff_metadata$MorphokineticCall)

txi$length<-txi$length+1

ddsTxi <- DESeqDataSetFromTximport(txi, colData=diff_metadata,design=~MorphokineticCall)

```

# Morphokinetic Quality Filter

Remove genes that are not expressed in any sample

- can add binary screening so must be expressed >1 in any one sample

```{r quality_filter}

expressed_genes<-read.table("/Volumes/valor2/users/agroff/seq/humanEmbryo/analysis/March2018_expressed_genes.tab",header=TRUE)

expressed_genes<-expressed_genes$x

samples_keep<-read.table("/Volumes/valor2/users/agroff/seq/humanEmbryo/analysis/March2018_samples_passing_QC.tab",header=TRUE)

#quality filtering

#remove genes with no counts

dds<-ddsTxi

nrow(dds)

dds <- dds[ rowSums(counts(dds)) > 1, ] #get rid of super noisy expression

nrow(dds)

dim(dds)

dds<-dds[,(colnames(dds)%in%samples_keep$ProcessingID)] #get rid of low expressing samples

dim(dds)

#keep only remaining expressed genes (>=1TPM in at least 1 sample)

dds<-dds[row.names(dds)%in%expressed_genes,]

#remove mitochondrial reads here

counttable<-as.data.frame(counts(dds))

counttable$geneinfo<-row.names(counttable)

counttable$txname<-str_split_fixed(counttable$geneinfo,"_",2)[,1]

counttable$genename<-str_split_fixed(counttable$geneinfo,"_",2)[,2]

counttable_chr<-merge(counttable,txn_chr_info,by.x="txname",by.y="TXNAME")

mito<-counttable_chr[which(counttable_chr$chr=="chrM"),]

counttable<-counttable_chr[which(counttable_chr$chr!="chrM"),]

genomic_genes<-counttable$geneinfo

dds<-dds[row.names(dds)%in%genomic_genes,]

#remove PAR1 genes

par1<-c("PLCXD1","GTPBP6","PPP2R3B","SHOX","CRLF2","CSF2RA","IL3RA","SLC25A6","ASMTL","P2RY8","CXYorf3","ASMT","DHRSXY","ZBED1","CD99","XG")

par1_genes<-counttable[which(counttable$genename %in% par1),"geneinfo"]

dds<-dds[!(row.names(dds)%in%par1_genes),]

dim(dds)

#update diff_files after QC

diff_files<-diff_files[names(diff_files)%in%dds@colData$ProcessingID]

```

PCA morpho samples

```{r MorphokineticPCA}

vsd<-varianceStabilizingTransformation(dds)

par(mfrow=c(1, 2))

dds <- estimateSizeFactors(dds)

plotPCA(vsd, intgroup = c("MorphokineticCall"))+ggtitle("PCA, MorphokineticCall")+theme_bw()

```

Differential expression morphological grade

```{r DiffExp}

dds<-DESeq(dds)

resultsNames(dds)

res<-results(dds,alpha=0.05)

ressig<-as.data.frame(res[which(res$padj<0.05),])

ressig$info<-row.names(ressig)

ressig$genenames<-str_split_fixed(ressig$info,"_",2)[,2]

ressig$txname<-str_split_fixed(ressig$info,"_",2)[,1]

comb_dat<-as.data.frame(merge(ressig,annot_short,by.x="txname","txname"))

dat<-as.data.frame(res)

dat$sig<-"no"

dat$sig[which(dat$padj<0.05)]<-"yes"

dat$info<-row.names(dat)

dat$genenames<-str_split_fixed(dat$info,"_",2)[,2]

dat$txname<-str_split_fixed(dat$info,"_",2)[,1]

#write.table(dat,file="morphokinetic_diff.txt",sep="\t",quote=FALSE,row.names=FALSE,col.names=TRUE)

```

Morphokinetic DE volcano

```{r MorphokineticDE}

pdf("Morphokinetic_DE_volcano_all.pdf")

#ggplot(dat,aes(log2FoldChange,-log10(pvalue),colour=sig))+geom_point()+theme_bw()+scale_colour_manual(values=c("no"="black","yes"="red"))

ggplot(dat,aes(log2FoldChange,-log10(pvalue),label=genenames))+geom_bin2d()+theme_bw()+scale_fill_gradient("low"="light gray","high"="black")+geom_point(data=subset(dat,sig=="yes"),aes(log2FoldChange,-log10(pvalue)),color="red")#+geom_text(data=subset(dat,sig=="yes"),color="red")

dev.off()

```

Morphological DE TPM-based gene expression jitter plots

```{r TPM_jitters}

tpm_files<-lapply(diff_files,read.table,header=TRUE)

expression_info<-lapply(tpm_files,function(x){

newx<-x[,c("gene_id","TPM")]

newx

})

exp_info2<-lapply(seq_along(expression_info),function(x){

new<-expression_info[[x]]

new$samplename<-names(expression_info)[x]

as.data.frame(new)

})

expression_info<-do.call("rbind",exp_info2)

expression_info$genename<-str_split_fixed(expression_info$gene_id,"_",2)[,2]

expression_info<-expression_info[which(expression_info$genename %in% ressig$genenames),]

expression_annot<-merge(expression_info,metadata,by.x="samplename",by.y="ProcessingID")

library(data.table)

expression_annot_dt<-data.table(expression_annot)

expression_annot_dt$infocondition<-paste(expression_annot_dt$gene_id,expression_annot_dt$MorphokineticCall,sep="_")

morphokinetic_diff_genes<-unique(expression_annot_dt$genename)

mean_by_condition<-aggregate(expression_annot_dt[,c("MorphokineticCall","genename","TPM")],by=list(expression_annot_dt$MorphokineticCall,expression_annot_dt$genename),mean)

mean_by_condition$MorphokineticCall<-NULL

mean_by_condition$genename<-NULL

names(mean_by_condition)<-c("MorphokineticCall","genename","MeanTPM")

mean_data<-reshape(mean_by_condition, idvar = "genename", timevar = "MorphokineticCall", ids="MeanTPM",direction = "wide")

mean_data$log2foldchange_wrt_good<-log2(mean_data$MeanTPM.GOOD/mean_data$MeanTPM.BAD)

mean_data_binary<-mean_data

mean_data_binary<-mean_data_binary[,c("MeanTPM.BAD","MeanTPM.GOOD")]

mean_data_binary[mean_data_binary<10]<-0

mean_data_binary[mean_data_binary>=10]<-1

binary_sums<-rowSums(mean_data_binary) #keep 1 or 2, get rid of 0

mean_data_filtered<-mean_data[which(binary_sums>0),]

pdf("morphokinetic_WE_all_jitter.pdf")

ggplot(expression_annot_dt,aes(MorphokineticCall,TPM,colour=MorphokineticCall))+geom_jitter()+theme_bw()+facet_wrap(~genename,scales="free")+scale_colour_manual(values=c("BAD"="red","GOOD"="gray"))#+geom_text(aes(label=ReadableEmbryoID))

dev.off()

```

Morphokinetic DE GO TERMS

# GO term analysis of sig DE genes

```{r go_sig_genes, eval=FALSE, include=FALSE}

#source("https://bioconductor.org/biocLite.R")

#biocLite("org.Hs.eg.db")#,lib="/n/home06/agroff/R/x86_64-unknown-linux-gnu-library/3.3")

library(ReactomePA)

library(DOSE)

library(clusterProfiler)

require(biomaRt)

Down<-ressig[which(ressig$log2FoldChange<0),"genenames"]

Up<-ressig[which(ressig$log2FoldChange>0),"genenames"]

#ensembl <- useMart("ENSEMBL_MART_ENSEMBL","hsapiens_gene_ensembl",

# host="www.ensembl.org") # <- No longer works ...

ensembl = useMart(biomart="ENSEMBL_MART_ENSEMBL", host="grch37.ensembl.org", path="/biomart/martservice" ,dataset="hsapiens_gene_ensembl")

#https://support.bioconductor.org/p/62064/

#listAttributes(ensembl)

filters<-listFilters(ensembl)

#grep('gene',filters$name,value=TRUE, ignore.case=TRUE)

getEntrezIDs<-function (geneNames)

{

tmp <- getBM(attributes = c("entrezgene"), filters = "hgnc_symbol", values = geneNames, mart = ensembl)

tmp

}

require(org.Hs.eg.db)

doGO<-function(title,geneNames,ensembl){

#ensembl = useMart(biomart="ENSEMBL_MART_ENSEMBL", host="grch37.ensembl.org", path="/biomart/martservice" ,dataset="hsapiens_gene_ensembl")

#https://support.bioconductor.org/p/62064/

filters<-listFilters(ensembl)

sigEntrez<-getEntrezIDs(geneNames)

sigEZ<-strsplit(as.character(sigEntrez), ", ")

sigEZ<-unlist(sigEZ)

goBP<-enrichGO(gene=sigEZ,'org.Hs.eg.db',ont="BP",pvalueCutoff=0.05,readable=T)

goMF<-enrichGO(gene=sigEZ,'org.Hs.eg.db',ont="MF",pvalueCutoff=0.05,readable=T)

goCC<-enrichGO(gene=sigEZ,'org.Hs.eg.db',ont="CC",pvalueCutoff=0.05,readable=T)

makeGOplot<-function(goobj,catnum,p_cutoff){

res<-goobj@result

res<-res[which(res$p.adjust<p_cutoff),]

res<-res[order(res$p.adjust),]

res$Description<-factor(res$Description,levels=res$Description)

if(dim(res)[1]>catnum){res<-res[1:catnum,]}

ggplot(res,aes(Description,-log10(p.adjust)))+geom_bar(stat="identity")+theme_bw()+theme(axis.text.x=element_text(angle=-90, hjust=0))

#ggplot(res,aes(Description,Count))+geom_bar(stat="identity")+theme_bw()+theme(axis.text.x=element_text(angle=-90, hjust=0))

}

A<-makeGOplot(goBP,20,0.05)+ggtitle(paste("GO BP",title,sep = " "))

B<-makeGOplot(goMF,20,0.05)+ggtitle(paste("GO MF",title,sep = " "))

C<-makeGOplot(goCC,20,0.05)+ggtitle(paste("GO CC",title,sep = " "))

#write.table(goBP@result,file=paste(filename,"_goBP_result.tab",sep="\t"),quote=FALSE)

#write.table(goMF@result,paste(filename,"_goMF_result.tab",sep="\t"),quote=FALSE)

#write.table(goCC@result,paste(filename,"_goCC_result.tab",sep="\t"),quote=FALSE)

goBPres<-goBP@result

goMFres<-goMF@result

goCCres<-goCC@result

return(list(A,B,C,goBPres,goMFres,goCCres))

}

#DOWN_outputList<-doGO("Down Morphokinetic DE", Down,ensembl)

#DOWN_outputList[[1]] #nothing

#DOWN_outputList[[2]] #nothing

#DOWN_outputList[[3]] #nothing

UP_outputList<-doGO("UP Morphokinetic DE", Up,ensembl)

UP_outputList[[1]]

UP_outputList[[2]]

UP_outputList[[3]]#none

UP_goBP_result<-UP_outputList[[4]]

UP_goMF_result<-UP_outputList[[5]]

UP_goCC_result<-UP_outputList[[6]]

pdf("morphokinetic_goBP_all.pdf")

UP_outputList[[1]]

dev.off()

pdf("morphokinetic_goMF_all.pdf")

UP_outputList[[2]]

dev.off()

pdf("morphokinetic_goCC_all.pdf")

UP_outputList[[3]]

dev.off()

genesofinterest<-UP_goMF_result$geneID

genesofinterest_list<-unique(unlist(str_split(genesofinterest,"/")))

genesofinterest_genes<-expression_annot_dt[grep(paste(genesofinterest_list,collapse="|"),expression_annot_dt$genename),]

pdf("go_driving_genes_up_morphokinetic_all_jitter.pdf")

ggplot(genesofinterest_genes,aes(MorphokineticCall,TPM,colour=MorphokineticCall))+geom_jitter()+theme_bw()+facet_wrap(~genename,scales="free")+scale_color_manual(values=c("BAD"="Red","GOOD"="Grey"))#+geom_text(aes(label=ReadableEmbryoID))

dev.off()

detach("package:biomaRt")

```

Morphokinetic GSEA - not super interesting..

##GSEA setup

```{r gsea_diffs, include=FALSE}

library(GSA)

library(limma)

library(gplots)

library(marray)

library(RColorBrewer)

library(RMySQL)

reactome_gs <- GSA.read.gmt("/Volumes/valor2/users/agroff/GSEA/c2.cp.reactome.v4.0.symbols.gmt")

biocarta_gs <- GSA.read.gmt("/Volumes/valor2/users/agroff/GSEA/c2.cp.biocarta.v4.0.symbols.gmt")

res<-as.data.frame(res)

geneinfo<-row.names(res)

res$info<-geneinfo

geneinfo2<-str_split_fixed(geneinfo,"_",2)

res$txname<-geneinfo2[,1]

res$genename<-geneinfo2[,2]

dat<-merge(res,txn_chr_info,by.x="txname","TXNAME")

dat<-dat[order(dat$stat),]

gene_set_index <- function(genelist, short_names){

which(short_names %in% genelist)

}

get_gene_set_p_vals <- function(input, gs, alternative){

gene_set_indices <- lapply(gs$genesets, function(genelist){

gene_set_index(genelist,input$short_name)

})

pvl<-lapply(gene_set_indices,geneSetTest,input$test_stat, alternative=alternative)

pvl_mat<-as.data.frame(t(unlist(pvl)))

colnames(pvl_mat) <- gs$geneset.names

return(pvl_mat)

}

get_gene_set_q_vals <- function(pvl_mat, method="bonferroni"){

comp_corrected <- matrix(p.adjust(pvl_mat, method=method), nrow=nrow(pvl_mat), ncol=ncol(pvl_mat))

colnames(comp_corrected) <- colnames(pvl_mat)

rownames(comp_corrected) <- rownames(pvl_mat)

return(comp_corrected)

}

colMins<-function(x){

apply(x,2,min)

}

rowMins<-function(x){

apply(x,1,min)

}

InputCols<-maPalette(low="white",high="red",k=100)

ztest<-function(samp,pop){

(mean(samp,na.rm=T)-mean(pop,na.rm=T))/sd(pop,na.rm=T)

}

get_gene_set_ztest <- function(scoring_df, gs){

gene_set_indices <- lapply(gs$genesets, function(genelist){

gene_set_index(genelist, scoring_df$short_name)

})

zscores <- lapply(gene_set_indices,function(gsi){

ztest(scoring_df$test_stat[gsi],scoring_df$test_stat)

})

zscore_mat<-do.call(rbind,lapply(zscores,unlist))

rownames(zscore_mat) <- gs$geneset.names

colnames(zscore_mat) <- "zscore"

return(zscore_mat)

}

```

## GSEA computation

```{r gsea, include=FALSE}

df.pop<-data.frame("short_name"=toupper(dat$GENEID),"test_stat"=dat$stat)

df.pop.unique<-unique(df.pop)

rownames(df.pop.unique)<-NULL

df.pop.unique.ordered<-df.pop.unique[order(df.pop.unique$test_stat),]

Input.df<-df.pop.unique.ordered

Input.df$short_name<-as.character(Input.df$short_name)

gseaInput.df<-Input.df

gseaInput.df$test_stat[which(is.na(gseaInput.df$test_stat))]<-0

reactome_pvl_mat <- get_gene_set_p_vals(Input.df, reactome_gs,alternative="either")

reactome_pvl_corrected <- get_gene_set_q_vals(reactome_pvl_mat)

reactome_pvl_corrected<-rbind(reactome_pvl_corrected,reactome_pvl_corrected)

biocarta_pvl_mat <- get_gene_set_p_vals(Input.df, biocarta_gs, alternative="either")

biocarta_pvl_corrected <- get_gene_set_q_vals(biocarta_pvl_mat)

biocarta_pvl_corrected<-rbind(biocarta_pvl_corrected,biocarta_pvl_corrected)

reactome_zscores<-get_gene_set_ztest(Input.df,reactome_gs)

reactome_zscores<-cbind(reactome_zscores,reactome_zscores)

biocarta_zscores<-get_gene_set_ztest(Input.df,biocarta_gs)

biocarta_zscores<-cbind(biocarta_zscores,biocarta_zscores)

k <- 100

myColors<-maPalette(low="blue",mid="white",high="red",k=k)

myBreaks<-seq(-2,2,length.out=(k+1))

enrichmentBreaks<-seq(0,6,length.out=(k+1))

```

## GSEA figures

Biocarta zscore:

```{r gsea_zscore_biocarta, fig.height=8, fig.width=8}

x<-((biocarta_zscores[which(colMins(biocarta_pvl_corrected) < 0.01),]))

x_ordered<-x[order(x[,1], decreasing=TRUE),]

if(length(x_ordered)>50){x_ordered<-x_ordered[1:50,]}

x_ordered<-as.matrix(x_ordered)

if(dim(x_ordered)[1]>1){

heatmap.2(x_ordered, trace="none",col=myColors,breaks=myBreaks,margins=c(1,20),dendrogram="both",labCol=c(""),cexRow =1, offsetRow=0)

}else{print("Not enough significant categories to print a heatmap!")}

rm(x_ordered)

rm(x)

```

Reactome zscore:

```{r gsea_zscore_reactome, fig.height=8, fig.width=8}

x<-((reactome_zscores[which(colMins(reactome_pvl_corrected) < 0.01),]))

x_ordered<-x[order(x[,1], decreasing=TRUE),]

if(length(x_ordered)>50){x_ordered<-x_ordered[1:50,]}

x_ordered<-as.matrix(x_ordered)

if(dim(x_ordered)[1]>1){

heatmap.2(x_ordered, trace="none",col=myColors,breaks=myBreaks,margins=c(1,20),dendrogram="both",labCol=c(""),cexRow =1, offsetRow=0)

}else{print("Not enough significant categories to print a heatmap!")}

rm(x_ordered)

rm(x)

```

Replicate scatters:

```{r repscatters}

tpm_files<-lapply(diff_files,read.table,header=TRUE)

expression_info<-lapply(tpm_files,function(x){

newx<-x[,c("gene_id","TPM")]

newx

})

exp_info2<-lapply(seq_along(expression_info),function(x){

new<-expression_info[[x]]

new$samplename<-names(expression_info)[x]

as.data.frame(new)

})

expression_info<-do.call("rbind",exp_info2)

expression_info$genename<-str_split_fixed(expression_info$gene_id,"_",2)[,2]

exp_info_wide<-dcast(expression_info,gene_id+genename~samplename,value.var="TPM")

library(GGally)

ggpairs(exp_info_wide[,3:8]) #currently only 5 genes--need to read in ALL!

```

Morphokinetic data Fig

```{r setup}

library(plyr)

library(reshape2)

library(ggplot2)

library(stringr)

```

```{r loaddata}

dat<-read.table("~/Google Drive/Embryo/Info:Data/morphokinetic_data_050517.tab",sep="\t",header=TRUE)

#2316Embryo1 (E1), 2316Embryo3 (E2), 2316Embryo5 (E3), 2316Embryo12(E4), 21716Embryo4 (E5), 21716Embryo7 (E6), 21716Embryo8 (E7), 21716Embryo13 (E8), 21716Embryo15 (E10), 21716Embryo20 (E11)

dat$PaperID<-"NA"

dat$PaperID[which(dat$Sample=="2316Embryo1")]<-"E1"

dat$PaperID[which(dat$Sample=="2316Embryo3")]<-"E2"

dat$PaperID[which(dat$Sample=="2316Embryo5")]<-"E3"

dat$PaperID[which(dat$Sample=="2316Embryo12")]<-"E4"

dat$PaperID[which(dat$Sample=="21716Embryo4")]<-"E5"

dat$PaperID[which(dat$Sample=="21716Embryo7")]<-"E6"

dat$PaperID[which(dat$Sample=="21716Embryo8")]<-"E7"

dat$PaperID[which(dat$Sample=="21716Embryo13")]<-"E8"

dat$PaperID[which(dat$Sample=="21716Embryo15")]<-"E10"

dat$PaperID[which(dat$Sample=="21716Embryo20")]<-"E11"

dat.melt<-melt(dat)

dat.melt$type<-"embryos"

dat.melt$type[grep("clin",dat.melt$Sample)]<-"clinical"

#metadata<-read.table("/Volumes/valor2/users/agroff/seq/humanEmbryo/analysis/March2018_paperID_updated_metadata.txt",header=TRUE,stringsAsFactors = FALSE)

```

plot

```{r plotdata}

#pdf("morphokinetic_boxplots.pdf")

#ggplot(dat.melt,aes(variable,value,color=type))+geom_boxplot(notch=TRUE)+theme_bw()+ylim(0,125)

#dev.off()

dat.melt$label<-"undet"

dat.melt$label[which(dat.melt$Grade=="GOOD")]<-"GOOD"

dat.melt$label[which(dat.melt$Grade=="BAD")]<-"BAD"

dat.melt$label[which(dat.melt$type=="clinical")]<-"clinical"

#pdf("morphokinetic_dotplots.pdf")

#ggplot(dat.melt,aes(variable,value,color=type))+geom_point(alpha=0.5)+theme_bw()+ylim(0,125)+scale_colour_manual(values=c("clinical"="grey","embryos"="black"))

#dev.off()

#ggplot(dat.melt,aes(variable,value,color=label))+geom_point(alpha=0.5)+theme_bw()+ylim(0,125)+scale_colour_manual(values=c("clinical"="grey","GOOD"="green","BAD"="red","undet"="black"))

#ggplot(dat.melt,aes(variable,value,color=label))+geom_boxplot()+theme_bw()+ylim(0,125)+scale_colour_manual(values=c("clinical"="grey","GOOD"="green","BAD"="red","undet"="black"))

dat.melt2<-dat.melt[grep("undet",dat.melt$label,invert=TRUE),]

#ggplot(dat.melt2,aes(variable,value,color=label))+geom_point(alpha=0.5)+theme_bw()+ylim(0,125)+scale_colour_manual(values=c("clinical"="grey","GOOD"="red","BAD"="black"))

#pdf("morph_jitter_dotplot.pdf")

ggplot(dat.melt2,aes(variable,value,color=label))+geom_jitter()+theme_bw()+ylim(0,125)+scale_colour_manual(values=c("clinical"="grey","GOOD"="red","BAD"="black"))

#dev.off()

#ggplot(dat.melt2,aes(variable,value,color=label))+geom_boxplot()+theme_bw()+ylim(0,125)+scale_colour_manual(values=c("clinical"="grey","GOOD"="red","BAD"="black"))

#ggplot(dat.melt,aes(variable,value,group=Sample,colour=type))+geom_line()+geom_point()+theme_bw()

graded<-dat.melt[!is.na(dat.melt$Grade),]

graded$label<-paste(graded$Sample,graded$Grade,sep="_")

#pdf("graded_lineplots.pdf")

#ggplot(graded,aes(variable,value,group=Sample,colour=label))+geom_line()+geom_point()+theme_bw()+ylim(0,125)

#dev.off()

examples<-graded[which(graded$Sample%in%c("2316Embryo12","21716Embryo4","2316Embryo5","21716Embryo7")),]

#pdf("graded_lineplot_examples.pdf")

#ggplot(examples,aes(variable,value,group=Sample,colour=label))+geom_line()+geom_point()+theme_bw()+ylim(0,125)

#dev.off()

```

```{r final}

pdf("morphokinetic_data_overview.pdf")

ggplot(dat.melt2,aes(variable,value,color=label,group=PaperID,label=PaperID))+geom_jitter()+theme_bw()+ylim(0,125)+scale_colour_manual(values=c("clinical"="grey","GOOD"="black","BAD"="red"))+geom_line(data=subset(dat.melt2,type!="clinical"))+geom_text(data=subset(dat.melt2,type!="clinical"))+theme(axis.text.x=element_text(angle=90, hjust=0))

dev.off()

```
